# Diverged Plant Terpene Synthases Reroute the Carbocation Cyclization Path towards the Formation of Unprecedented 6/11/5 and 6/6/7/5 Sesterterpene Scaffolds

**DOI:** 10.1002/anie.201711444

**Published:** 2017-12-28

**Authors:** Ancheng C. Huang, Young J. Hong, Andrew D. Bond, Dean J. Tantillo, Anne Osbourn

**Affiliations:** ^1^ Department of Metabolic Biology John Innes Centre Colney Lane, Norwich Research Park Norwich NR4 7UH UK; ^2^ Department of Chemistry University of California, Davis Davis CA 95616 USA; ^3^ Department of Chemistry University of Cambridge Lensfield Rd Cambridge CB2 1EW UK

**Keywords:** cyclization, natural products, quantum chemical calculations, sesterterpene biosynthesis, terpene synthases

## Abstract

Sesterterpenoids are a relatively rare class of plant terpenes. Sesterterpene synthase (STS)‐mediated cyclization of the linear C_25_ isoprenoid precursor geranylfarnesyl diphosphate (GFPP) defines sesterterpene scaffolds. So far only a very limited number of STSs have been characterized. The discovery of three new plant STSs is reported that produce a suite of sesterterpenes with unprecedented 6/11/5 and 6/6/7/5 fused ring systems when transiently co‐expressed with a GFPP synthase in Nicotiana benthamiana. Structural elucidation, feeding experiments, and quantum chemical calculations suggest that these STSs catalyze an unusual cyclization path involving reprotonation, intramolecular 1,6 proton transfer, and concerted but asynchronous bicyclization events. The cyclization is diverted from those catalyzed by the characterized plant STSs by forming unified 15/5 bicyclic sesterterpene intermediates. Mutagenesis further revealed a conserved amino acid residue implicated in reprotonation.

Terpenoids are the largest class of natural products, with over 80 000 molecular structures discovered to date.^1^ The structural complexity and diversity of terpenoids is largely derived from the numerous carbocation cyclization modes of linear (C_5_ isoprene)_*n*_‐containing precursors, catalyzed by terpene synthases (TPSs). TPSs can be categorized into two classes (I and II) depending on the mode of initiation of cyclization of the linear precursor substrates. Class I TPSs are metal‐dependent enzymes that initiate cyclization of linear isoprenoid diphosphates via abstraction of the diphosphates for carbocation generation, whilst class II TPSs initiate catalysis by protonation of a C=C bond or epoxide group in an isoprenoid substrate.^1^ Structurally, an aspartate‐rich DDXXD motif and an NSE/DTE motif are typically present in class I TPSs, whereas a general acid motif DXDD is the signature of a class II TPS.^2^

Sesterterpenes, which are comprised of five C_5_ isoprene units, constitute a relatively rare subclass of terpenes. The majority characterized to date are from marine sponges and terrestrial fungi, with only about 80 structures reported from the plant kingdom so far.^3^ This group of terpenes thus represents an underexplored area of chemical space that holds considerable promise for discovery of new structurally complex and diverse compounds and potential new bioactivities.^4^ TPSs that catalyze sesterterpene formation are called sesterterpene synthases (STSs). They have been found in fungi, and more recently several examples have been found in plants, all of which are from the Brassicaceae family. Fungal STSs are bifunctional chimeric proteins that consist of a TPS and a *trans*‐prenytransferase (PT) domain; the latter synthesizes geranylfarnesyl diphosphate (GFPP), the substrate for TPS‐mediated cyclization, from dimethyl allyl diphosphate (DMAPP) and isopentenyl diphosphate (IPP).^5^ While STSs in plants have only a TPS domain, the genes that encode these STSs tend to be physically clustered with a PT gene in plant genomes.^3^, ^6^ This clustering phenomenon has enabled us to rapidly search available sequenced plant genomes for candidate STS genes using a customized algorithm (PlantiSMASH) designed to identify clustered biosynthetic genes in plant genomes.^3^, ^7^ Intriguingly, although fungal and plant STSs share about 10 % protein sequence identity and are phylogenetically distant, they can in some cases catalyze the conversion of GFPP to form very similar structures via a unified bicyclic C_12_ carbocation (**A1**), for example, (+)‐quiannulatene by fungal EvQS and (−)‐*ent*‐quiannulatene by plant AtTPS25 (Figure 1 and Figure 2).^3^, 5c,5d This suggests that the folds of the active site pockets of STSs from fungi and plants might be conserved to some extent, and that the ability to catalyze such cyclization has arisen through convergent evolution.

**Figure 1:**
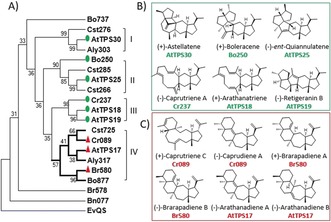
Plant sesterterpene synthases and structures of their products. A) Phylogenetic tree of plant candidate STSs (see the Supporting Information for details). A fungal STS (EvQS) is included as an outgroup. Green circles are previously characterized plant STSs; red triangles are STSs characterized in this work. B) Structures of products generated by previously characterized STSs. C) Structures of sesterterpenes with novel scaffolds produced by the three STSs investigated in this work.

In contrast to the convergent biosynthesis of similar sesterterpene structures via the unified cyclization path by fungal and plant STSs, new STS functionalities could also have emerged through divergent evolution within the same lineage. We previously identified 19 full length candidate STS genes, of which six, which spanned three main phylogenetic clades (clade I, II, III), were functionally characterized and their dominant products shown to be “fungal‐type” sesterterpenes (Figure 1 B).^3^ In this study, we focus on the functional characterization of three new TPSs (Cr089 from *Capsella rubella*, AtTPS17 from *Arabidopsis thaliana* and Br580 from *Brassica rapa*), belonging to an unexplored clade (clade IV) that has diverged from the three other plant STS clades (Figure 1). We used *Nicotiana benthamiana* as the heterologous host for transient expression of these TPS genes, a system that had previously proved very successful for expression of plant STSs.^3^, ^8^

Transient expression of individual STS with a GFPPS gene by “agro‐infiltration”,8a a process where agrobacteria (*Agrobacterium tumefaciens*) carrying the expression constructs were infiltrated into the undersides of leaves of *N. benthamiana*, resulted in the production of multiple peaks. These peaks, including (**2**–**8**), all have a characteristic *m*/*z=*340 [M]^+^ fragment, suggesting the formation of sesterterpenes (MW=340 for C_25_H_40_; Figure 2 A; Supporting Information, Figure S3). Large‐scale co‐expression of individual Clade IV STS genes with a GFPPS gene in *N. benthamiana* leaves using a vacuum infiltration system that we have developed allowed for production and isolation of compounds **2**–**8** in milligram quantities for structural elucidation by comprehensive NMR analysis.^9^ Other peaks were either low abundance or inseparable by chromatography. Compounds **2 a** and **2 b** were present across all the GC‐MS chromatograms of extracts of leaves expressing three different TPSs, although **2 b** is a very minor product in those of Br580 and AtTPS17 (Figure 2 A). Structural elucidation established **2 a** and **2 b** as a pair of C_18_ epimers having a 15/5 fused bicyclic ring system. Close examination of the NOESY spectra of **2 a** and **2 b** suggest their relative stereochemistry as (14S*,15R*,18S*)‐**2 a** and (14S*,15R*,18R*)‐**2 b**, respectively (Supporting Information, Tables S3,S4). In the cyclization paths catalyzed by the Clade I, II, and III STSs, the C_19_ carbocation (**A**) undergoes a 1,5 hydride shift to form the unified C_12_ bicyclic carbocation (**A1**) that leads to formation of one group of dominant end products (Figure 2 B). In contrast, Clade IV STSs seem to quench the cyclization path at an early stage by deprotonation of the C_19_ carbocation to form **2 a** and **2 b**, which then undergo reprotonation leading to the formation of multiple products. Compounds **2 a** and **2 b** were named (+)‐brassitetraene A and B, respectively.

**Figure 2:**
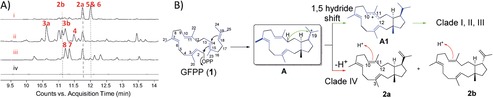
Metabolite profiles of the products of the four STSs and their biogenetic routes. A) GC‐MS traces of EtOAc extracts of *N. benthamiana* leaves expressing different constructs. i) AtGFPPS1+Br580; ii) AtGFPPS1+Cr089; iii) AtGFPPS1+AtTPS17; iv) green florescent protein as control. B) Functional divergence of plant STSs leads to rerouting of the cyclization path from cation A.

Compounds **3 a** and **3 b** were inseparable by silica chromatography, and attempts to separate them on an alumina column resulted in recovery of **3 b** only, suggesting that **3 a** might be isomerized to **3 b** during chromatography. Compound **3 b** was isolated as a white solid and a crystal structure obtained. NMR and X‐ray diffraction analysis of **3 b** (Supporting Information, Table S7) established its scaffold as an unprecedented 6/11/5 tricyclic fused ring, with relative stereochemistry (6S*, 11S*, 14S*, 15R*, 19R*)‐**3 b** (Figure 3). The absolute configuration of **3 b** could not be elucidated based on the X‐ray diffraction data due to the very weak anomalous scattering signal in this carbon/hydrogen only compound. Compound **3 a** was isolated from a mixture containing **3 a** and **3 b** using silver nitrate impregnated silica chromatography and its structure determined by NMR as an isomer of **3 b** differing in one double bond position (C_7_=C_21_ of **3 a** Vs C_7_=C_8_ of **3 b**) (Figure 3Supporting Information, Table S6). Compound **3 a** and **3 b** were named (−)‐caprutriene B and (+)‐caprutriene C, respectively.

**Figure 3:**
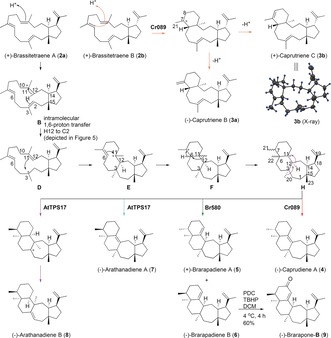
Proposed cyclization mechanism leading to the formation of compounds **3**–**8** and synthesis of (−)‐brarapone B (**9**). The crystal structure of **3 b** is shown with ellipsoids set at 50 % probability.^16^

Compounds **4**–**8** were all tetracyclic sesterterpenes having two C=C bonds, evident by the four olefinic signals in their ^13^C NMR spectra. Further analysis of their 2D NMR spectra, including HSQC and HMBC, established the nature of the novel 6/6/7/5 fused ring system shared by these five compounds, with variation of the position of one *endo* C=C bond (Supporting Information, Tables S7–S11 and Figures S32–S65). NOESY spectra revealed differences in stereochemistry mainly at the C_6_ and C_7_ positions, with **4**, **5**, **6**, **7** and **8** having relative stereochemistry as (3S*, 6S*, 7R*, 14S*, 15R*, 18S*)‐**4**, (3S*, 6S*, 7R*, 12S*, 14S*, 15R*, 18S*)‐**5**, (3S*, 6S*, 7S*, 12S*, 14S*, 15R*, 18S*)‐**6**, (3S*, 6S*, 7S*, 14S*, 15R*, 18S*)‐**7** and (6S*, 7S*, 11R*, 12R*, 14S*, 15R*, 18S*)‐**8**, respectively. Compounds **4**–**8** were named (−)‐caprudiene A, (+)‐brarapadiene A, (−)‐brarapadiene B, (−)‐arathanadiene A, and (−)‐arathanadiene B, respectively.

Having determined the relative stereochemistry of structures **2**–**8**, we then sought to establish the absolute configurations of these compounds by constructing a conjugated system in the molecule to allow for measurement of ECD spectra in the near UV to visible wavelength range, followed by comparison with ECD spectra predicted by quantum chemical calculations. We successfully installed an enone moiety into compound **6** to form compound **9** via metal‐mediated allylic oxidation using pyridinium dichromate coupled with *tert*‐butyl hydroperoxide (Figure 3). Comparing the experimental ECD spectrum of (−)‐**9** with the calculated one of (−)‐**9** established its absolute structure as (3*S*, 6*S*, 7*S*, 12*S*, 14*S*, 15*R*, 18*S*)‐**9** (Supporting Information, Figure S1). The absolute structure of its precursor compound **6** is assigned as (3*S*, 6*S*, 7*S*, 12*S*, 14*S*, 15*R*, 18*S*)‐**6**. Although the absolute configurations of other compounds are yet to be established, the fact that compounds **2**–**5** and **7**–**8** share the same relative stereochemistry with **9** at C_14_ and C_15_ as well as the communal precursors **2 a** and **2 b** strongly suggests that their absolute structures are as depicted in Figure 3.

Interestingly, the isolated compounds **2**–**8** include bi‐, tri‐, and tetracyclic scaffolds that are derived from the corresponding carbocation intermediates during cyclization, and reflect a unified cyclization path towards the formation of the 6/6/7/5 scaffold catalyzed by the three TPSs investigated herein. Based on the structures, we propose a cyclization path that involves a second‐stage reprotonation at C_10_=C_11_ of the common substrate **2 a**/**b** to generate a tertiary C_11_ carbocation **B**, which may then undergo either direct C_6_–C_11_ ring closure followed by deprotonation to form tricyclic caprutriene B and C, or (H_12_‐C_2_) intramolecular 1,6‐proton transfer (**B**→**D**) resulting in net double bond migration (C_10_=C_11_ in **2 a** to C_11_=C_12_ in **D**). Subsequent ring closure events (C_3_–C_12_, C_6_–C_11_) followed by 1,2‐hydride and 1,2 methyl shifts and deprotonation then yield compounds **4**–**8**, each with a 6/6/7/5 fused ring system.

Similar deprotonation/reprotonation sequences have been reported for other Class I TPS‐catalyzed cyclizations, including sesquiterpene synthases (for example, for tobacco 5‐epi‐aristolochene synthase (TEAS), and more recently for fungal STS astellifadiene synthase).2c, 5a, 10 To support our proposed cyclization mechanism, we also conducted in vivo feeding and time course experiments whereby we harvested *N. benthamiana* leaves expressing the plant STS genes at different time points (4 d, 5 d, 8 d, and 10 d) after watering the plant with pure D_2_O for seven days, starting from the third day post‐infiltration,^11^ and analyzed the leaf extracts by GC‐MS. Our results showed that the TIC area ratio of the intermediates **2 a** versus products **5**–**8** or **2 b** versus **3 a** and **3 b** decreased significantly over time. In sharp contrast, the abundance ratio of the MS fragment 341/340 increased correspondingly for Br580, AtTPS17 and Cr089 (Supporting Information, Figure S2). This suggests that intermediate **2 a**/**2 b**, once formed, might either reside in or released and rebound to the active site for reprotonation by D_2_O. Since this new unified cyclization path is diverted from cyclization paths catalyzed by plant STSs from Clades I, II, and III, finding conserved amino acid sites in clade IV STS that are different from other clades could potentially unveil the key amino acids that reroute the cyclization path from cation **A**. Sequence comparison of the characterized plant STSs with the Clade IV STSs identified two conserved amino acid sites (N354 and K424 in AtTPS17) in the active site regions of Clade IV STSs that differed from those of other STSs (Figure 4 A; Supporting Information, Figure S4). Mutation of these two amino acid sites in AtTPS17, Br580 and Cr089 to the amino acids present in STSs from Clade I–III, that is from asparagine to aspartic acid or from lysine to isoleucine or serine, resulted in loss of enzymatic activity in most cases, possibly due to protein folding problems. However, mutant AtTPS17^K424I^ was still active (ca. 68 % activity compared to wild‐type AtTPS17, as measured by total amounts of products **2 a**, **7** and **8** produced), but its product profile was altered; it showed a significant reduction in the formation of tetracyclic compounds **7** and **8**, as evident by the decreased area ratio of **7** and **8** versus **2 a** in the mutant (1.3) compared to that in the wild‐type (2.5; Figure 4).

**Figure 4:**
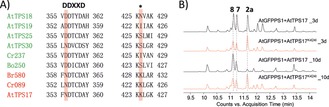
Sequence comparison of all characterized plant STSs and mutagenesis of key residues. A) Alignment of STS amino acid sequences in the active site region identified two conserved residues in Clade IV STSs (in red); B) GC‐MS chromatograms (representative of at least three biological replicates) of extracts of *N. benthamiana* leaves expressing wild‐type AtTPS17 and mutants AtTPS17^K424I^ (in red) harvested on the third and tenth day, respectively.

Moreover, prolonged reaction time (10 days) did not lead to significant further conversion of **2 a** to **7** and **8** by mutant AtTPS17^K424I^, which is in stark contrast to the complete consumption of **2 a** by the wild‐type AtTPS17 after 10 days post infiltration, further suggesting that this site might be involved in the reprotonation process and that lysine424 might be serving as a general acid/base aiding in reprotonation.^12^

Quantum chemical computations (mPW1PW91/6–31+G(d,p)//B3LYP/6–31+G(d,p); see Supporting Information for details) were carried out to assess the viability of our proposed mechanism. An intramolecular 1,6‐proton transfer event for the generation of carbocation **D** was shown to be energetically viable. (Figure 5). Although cation **D** might also be formed via deprotonation of C_11_ to generate a C_11_=C_12_ double bond followed by a second reprotonation of C_2_=C_3_, the incorporation of just one deuterium (*m*/*z* 341 rather than 342) in the mass spectra of products in our D_2_O feeding experiments exclude this possibility (Supporting Information, Figure S2). There are precedents for intramolecular proton transfer in terpene forming reactions, but not all such proposed transformations are energetically viable.^13^ Further cyclization involves a conformational change of carbocation **D** to prepare for the subsequent ring closure event. Cation **E** is found not to be a minimum (at least on the gas phase potential energy surface); instead a concerted bicyclization, in which the C−C bond formation events occur asynchronously, is found for the conversion of cation **D** to **F**.^14^ The overall barrier from cation **B** to the transition state structure for the **D**→**F** conversion is about 18 kcal mol^−1^ (for analogous reactions of other conformers, see the Supporting Information, Figures S73, S74). The **D**→**F** reaction sets the relative stereochemistry of C_3_, C_6_, C_11_ and C_12_. Cation **F**, once generated, can undergo low barrier 1,2 hydride and methyl shifts to form cation **G**, which, upon deprotonation, leads to compounds **4**, **5**, **6**, and **7**. Further 1,2 hydride and methyl shifts of cation **H** followed by deprotonation furnish (−)‐arathanadiene B (**8**).

**Figure 5:**
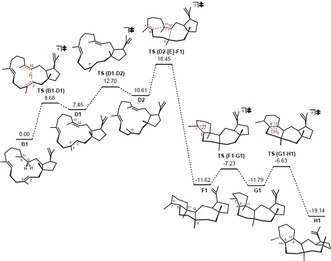
Structures involved in the conversion of carbocation **B** (here, conformer **B1**) to carbocation **H**; relative energies from mPW1PW91/6–31+G(d,p)//B3LYP/6–31+G(d,p) are shown.

To summarize, we have discovered and functionally characterized a phylogenetically diverged clade of plant STSs that catalyze the formation of unprecedented 6/11/5 tricyclic caprutriene B (**3 a**) and C (**3 b**) and 6/6/7/5 tetracyclic (−)‐caprudiene (**4**), brarapadiene A (**5**) and B (**6**) and arathanadiene A (**7**) and B (**8**) via a new unified cyclization path. Sequence analysis and site‐directed mutagenesis further identified a key amino acid site (Lys424 in AtTPS17) implicated in mediating the reprotonation of compounds **2 a**/**b**. Although we have not yet determined the exact mechanism by which Lys424 may participate and mediate the reprotonation reaction due to the lack of an accurate plant STS crystal structure as a template for modeling (Supporting Information, Figure S4B),^15^ we hypothesize that Lys424 may aid in holding H_2_O in position as a proton donor. Having the crystal structure of a STS in complex with relative substrates will be key to understanding the mechanisms. Our findings pave the way for further mechanistic studies and for protein engineering experiments aimed at understanding and generating sesterterpene diversity. Moreover, together with quantum chemical calculations, we demonstrate that a carbocation cyclization/rearrangement path that involves intramolecular 1,6 proton transfer and concerted but asynchronous double cyclization is energetically viable. This diverted cyclization path underscores the phylogenetic divergence of the plant STSs and the tremendous chemical engineering potential harbored within the plant kingdom. Our work also demonstrates the power of evolutionary genomics‐based approaches for unlocking this potential for natural product discovery.

## Conflict of interest

The authors declare no conflict of interest.

## Supporting information

As a service to our authors and readers, this journal provides supporting information supplied by the authors. Such materials are peer reviewed and may be re‐organized for online delivery, but are not copy‐edited or typeset. Technical support issues arising from supporting information (other than missing files) should be addressed to the authors.

Supplementary
