# Supplementary material for: Diverged Plant Terpene Synthases Reroute the Carbocation Cyclization Path towards the Formation of Unprecedented 6/11/5 and 6/6/7/5 Sesterterpene Scaffolds
Source: Angew Chem Int Ed Engl. 2017 Dec 28;57(5):1291–5. doi: 10.1002/anie.201711444 (PMC5814883; doi:10.1002/anie.201711444)
Supplement: Supplementary file 1 — Supplementary [file ANIE-57-1291-s001.pdf]

Angewandte Chemie
Eine Zeitschrift der Gesellschaft Deutscher Chemiker

Supporting Information

Diverged Plant Terpene Synthases Reroute the Carbocation Cyclization Path towards the Formation of Unprecedented 6/11/5 and 6/6/7/5 Sesterterpene Scaffolds

*Ancheng C. Huang, Young J. Hong, Andrew D. Bond, Dean J. Tantillo, and Anne Osbourn**

anie_201711444_sm_miscellaneous_information.pdf

Table of Contents

SI Materials and Methods……………………………………………………………………………………..…………………………………P3-6

Tables S1-12. …………………………………………………………………………………………………......…….……………………..P7-17

Figures S1-74. ....……………………..………………….........…………P18-54

SI Materials and Methods

Phylogenetic analysis

Amino acid sequences of candidate plant STSs encoded by genes that were previously identified to be co-localized with a PT gene were aligned using clusterW, together with a fungal STS (EvQS) as outgroup and an STS homologue (Bn077) encoded by a TPS gene (LOC106387077) that is not clustered with a PT gene.[1] The maximum-likelihood phylogenetic tree was generated based on this alignment using Mega5 with 1000 bootstrap resampling. Detailed information on the candidate STSs, their encoding genes and corresponding plant species can be found in Table S1.

Cloning and transient expression.

The coding sequences of *At4g15560*, *Br580* (LOC103859580) and *Cr089* (CARUB_v10016089mg) were retrieved from TAIR and NCBI. These sequences were synthesized by Integrated DNA Technologies to include the 5' (GGGGACAAGTTTGTACAAAAAAGCAGGCT) and 3' (GGGGACCACTTTGTACAAGAAAGCTGGGT) attB sites for Gateway cloning. Synthesized gene fragments were inserted into the pDONR207 (Gen^R) entry vector using standard Gateway cloning with BP clonase (Thermo Fisher Scientific). BP reaction products were transformed into chemically competent DH5α cells by heat shock at 42°C for 45 s and isolated plasmids harboring the desired inserts confirmed by sequencing (Eurofins). Confirmed genes were cloned into the pEAQ-HT (Kan^R) expression vector by standard LR reaction using Gateway LR clonase II (Thermo Fisher Scientific). *AtTPS17* (*At3g14490*) and AtGFPPS1 (At3g14530) were cloned into the pEAQ-HT vector as described in our previous work.[1a] pEAQ-HT expression vectors harboring the desired genes were transformed into *Agrobacterium tumefaciens* strain LBA4404 using a freeze-thaw protocol [2]. Transformed cells were rescued with LB medium (600 μl) and incubated at 28 °C for 4 h before plating on LB agar plates (50 μg/ml rifampicin, 50 μg/ml kanamycin and 100 μg/ml streptomycin) which were incubated at 28°C for 2 days. Positive colonies were inoculated into LB liquid broth and cultured at 28 °C overnight until the OD600 reached ca. 2.0. Cultures were pelleted at 3220 g for 5 min and re-suspended with MMA buffer (1 ml, containing 1 M $MgCl_2$, pH5.6 MES buffer and 1% acetosyringone). *A. tumefaciens* strains harbouring different expression constructs were then mixed accordingly to bring the OD600 for each construct to ca. 0.2 in the final solution for infiltration of *N. benthamiana* leaves. *A. tumefaciens* LBA4404 carrying a green florescent protein expression construct in pEAQ-HT was used as a control. The *A. tumefaciens* suspensions were infiltrated into the undersides of leaves of 4-week old *N. benthamiana* plants using a syringe without a needle in triplicate. The infiltrated plants were maintained in a containment glasshouse (with a 16 h light/8 h dark cycle) for five days before being harvested. Harvested leaves expressing different construct combinations were lyophilized and stored at -80 °C until analysis.

Metabolite extraction and GC-MS analysis.

N. benthamiana leaves expressing genes of interest were harvested 5 days post infiltration and lyophilized unless otherwise specified. Dry leaves (3 mg) were homogenized into powder with tungsten beads (Qiagen) and extracted with EtOAc (250 μl, containing 10 μg/ml nonadecane-d_{40} as internal standard) with sonication for 0.5 h. The suspensions were centrifuged at 16,000 g for 2 min and the supernatants (200 μl) analyzed directly by GC-MS as described below.

GC-MS analysis was performed on an Agilent GC (7890B)-MSD (5977A) equipped with a robotic multi-purpose auto-sampler (MPS) and a Zebra-5HT INFERO capillary column (35mX0.25mmX0.1 μm film thickness, phenomenex). A 1 μl aliquot of each sample was injected in pulsed splitless mode into the GC inlet which was set at 250 °C. The pulse pressure was set at 20 psi for 0.5 min after injection. The inlet was purged after 0.5 min with a split vent flow at 100 ml/min. A gradient temperature GC program was used for qualitative and quantitative analyses of sesterterpenes. The initial temperature was set at 170 °C and held for 2 min, increased to 250 °C at 6 °C/min over 13 min, ramped up to 340 °C at 20 °C/min and held at 340 °C for 1 min. To examine possible sesquiterpenes and diterpenes produced by the selected TPSs, the GC temperature program was adjusted to: initial temperature 100 °C, held for 2 min, increased to 290 °C at 8 °C/min over 25.75 min and then ramped up to 340 °C at 40 °C/min and held for 5 min. The carrier gas was helium. The gas flow rate was constant at 1.0 ml/min. The GC and MS auxiliary interface was set at 280 °C. Metabolites eluted from the GC were ionized by electron impact (EI, 70 eV) ionisation with collision energy set at 70 eV. The temperatures of the MS quadrupler and source were set at 230 °C and 150 °C, respectively. MS signals were acquired in full scan mode with the scan range set from 60 to 800 Da.

Isolation of sesterterpenes following large-scale vacuum-infiltration of *N. benthamiana* leaves.

To direct the metabolic flux towards sesterterpene production, we also co-infiltrated an *A. tumefaciens* strain harboring an expression construct for 1-deoxy-D-xylulose 5-phosphate synthase (DXS) (*At4g15560*),[3] an enzyme from the non-mevalonate pathway, with AtGFPPS1 and STS genes in our scale-up experiments. Seed cultures of *A. tumefaciens* strains harboring expression constructs for the relevant AtGFPPS1 and STSs were prepared as described above and 100 ml inoculated into 1 l LB broth containing 50 μg/ml rifampicin, 50 μg/ml kanamycin and 100 μg/ml streptomycin. Cultures were incubated in a shaker (28 ºC and 220 rpm) for about 15 h until the OD600 reached ca. 2.0. *A. tumefaciens* cells were pelleted by centrifuging at 6500 g for 25 min. Supernatants were discarded. The pellets were then resuspended in MMA buffer (200 ml) and kept in the dark for 1 h before performing vacuum infiltration. Batches of four *N. benthamiana* plants were simultaneously infiltrated using a custom-built vacuum chamber with an oil pump, as described in the literature [4]. Briefly, the *A. tumefaciens* suspensions containing the different expression constructs were mixed and topped up to ca. 10 l with MMA buffer in a 10 l basin. Plants were secured in a rack and inverted to submerge the leaves into the bacterial suspension in the basin. The basin was placed into a vacuum chamber and the plants vacuum-infiltrated by imposing a vacuum to ca. 0.1 bar followed by venting. The plants were then maintained in a containment growth room (16h light, 8h dark) for five days before the leaves were harvested and lyophilized. Dried leaf material was ground to a powder using a mortar and pestle and extracted with hexanes at room temperature with agitation for three days. Extracts were filtered each day and the leaf material re extracted with fresh solvent. Extracts were then combined, dried *in vacuo* and purified by silica column chromatography (SCC) using an Isolera One (Biotage) automatic flash purification system.

*Isolation of (+)-brassitetraene A (**2a**), (-)-brarapadiene A (**5**) and B (**6**)*. *N. benthamiana* plants (155) were co-infiltrated with *A. tumefaciens* strains containing *DXS, GFPPS* (*At3g14530*) and *Br580* expression constructs. Five days later, infiltrated leaves were harvested and lyophilezed. 90 g of the dried leaf material was extracted with hexanes (1.5 l per day) for three days. Combined extracts were dried *in vacuo* and dried extracts subjected to SCC using 100% hexanes as an eluent, yielding (+)-brassitetraene A (**2a**, 87.8 mg) and a mixture of (-)-brarapadiene A and B (93.6 mg). (-)-Brarapadiene A (**5**, 3.0 mg) and (-)-brarapadiene B (**6**, 3.6 mg) were isolated by repeated silver nitrate impregnated silica (from SiliCycle Inc.) column chromatography using 100% hexanes to 1% diethyl ether in hexanes as eluents.

2a: colorless liquid; $[\alpha]_D^{20}$ = +71.1 (*c* 0.96, acetone); $[M]^+$ = 340 (EI-MS), calcd. for $C_{25}H_{40}$= 340; ^{1}H and ^{13}C NMR data, see Table S3.

5: colorless liquid; $[\alpha]_D^{20}$= +26.3 (*c* 0.16, acetone); $[M]^+$ = 340 (EI-MS), calcd. for $C_{25}H_{40}$= 340; ^{1}H and ^{13}C NMR data, see Table S8.

6: colorless liquid; $[\alpha]_D^{20}$= -8.5 (*c* 0.39, acetone); $[M]^+$ = 340 (EI-MS), calcd. for $C_{25}H_{40}$= 340; ^{1}H and ^{13}C NMR data, see Table S9.

*Isolation of (+)-brassitetraene B (**2b**), (-)-caprutriene B (**3a**), (+)-caprutriene C (**3b**) and (-)-caprudiene A (**4**)*. *N. benthamiana* plants (223) were co-infiltrated with *A. tumefaciens* strains containing *DXS, AtGFPPS1* (*At3g14530*) and *Cr089* expression constructs. Dried leaves (130 g) were extracted with hexanes (2 l per day) for three days. Combined extracts were purified by SCC using 100% hexanes as an eluent, yielding pure (-)-caprudiene A (4.5 mg) as a colorless liquid and three other main fractions containing caprutriene B and C and brassitetratene A and B, respectively. Fraction 1 containing ~87% compound **3a** and **3b** (12.0 mg, **3a**: **3b** approximates 3:4 based on the corresponding peak areas in the GC-MS chromatogram) was first purified by SCC without any separation. Subsequent purification of a small portion of this fraction on neutral allumia column resulted in the recovery of only compound **3b** (7.8 mg) as a white solid, suggesting that compound **3a** was completely converted to **3b** on allumina column based on the original amount of the mixture and the compounds isolated. Fraction 2 containing ~85% **3a** and **3b** in a ratio of ~4:3 (13.3 mg) was loaded on a silver nitrate impregnated silica column to achieve separation of **3a** and **3b** with 5.2 mg of **3a** purified. Fraction 3 (17.2 mg) containing a mixture of compound **2a** and **2b** in a ratio of ~1:3. Attempts to separate these two compounds using silica and alumina column chromatography were not successful. However, we managed to separate the mixture of compounds **2a** and **2b** using silver nitrate impregnated silica to yield pure (+)-brassitetraene A (**2a**, 3.4 mg) and (+)-brassitetraene B (**2b**, 6.8 mg).

2b: colorless liquid; $[\alpha]_D^{20}$= +3.8 (*c* 0.13, acetone); $[M]^+$ = 340 (EI-MS), calcd. for $C_{25}H_{40}$= 340; ^{1}H and ^{13}C NMR data, see Table S4.

3a: colorless liquid; $[\alpha]_D^{20}$= -24.3 (*c* 0.28, acetone); $[M]^+$ = 340 (EI-MS), calcd. for $C_{25}H_{40}$= 340; ^{1}H and ^{13}C NMR data, see Table S5.

3b: white solid; $[\alpha]_D^{20}$= +21.2 (*c* 0.33, acetone); $[M]^+$ = 340 (EI-MS), calcd. for $C_{25}H_{40}$= 340; ^{1}H and ^{13}C NMR data, see Table S6.

4: colorless liquid; $[\alpha]_D^{20}$= -124.3 (*c* 0.23, acetone); $[M]^+$ = 340 (EI-MS), calcd. for $C_{25}H_{40}$= 340; ^{1}H and ^{13}C NMR data, see Table S7.

*Isolation of (-)-arathanadiene A (**7**) and (-)-arathanadiene B (**8**). N. benthamiana* plants (282) were co-infiltrated with *A. tumefaciens* strains containing *DXS, AtGFPPS1* (*At3g14530*) and *AtTPS17* (*At3g14490*) expression constructs. Dried leaves (142 g) were extracted with hexanes (2 l per day) for three days. Combined extracts were purified by SCC using 100% hexanes as the eluent to give two fractions containing compounds **7** and **8**. Fraction1 (87 mg) containing compound **7** was further purified by SCC and normal phase (Luna 5 μm silica column, 100 Å, 250x10 mm, Phenomenex) semi-preparative HPLC using 100% hexanes as mobile phase for elution to give (-)-arathanadiene A (**7**, 14.6 mg) as a colorless liquid. Fraction2 (147 mg) containing compound **8** was further purified by repeated SCC to yield (-)-arathanadiene B (**8**, 11.6 mg) as a colorless liquid.

7: colorless liquid; $[\alpha]_D^{20}$= -76.9 (*c* 0.36, acetone); $[M]^+$ = 340 (EI-MS), calcd. for $C_{25}H_{40}$= 340; ^1H and ^{13}C NMR data, see Table S10.

8: colorless liquid; $[\alpha]_D^{20}$= -20.1 (*c* 0.79, acetone); $[M]^+$ = 340 (EI-MS), calcd. for $C_{25}H_{40}$= 340; ^1H and ^{13}C NMR data, see Table S11.

Synthesis of (-)-brarapone B for ECD measurement.

To a suspension of pridinium dichomate (PDC, 62 mg, 17 *m*mol, 1.1 equiv) in DCM (1 mL) at 4 °C was added slowly *tert*-butyl-hydroperoxide (TBHP, 5-6 M in decane, 100 mL, 30 equiv). After vortexing for 0.5 h, to the suspension was added a solution of mixture of compounds **5** and **6** (**5**:**6**≈1:2, 53 mg, 15 *m*mol, 1 equiv) in 0.2 mL DCM. The resulting reaction mixture was vortexed at 4 °C for 4 h. To quench the reaction, hexanes (1 mL) was added to the reaction mixture. The suspension was filtered through a silica plug which was further rinsed with 10% ethyl acetate (in hexanes, 3 mL) three times. Combined filtrates were dried under N_2 and dry reaction products purified by SCC to furnish (-)-brarapone B (**9**, 22.3 mg) in ~60% yield as a colorless liquid.

9: colorless liquid; $[\alpha]_D^{20}$= -22.0 (*c* 0.6, acetone); $[M]^+$ = 340 (EI-MS), calcd for $C_{25}H_{40}$= 340; ^1H and ^{13}C NMR data, see Table S12.

Structural characterization of sesterterpenes by NMR and other spectroscopic techniques.

Standard 1D and 2D NMR spectra including ^1H, ^{13}C, DEPT135, COSY, HSQC, HMBC and NOESY were acquired on a Bruker 400 MHz Topspin NMR spectrometer. All signals were acquired at 298 K. Samples were dissolved in $CDCl_3$ or C_6D_6 for data acquisition and calibrated by referencing to either residual solvent ^1H and ^{13}C signal or TMS. Detailed structural assignments and tabulated NMR data for compounds **2**-**9** were presented in the **Tables S3-12**. Optical rotations of compounds **2**-**9** were measured in acetone using a PerkinElmer Polarimeter (Model 341) with a 100 mm path cell (1 ml) at 20 °C and converted to specific optical rotation using equation $[\alpha]_D^{20}$= 100 x (measured optical rotation)/concentration (g/100ml). ECD measurement was performed on a Chirascan-plus circular dichroism spectrometer (Applied Photophysics Ltd). Compound **9** was dissolved in absolute ethanol at a concentration of 0.5 mg/ml for ECD measurement. The scan range was set at 200-500 nm and the measurement was done in triplicate at room temperature (~17 °C).

Single crystal X-ray diffraction analysis.

Single crystal X-ray analysis was carried out for compound **3b** on a Bruker D8-QUEST instrument equipped with a PHOTON-100 area detector and Incoatec IμS Cu microsource (wavelength = 1.5418 Å, beam diameter at the crystal ca 100 μm). Crystals were mounted on an X-ray transparent loop (Mitegen) using an inert oil and cooled to 180(2) K using an open-flow N_2 cryostat. Data were collected and processed using the *APEX3* software package (Bruker). Structures were solved and refined using *SHELXT* and *SHELXL* (Bruker). In the absence of any significant anomalous scatterers, reliable indications of the absolute structure could not be obtained. The crystallographic data have been deposited at the Cambridge Crystallographic Data Centre: CCDC1583364

Time course and feeding experiments

Three four week old *N. benthamiana* plants were each infiltrated with combinations of *A.tumefacien* strains carrying different expression constructs: *AtGFPPS1+Cr089*, *AtGFPPS1+Br580*, and *AtGFPPS1+AtTPS17*, respectively, using a needless syringe as outlined above. Five leaves of each plant were infiltrated. The infiltrated plants were maintained in a growth chamber with 16 h light/ 8 h dark cycle at 22 °C. One leaf from each plant was harvested on the thrid day, before watering the plants with D_2O (~5 ml/day) until the tenth day. Infiltrated leaves expressing different combinations of genes were harvested on the fourth, fifth, eight and tenth days, respectively. Harvested leaves were lyophilized and analyzed by GC-MS as described above.

Sequence comparisons and homology modelling.

Amino acid sequences of protein sequences listed in TableS1 were aligned using Uniprot (http://www.uniprot.org/). Homology models of *AtTPS17* were generated on modelling server Phyre[2] (http://www.sbg.bio.ic.ac.uk/phyre2/html/page.cgi?id=index) using the crystal structure of 5-epi-aristolochene synthase as template.[5] Docking of STSs substrate GFPP was performed using Autodock4.0 and AutoDockTool (ADT) developed by The Scripps Research Institute.[6]

Mutagenesis of *AtTPSs17, Br580, and Cr089*.

Site-directed mutagenesis was performed by PCR amplification using the entry vector pDONR207 harbouring the wild type genes as templates and the mutated complementary sequences as primers (listed in Table S2). DMSO (5%) was added to the PCR reaction to alleviate formation of primer dimers. PCR-amplified products were purified with a PCR quick purification kit and eluted with 15 µl elution buffer. The purified products were digested with DpnI (New England Lab) at 37 ºC with agitation at 500 rpm for 2 h to remove the original methylated plasmids before transformation into competent cell DH5α by heat shock at 42 ºC for 45 s. The transformed cells were rescued with LB medium (0.3 ml) at 37 ºC for 2 h and plated on gentamycin (20 µg/ml) containing LB agar plates which were incubated at 37 ºC for 16 h. Positive colonies were inoculated into LB-gentamycin broth (10 ml) and cultured at 37 ºC for 10 h. Plasmids were extracted from the cultures and mutations were confirmed by sequencing (Eurofins). Mutagenized genes were gateway cloned into the pEAQ-HT expression vector, transformed into LBA4404 and co-expressed with LBA4404/GFPPS1 in our transient tobacco expression system as described in the Cloning and transient expression section.

Quantum chemical calculations. All quantum calculations were performed with the *GAUSSIAN09* software suite.[7] Geometries were optimized using the B3LYP density functional theory (DFT) method and the 6-31+G(d,p) basis set.[8] All stationary points were characterized as minima or transition state structures using frequency calculations. All reported energies include zero-point energy corrections (unscaled) from these frequency calculations. mPW1PW91 single point energies were calculated for all structures [9]. These methods are well established for examining carbocation rearrangement reactions.[10] Structures of intermediate carbocations and ransition state structures are presented in Figure S73-74**.**

To compute the ECD spectrum of (-)-brarapone B (**9**) in Figure S1, conformational analysis was carried out using CONFLEX (5kcal/mol window). The resulting 33 conformers were then optimized further using B3LYP/6-31G(d). 5 conformers (3kcal/mol cut off energy) were subjected to full optimization using B3LYP/6-31+G(d,p). 3 contributing conformers (>1% relative abundance) were selected and subjected to the TD-DFT calculations of the UV/ECD by using B3LYP/6-31+G(d,p) in gas phase.

References

[1] a) A. C. Huang, S. A. Kautsar, Y. J. Hong, M. H. Medema, A. D. Bond, D. J. Tantillo, A. Osbourn, *Proc. Natl. Acad. Sci. USA* **2017**, *114*, E6005-E6014; b) M. Okada, Y. Matsuda, T. Mitsuhashi, S. Hoshino, T. Mori, K. Nakagawa, Z. Quan, B. Qin, H. Zhang, F. Hayashi, H. Kawaide, I. Abe, *J. Am. Chem. Soc.* **2016**, *138*, 10011-10018.

[2] H. Chen, R. S. Nelson, J. L. Sherwood, *BioTechniques* **1994**, *16*, 664-668, 670.

[3] a) J. Andersen-Ranberg, K. T. Kongstad, M. T. Nielsen, N. B. Jensen, I. Pateraki, S. S. Bach, B. Hamberger, P. Zerbe, D. Staerk, J. Bohlmann, B. L. Møller, B. Hamberger, *Angew. Chem.* **2016**, *128*, 2182-2186; b) W. N. Hunter, *J. Biol. Chem.* **2007**, *282*, 21573-21577.

[4] J. Reed, M. J. Stephenson, K. Miettinen, B. Brouwer, A. Leveau, P. Brett, R. J. M. Goss, A. Goossens, M. A. O'Connell, A. Osbourn, *Metab. Eng.* **2017**, *42*, 185-193.

[5] L. A. Kelley, S. Mezulis, C. M. Yates, M. N. Wass, M. J. E. Sternberg, *Nat. Protoc.* **2015**, *10*, 845.

[6] G. M. Morris, R. Huey, W. Lindstrom, M. F. Sanner, R. K. Belew, D. S. Goodsell, A. J. Olson, *J. Comput. Chem.* **2009**, *30*, 2785-2791.

[7] M. J. Frisch, G. W. Trucks, H. B. Schlegel, G. E. Scuseria, M. A. Robb, J. R. Cheeseman, G. Scalmani, V. Barone, B. Mennucci, G. A. Petersson, H. Nakatsuji, M. Caricato, X. Li, H. P. Hratchian, A. F. Izmaylov, J. Bloino, G. Zheng, J. L. Sonnenberg, M. Hada, M. Ehara, K. Toyota, R. Fukuda, J. Hasegawa, M. Ishida, T. Nakajima, Y. Honda, O. Kitao, H. Nakai, T. Vreven, Jr, J. E. Peralta, F. Ogliaro, M. Bearpark, J. J. Heyd, E. Brothers, K. N. Kudin, V. N. Staroverov, R. Kobayashi, J. Normand, K. Raghavachari, A. Rendell, J. C. Burant, S. S. Iyengar, J. Tomasi, M. Cossi, N. Rega, J. M. Millam, M. Klene, J. E. Knox, J. B. Cross, V. Bakken, C. Adamo, J. Jaramillo, R. Gomperts, R. E. Stratmann, O. Yazyev, A. J. Austin, R. Cammi, C. Pomelli, J. W. Ochterski, R. L. Martin, K. Morokuma, V. G. Zakrzewski, G. A. Voth, P. Salvador, J. J. Dannenberg, S. Dapprich, A. D. Daniels, Farkas, J. B. Foresman, J. V. Ortiz, J. Cioslowski, D. J. Fox, *Gaussian 09 Revision D.01*, Gaussian Inc. Wallingford CT 2013, **2013**.

[8] A. D. Becke, *The Journal of Chemical Physics* **1993**, *98*, 5648-5652.

[9] S. P. T. Matsuda, W. K. Wilson, Q. Xiong, *Org. Biomol. Chem.* **2006**, *4*, 530-543.

[10] D. J. Tantillo, *Nat. Prod. Rep.* **2011**, *28*, 1035-1053.

Table S1. Plant STSs used for the phylogenetic analysis, their corresponding gene ids, species names and source of coding sequences

STSs	Gene ID	Species	Source
Aly303 [c]	LOC9304603	*Arabidopsis lyrata*	NCBI
Aly317 [c]	LOC9321114	*Arabidopsis lyrata*	NCBI
AtTPS17 [b]	At3g14490	*Arabidopsis thaliana*	TAIR
AtTPS18[a]	At3g14520	*Arabidopsis thaliana*	TAIR
AtTPS19 [a]	At3g14540	*Arabidopsis thaliana*	TAIR
AtTPS25 [a]	At3g29410	*Arabidopsis thaliana*	TAIR
AtTPS30 [a]	At3g32030	*Arabidopsis thaliana*	TAIR
Bo737 [c]	LOC106333737	*Brassica oleracea*	NCBI
Bo877 [c]	LOC106333877	*Brassica oleracea*	NCBI
Bo250 [a]	LOC106343250	*Brassica oleracea*	NCBI
Br578 [c]	LOC103859578	*Brassica rapa*	NCBI
Br580 [b]	LOC103859580	*Brassica rapa*	NCBI
Cst276 [c]	LOC104779276	*Camelina sativa*	NCBI
Cst725 [c]	LOC104745725	*Camelina sativa*	NCBI
Cst266 [c]	LOC104765266	*Camelina sativa*	NCBI
Cr089 [b]	CARUB_v10016089mg	*Capsella rubella*	NCBI
Cr237 [a]	CARUB_v10016237mg	*Capsella rubella*	NCBI
Bn077 [c]	LOC106387077	*Brassica napus*	NCBI

[a]: STSs characterized in reference [1]; [b]: STSs characterized in this work; [c]: Other TPSs not yet characterized.

Table S2 | Primer sequences used for mutagenesis

Gene ID	Forward primer	Reverse primer
Br580^{N359D}	GCGGTGGCGACTGTTTTTgATGATACATGTGATGCATATG	CATATGCATCACATGTATCATcAAAAACAGTCGCCACCGC
TPS17^{N354D}	CCATGATTGTGACTGTTTTTgATGATACATGTGATGC	GCATCACATGTATCATcAAAAACAGTCACAATCATGG
Cr089^{N353D}	GTGATTGCGACTGTTTTGgATGACACATGTGATGCATATG	CATATGCATCACATGTGTCATcCAAAACAGTCGCAATCAC
Br580^{K429S}	GTTGTAGATGAGATGAAGAgcTTGGCGAGAGCGTACTTGGC	GCCAAGTACGCTCTCGCCAAgcTCTTCATCTCATCTACAAC
Br580^{K429I}	GTTGTAGATGAGATGAAGAtcTTGGCGAGAGCGTACTTGGC	GCCAAGTACGCTCTCGCCAAgaTCTTCATCTCATCTACAAC
TPS17^{K424S}	GTAGCGGTAGATGAGATCAAGAgcTTGGGGAAAGCATACTTGG	CCAAGTATGCTTTCCCCAAgcTCTTGATCTCATCTACCGCTAC
TPS17^{K424I}	GTAGCGGTAGATGAGATCAAGAtcTTGGGGAAAGCATACTTGG	CCAAGTATGCTTTCCCCAAgaTCTTGATCTCATCTACCGCTAC
Cr089^{K423S}	GCAGTAGATGAGATGAAGAgcTTGGGGAAAGCATACTTG	CAAGTATGCTTTCCCCAAgcTCTTCATCTCATCTACTGC
Cr089^{K423I}	GCAGTAGATGAGATGAAGAtcTTGGGGAAAGCATACTTG	CAAGTATGCTTTCCCCAAgaTCTTCATCTCATCTACTGC

Table S3 | ^{1}H (400 MHz), ^{13}C (100 MHz), COSY, HMBC and NOESY NMR (in benzene-d_6) data of (+)-brassitetraene A (**2a**)

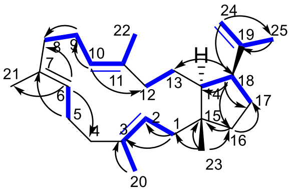

(+)-Brassitetraene A (**2a**)

key COSY

key HMBC

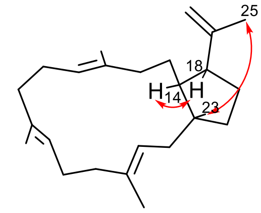

(+)-Brassitetraene A (**2a**)

Key NOESY

Position	δH (ppm) (multiplicity, *J* in Hz, number of H)	δC (ppm)	COSY	HMBC	NOESY
1	2.46 (dd, 14.4, 9.6, 1H);<br>1.77 (m, 1H)	41.57		C3, C4, C14, C15, C16, C23;<br>C2, C3, C14, C15	
2	5.26 (m, 1H)	122.75	H1ab, H20		
3		134.65			
4	2.12 (m, 1H);<br>2.04 (m, 1H)	39.51	H5	C6	
5	2.22 (m, 1H);<br>2.07 (m, 1H)	40.33	H21	C3, C4, C6	
6	5.23 (m, 1H)	124.98	H5ab,<br>H8ab	C4, C8, C21	
7		133.37			
8	2.21 (m, 2H)	25.04	H9	C7, C10	
9	2.10 (m, 2H)	24.79	H8	C8, C22	
10	5.31 (m, 1H);	126.14	H9, H22	C9, C12, C22	
11		133.96			
12	2.02 (m, 2H)	39.08	H13ab	C9, C10, C11, C13, C18	
13	1.23 (m, 1H);<br>1.14 (ddd, 9.2, 6.4,2.8, 1H)	21.72	H12ab	C11, C12, C14, C15, C18, C23;<br>C11	
14	1.87 (br dd, 9.6, 7.2, 1H)	42.09	H18	C1, C12, C13, C17, C18	H18
15		45.75			
16	1.15 (dd, 11.2, 4.0, 1H);<br>1.40 (dd, 11.2, 4.0, 1H)	38.74		C1, C14, C15, C17, C23;<br>C14, C15, C17, C18	
17	1.709 (m, 1H);<br>1.64 (dt, 8.0, 4.0, 1H)	25.24	H18	C16, C18	
18	2.69 (dt, 12.4, 7.2, 1H)	50.02	H14, H17	C13, C14, C16, C17, C19, C24	H14
19		147.31			
20	1.57 (s, 3H)	15.83	H2	C2, C4	
21	1.57 (s, 3H)	15.7	H6	C6	
22	1.57 (s, 3H)	15.7	H10	C10, C11	
23	1.10 (s, 3H)	23.77		C1, C14, C16	H25
24	4.968 (m, 1H);<br>4.815 (br s, 1H)	110.15	H25	C18, C19, C25	
25	1.68 (s, 3H)	23.82	H24ab	C18, C19, C24	H23

Table S4 | ^{1}H (400 MHz), ^{13}C (100 MHz), COSY, HMBC and NOESY NMR (in benzene-d_6) data of (+)-brassitetraene B (**2b**)

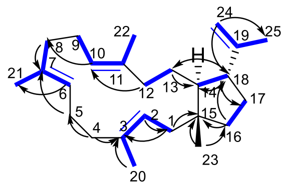

(+)-Brassitetraene B (**2b**)

key COSY
key HMBC

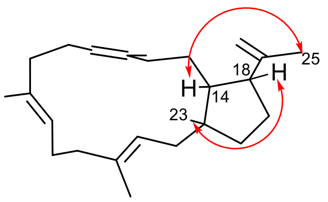

(+)-Brassitetraene B (**2b**)

Key NOESY

Position	δH (ppm) (multiplicity, *J* in Hz, number of H)	δC (ppm)	COSY	HMBC	NOESY
1	1.71 (dd, 20.0, 8.0, 1H); 1.26 (m, 1H)	39.29	H2	C3, C15	
2	5.36 (tq, 8.0, 1.2, 1H)	121.39	H1, H20	C1, C20	
3		135.2			
4	2.14 (m, 2H)	25.05		C3, C5, C6	
5	2.12 (m, 2H);	38.8		C2, C4	
6	5.17 (br t, 6.4, 1H)	125.44	H8, H21	C9, C21	
7		133.69			
8	2.20 (m, 1H)	25.1	H6, H21	C6, C7	
9	2.12 (m, 1H);	39.1	H10	C8, C10, C22	
10	5.20 (m, 1H)	124.98	H9, H22	C8	
11		134.44			
12	2.14 (m, 2H)	37.4	H13a,b	C10, C13	
13	1.60 (m, 1H); 1.39 (m, 1H)	27.44	H12a,b	C12, C14, C18; C11, C12, C14, C18, C22	
14	1.83 (dd, 9.6, 6.4, 1H)	43.65	H18	C12, C13, C15,C16, C17, C18, C19, C23	H25
15		44.73			
16	2.16 (m, 2H)	39.45	H17	C1, C15, C17, C23; C14, C18, C23	
17	1.78 (m, 1H); 1.48 (m, 1H)	29.63	H16	C15, C18, C19	
18	2.343 (ddd, 9.6, 9.2, 7.6, 1H)	54.62	H14	C13, C14, C17, C19, C24, C25	H23
19		149.5			
20	1.56 (s, 3H)	16.86	H2	C2	
21	1.56 (s, 3H)	16.2	H6	C9	
22	1.56 (s, 3H);	16.21	H10	C10, C11, C12	
23	0.90 (s, 3H)	24.16		C15, C16	H18
24	4.89 (d, 2.8, 3H); 4.78 (dq, 2.8, 1.2, 3H)	110.73	H25	C18, C25	
25	1.71 (s, 3H)	18.85	H24	C18, C19, C24	H14

Table S5 | ^{1}H (400 MHz), ^{13}C (100 MHz), COSY, and HMBC NMR (in benzene-*d*6) data of (-)-caprutriene B (**3a**)

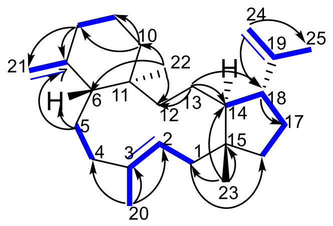

(-)-Caprudiene B (**3a**)

key COSY

key HMBC

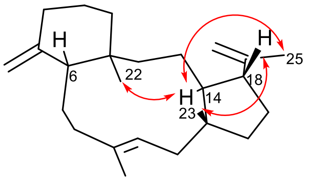

(-)-Caprutriene B (**3a**)

Key NOESY

Position	δH (ppm) (multiplicity, *J* in Hz, number of H)	δC (ppm)	COSY	HMBC	NOESY
1	2.12 (m, 1H);<br>1.98 (dd, 13.2, 2.0, 1H)	43.42	H2;<br>H2	C2, C3, C16, C23;<br>C2, C3, C14, C15, C23	H1b, H14;<br>H1a, H23
2	5.36 (m, 1H)	122.34	H1a, H1b,<br>H20	C4, C20	
3		136.13			
4	2.12 (m, 1H);<br>1.80 (dd, 13.2, 11.8, 1H)	39.34	H4b, H5a;<br>H4a, H5b	C6, C20;<br>C2, C3, C5, C6, C20	
5	1.70 (m, 1H);<br>1.34 (m, 1H)	23.01	H4a, H5b;<br>H4b, H5a	C6, C7;<br>C3, C6, C7, C11	
6	2.13 (m, 1H)	46.54	H21a, H21b	C7, C11, C21, C22	
7		150.28			
8	2.38 (dq, 13.2, 2.4, 1H);<br>1.89 (m, 1H)	38.44	H8b, H9a/b;<br>H8a,H9a/b	C6, C7, C11, C21;	
9	1.49 (m, 2H)	23.91	H8a, H8b	C11	
10	1.543 (m, 1H);<br>1.14 (m, 1H)	37.27	H10b;<br>H10a	C9;<br>C6, C8, C22	
11		39.52			
12	1.47 (m, 1H);<br>1.22 (m, 1H)	41.04	H12b;<br>H12a	C6, C10, C14;<br>C13, C14	
13	1.71 (m, 1H);<br>1.02 (m, 1H)	25.44	H13b;<br>H13a	C11, C12, C14;<br>C12, C18	
14	1.23 (m, 1H)	52.69	H18	C1, C12, C15, C18, C19, C23	H1a, H25
15		45.47			
16	1.48 (m, 2H)	42.63		C15, C17, C23	
17	1.61 (m, 1H);<br>1.48 (m, 1H)	29.41	H17b;<br>H17a, H18	C14, C15;<br>C16	
18	2.22 (m, 1H)	56.92	H14, H17b	C17	H13b, H23
19		147.7			
20	1.67 (s, 3H)	17.45	H2	C2, C3, C4	
21	4.99 (q, 2.4, 1H);<br>4.85 (br s, 1H)	150.28	H6, H21b;<br>H6, H21a	C6, C8;<br>C6, C8	
22	0.72 (s, 3H)	221.83		C4, C6, C10, C12	
23	0.93 (s, 3H)	23.3		C1, C14, C15	H1b, H13a, H14
24	4.94 (br d, 2.8, 1H);<br>4.80 (m, 1H)	111.16	H24b, H25;<br>H24a, H25	C18, C25;<br>C18, C25	
25	1.71 (s, 3H)	18.43	H24	C18, C19, C24	H5b, H10a, H10b,<br>H9a/b, H14

Table S6 | ^{1}H (400 MHz), ^{13}C (100 MHz), COSY, and HMBC NMR (in benzene-d_6) data of (+)-caprutriene C (**3b**)

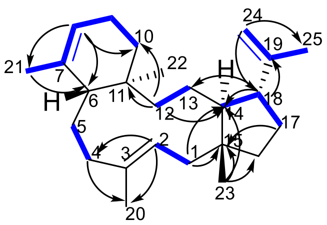

(+)-Caprutriene C (**3b**)

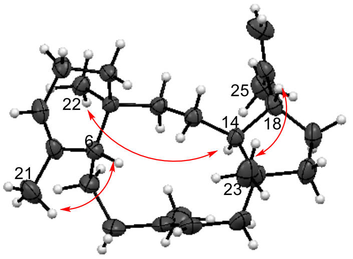

(+)-Caprutriene C (**3b**, X-ray)

key COSY
key HMBC
Key NOESY

Position	δH (ppm) (multiplicity, *J* in Hz, number of H)	δC (ppm)	COSY	HMBC	NOESY
1	2.20 (t, 12.0, 1H);<br>1.98 (d, 12.0, 1H)	43.72	H2	C2, C3, C14, C15, C16,<br>C23, C24, C25	
2	5.46 (dd, 12.0, 4.0, 1H)	124.07	H1a,b	C4, C20	
3		134.7			
4	1.48 (m, 2H)	42.47	H5	C5, C20	
5	1.36 (m, 2H)	24.64	H4	C3, C4, C7	
6	2.14 (dd, 12.4, 7.2, 1H)	42.56		C13, C21	H21
7		137.34			
8	5.42 (m, 1H)	121.66	H9, H21	C6, C10, C21	
9	2.06 (m, 1H);<br>1.87 (m, 1H)	23.06	H8, H10, H21	C6;<br>C7, C8, C11	
10	1.54 (m, 1H);<br>1.11 (m, 1H)	33.76	H9	C9, C11, C22;<br>C4, C8	
11		35.48			
12	1.39 (m, 1H);<br>1.08 (m, 1H)	41.67	H13a,b	C11, C22;<br>C10, C11, C14	
13	1.59 (m, 1H);<br>0.96 (m, 1H)	24.43	H12a<br>H12a	C11, C15, C18;<br>C18, C22	
14	1.13 (m, 1H)	53.12	H18	C1, C15, C18, C19	H22
15		45.89			
16	1.99 (m, 1H);<br>1.48 (m, 1H)	42.5		C15, C17, C18	
17	1.65 (m, 1H);<br>1.47 (m, 1H)	28.5	H18	C14, C15	
18	2.27 (dd, 11.2, 8.8, 1H)	56.45	H14, H17	C13, C14, C23, C25	H23
19		147.5			
20	1.68 (s, 3H)	16.66	H4	C2, C3, C4	
21	1.77 (s, 3H)	22.2	H8	C6, C7, C8	H6
22	0.80 (s, 3H);	21.73		C10, 11, C12	H14
23	0.90 (s, 3H)	21.94		C1, C14, C15	H18
24	4.92 (d, 2.8, 1H);<br>4.81 (dq, 2.8, 1.6, 1H)	111.1	H25	C18, C25	
25	1.70 (s, 3H)	18.3	H24	C18, C19, C24	

Table S7 | ^{1}H (400 MHz), ^{13}C (100 MHz), COSY, HMBC and NOESY NMR (in benzene-d_6) data of (-)-caprudiene (**4**)

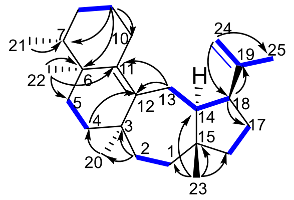

(-)-Caprudiene (**4**)

key COSY

key HMBC

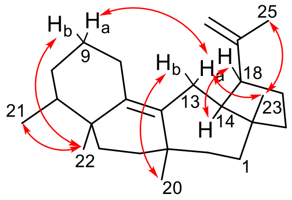

(-)-Caprudiene (**4**)

Key NOESY

Position	δH (ppm) (multiplicity, *J* in Hz, number of H)	δC (ppm)	COSY	HMBC	NOESY
1	1.65 (m, 1H);<br>1.49 (m, 1H)	40.62	H1b, H2a, H2b;<br>H1a, H2a, H2b	C2;<br>C3, C12	H1b, H2a;<br>H1a, H14
2	2.05 (td, 14.0, 4.8, 1H);<br>1.14 (m, 1H)	38.09	H1a, H1b, H2b;<br>H1a, H1b, H2a	C1, C3, C13, C23;<br>C23	H1a, H2b, H23;<br>H2a
3		38.54			
4	1.60 (m, 1H);<br>1.31 (m, 1H)	39.79	H4b;<br>H4a, H5a, H5b	C20;<br>C3, C12, C20	H4b, H20;<br>H4a, H20
5	1.62 (m, 1H);<br>1.50 (m, 1H)	34.73	H4b, H5b;<br>H4b, H5a	C7, C22;<br>C3, C6, C11, C12,<br>C22	H5b, H21, H22;<br>H4b, H5a
6		39.53			
7	1.33 (m, 1H)	45.29	H21		H8a, H21
8	1.70 (m, 1H);<br>1.35 (m, 1H)	27.13	H8b;<br>H8a, H9b		H8b, H7;<br>H8a, H21, H22
9	2.81 (d, 16.0, 1H);<br>1.79 (m, 1H)	26.63	H9b;<br>H8b, H9b	C6, C7, C10;<br>C6, C7	H8a, H9b, H10a, H13a;<br>H9a, H22
10	1.36 (m, 2H)	31.76			
11		137.8			
12		134.21			
13	2.67 (dd, 15.6, 11.2, 1H);<br>2.20 (ddd, 15.6, 8.0, 2.0, 1H)	29.3	H13b, H14;<br>H13a	C2, C14, C18;<br>C11, C12, C14, C15	H9a, H13b, H23, H25;<br>H13a
14	2.31 (dt, 11.2, 8.0, 1H)	47.92	H13a, H18	C1, C13, C15, C18,<br>C23	H5b, H13a, H18, H20
15		45.64			
16	1.42 (m, 1H);<br>1.36 (m, 1H)	43.58	H16b, H17a, H17b;<br>H16a, H17a, H17b	C14, C18, C23;<br>C14, C18, C23	
17	1.85 (m, 1H);<br>1.75 (m, 1H)	29.07	H16ab, H17b;<br>H16ab, H17a	C18, C19;<br>C14, C18	H18, H24b;<br>H24a
18	2.87 (t, 8.0, 1H)	50.43	H14	C14, C17, C19, C24,<br>C25	H14, H24a
19		147.8			
20	1.17 (s, 3H)	26.17		C2, C4, C12	H4a, H4b, H5b, H14
21	0.84 (d, 5.6, 3H);	16.24	H7	C6, C7, C10	H5a, H5b, H8b, H22
22	0.99 (s, 3H)	18.97		C5, C6, C11	H8b, H9b, H21
23	0.98 (s, 3H)	18.26		C1, C14, C15, C16	H2a, H13a, H25
24	5.02 (dq, 2.4, 1.8, 1H);<br>4.92 (br s, 1H)	112.83	H24b, H25;<br>H24a, H25	C18, C19, C25;<br>C18, C19, C25	H24b, H25;<br>H17a, H18, H24a, H23
25	1.77 (br s, 3H)	24.22	H24a,b	C18, C19, C24	H13a, H13b, H24a

Table S8 | ^{1}H (400 MHz), ^{13}C (100 MHz), COSY, HMBC and NOESY NMR (in benzene-d_6) data of (+)-brarapadiene A (**5**)

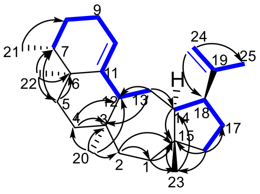

(+)-Brarapadiene A (**5**)

key COSY
key HMBC

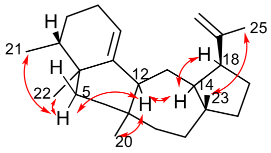

(+)-Brarapadiene A (**5**)

Key NOESY

Position	δH (ppm) (multiplicity, *J* in Hz, number of H)	δC (ppm)	COSY	HMBC	NOESY
1	1.57 (m, 1H);<br>1.29 (m, 1H)	37.68	H1b;<br>H1a	C3, C14, C15, C20;<br>C3, C12, C14, C15, C23	H1b, H23;<br>H1a, H20
2	1.62 (m, 2H)	33.71		C1, C14, C15	
3		37.88			
4	1.60 (m, 1H);<br>1.39 (m, 1H)	35.77		C2;<br>C3, C6, C7, C12, C20	H4b, H10, H20, H22;<br>H4a
5	1.51 (m, 1H);<br>1.31 (m, 1H)	38.16	H5b;<br>H5a	C22;<br>C6, C7, C22	H5b, H12, H21;<br>H5a
6		44.68			
7	1.40 (m, 1H)	39.42	H8a/b, H21	C6, C8, C9, C10	H21
8	1.41 (m, 2H)	26.72	H7	C7	H9a/b
9	2.08 (m, 2H)	25.82	H10, H12		H8a/b
10	5.60 (m, 1H)	117.36	H9, H12		H9a/b, H4a, H20;
11		149.25			
12	2.54 (m, 1H)	54.6	H9, H10, H13ab		H13a, H13b, H20, H22
13	1.79 (m, 1H);<br>1.58 (m, 1H)	24.69	H12, H13b;<br>H12, H13a	C3, C12;<br>C14	H12, H13b;<br>H12. H13a
14	1.69 (dd, 10.0, 8.0, 1H)	46.78	H18	C15, C18	H18, H20
15		41.89			
16	1.61 (m, 1H);<br>1.21 (m, 1H)	41.37	H16b;<br>H16a, H17	C14;<br>C19, C23	H2b, H17a/b, H23;<br>H2a, H17a/b
17	1.89 (m, 2H)	28.24	H16b, H18	C14, C16, C19	H16a, H16b, H18, H23, H24b
18	2.62 (dt, 10.0, 8.0, 1H)	46.87	H14, H17	C14, C19	H14, H17a/b
19		148.08			
20	0.99 (s, 3H)	20.09		C2, C3, C12, C13	H1b, H2a/b, H4ab, H12, H14
21	0.90 (d,6.0, 3H)	17.26	H7	C6, C7, C8	H5a, H7
22	0.890 (s, 3H)	20.6		C5, C6, C7, C11	H4a, H5a, H12
23	0.885 (s, 3H)	18.27		C14, C15	H1a, H16a, H25
24	4.96 (br s, 1H);<br>4.94 (br s, 1H)	111.45	H18, H24b, H25;<br>H18, H24a, H25	C14, C25;<br>C14, C25	H25;<br>H17a/b, H18, H23
25	1.76 (s, 3H)	25.35	H18, H24a, H24b	C18, C19, C24	H23, H24a

Table S9 | ^{1}H (400 MHz), ^{13}C (100 MHz), COSY, HMBC and NOESY NMR (in benzene-d_6) data of (-)-brarapadiene B (**6**)

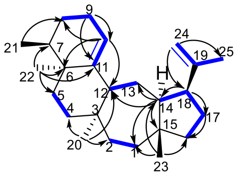

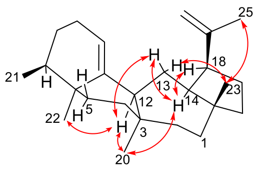

Position	δH (ppm) (multiplicity, *J* in Hz, number of H)	δC (ppm)	COSY	HMBC	NOESY
1	1.55 (m, 1H);<br>1.45 (m, 1H)	38.17	H1b;<br>H1a, H2b	C2, C3, C15, C16<br>C2, C14, C15, C23	H23;<br>H2a
2	1.64 (m, 1H);<br>0.95 (m, 1H)	34.04	H2b;<br>H1b	C1, C4, C20;<br>C4, C12, C15	H1b, H2b, H13a, H23;<br>H1a, H2a, H20
3		40.56			
4	1.09 (m, 1H);<br>0.81 (ddd, 12.8, 4.4, 2.0, 1H)	40.37	H4b, H5b;<br>H4a, H5a	C2, C3, C20;<br>C5, C6, C12, C20	H2a, H4b, H5a;<br>H4a
5	1.50 (m, 1H);<br>1.35 (m, 1H)	31.53	H4b, H5b;<br>H4a, H5a	C4, C6, C22;<br>C4, C6, C22	H4a;<br>H12, H20
6		39.07			
7	1.48 (m, 1H)	36.75	H21	C6	H21
8	1.36 (m, 2H)	27.2	H9	C6, C7	H9ab
9	2.04 (m, 2H)	26.22	H8	C8, C10, C11	H8ab
10	5.72 (br s, 1H);	119.42	H9, H12	C6, C8, C12	H9ab, H13a, H13b, H23
11		146.37			
12	2.21 (m, 1H)	43.69	H9, H13	C2, C3, C10, C11,<br>C13, C20	H13b, H14, H20, H22
13	1.80 (m, 1H);<br>1.71 (m, 1H)	26.43	H12, H13b;<br>H12, H13a	C4, C15;<br>C4, C15	H16a, H23;<br>H12, H14, H18
14	2.29 (td, 11.6, 1.6, 1H)	46.24	H15	C1, C12, C13, C15,<br>C19, C23	H13b, H16b, H18, H20
15		45.06			
16	1.56 (m, 1H);<br>1.30 (m, 1H)	43.15	H16b, H17a;<br>H16a	C14, C15, C18, C23;<br>C15, C17, C23	H13a, H16b, H23;<br>H13a, H14
17	1.77 (m, 2H)	29.69	H16a, H18;	C15, C18, C19;	H16ab, H24b
18	2.93 (dt, 11.6, 9.2, 1H)	50.65	H14, H17a	C14, C17, C19, C24,<br>C25	H13b, H14, H24b
19		147.88			
20	1.15 (s, 1H)	28.17		C2, C3, C4	H12, H14
21	0.86 (d, 7.6, 3H)	20.99	H7	C8	H5a, H8ab
22	0.87 (s, 3H)	16.75		C5, C6, C7, C11	H12
23	0.97 (s, 3H)	20.09		C1, C14, C16	H13a, H16a, H25
24	4.86 (dq, 4.0, 1.2, 1H);<br>4.83 (brs, 1H)	114.01	H24b, H25;<br>H24a, H25	C18, C25;<br>C18, C25	H25;<br>H18, H23
25	1.69 (br s, 3H)	23.01	H24a, H24b	C18, C19, C24	H23, H24a

Table S10 | ^{1}H (400 MHz), ^{13}C (100 MHz), COSY, HMBC and NOESY NMR (in benzene-d_6) data of (-)-arathanadiene A (**7**)

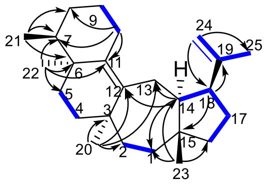

(-)-Arathanadien A (**7**)

key COSY

key HMBC

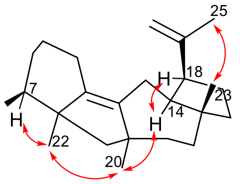

(-)-Arathanadiene A (**7**)

Key NOESY

Position	δH (ppm) (multiplicity, *J* in Hz, number of H)	δC (ppm)	COSY	HMBC	NOESY
1	1.49 (m, 1H);<br>1.33 (m, 1H)	37.63	H1b, H2a, H2b;<br>H1a	C2, C16, C23;<br>C15, C17, C23	H1b;<br>H1a, H2b, H14
2	1.78 (dd, 14.4, 8.8, 1H);<br>1.52 (m, 1H)	33.03	H1a, H2b;<br>H1a, H2a	C3, C12, C20;<br>C2, C13, C15	H2b, H23;<br>H1b, H2a, H10a, H20
3		37.38			
4	2.43 (m, 2H)	31.54	H5a, H5b	C5, C12	H5a, H5b, H13a/b, H20, H22
5	1.63 (m, 1H);<br>1.50 (m, 1H)	37.65	H4ab, H5b;<br>H4ab, H5a	C4, C5b, C6, C12, C22;<br>C4, C5a, C6, C7, C22	H4a/b, H5b, H21;<br>H4a/b, H5a, H21
6		51.66			
7	1.35 (m, 1H)	44.55	H21	C21	H21, H22
8	1.34 (m, 1H);<br>1.26 (m, 1H)	30.89		C10;	H22
9	1.68 (m, 1H);<br>1.27 (m, 1H)	27.34	H9b, H10a;<br>H9a		H10a, H20
10	2.79 (dt, 14.8, 3.2, 1H);<br>1.85 (m, 1H)	25.78	H9a, H10b;<br>H10a	C6, C8, C11, C12;<br>C3, C7	H2b, H9a, H10b, H20;<br>H10a
11		140.94			
12		140.77			
13	1.74 (m, 2H)	35.82		C2, C3, C15, C23	H4a/b, H23
14	1.74 (m, 1H)	46.6	H18	C13, C18, C9, C20, C23	H1b, H10a, H16b, H20
15		41.81			
16	1.58 (ddd, 11.6, 6.8, 2.0, 1H);<br>1.18 (m, 1H)	41.4	H16b, H17ab;<br>H16a, H17ab	C13, C14, C25;<br>C15, C19, C23	H16b, H23;<br>H16a, H17ab
17	1.89 (m, 2H)	28.11	H16ab	C13, C14, C15, C19, C25	H16b, H18, H23, H24b
18	2.60 (m, 1H)	46.74	H14, H17ab	C14, C19, C24, C25	H14, H17a/b, H24b
19		148			
20	1.23 (s, 3H)	24.51		C2, C3, C12, C13	H2b, H4a/b, H14, H22
21	0.83 (d, 6.4, 3H)	17	H7	C6, C7, C8	H5a, H5b, H7
22	0.89 (s, 3H)	17.27		C5, C6, C7, C11	H7, H20
23	0.89 (s, 3H);	18.34		C1, C14, C16	H13a/b, H16a, H25
24	4.95 (s, 1H);<br>4.93 (s, 1H)	111.4	H24b, H25;<br>H24a, H25	C24b, C25;<br>C24a, C25	H25;<br>H13a/b, H17a/b, H18, H23
25	1.75 (s, 3H)	25.31	H24ab	C18, C19, C24	H23, H24a

Table S11 | ^{1}H (400 MHz), ^{13}C (100 MHz), COSY, HMBC and NOESY NMR (in benzene-d_6) data of (-)-arathanadiene B (**8**)

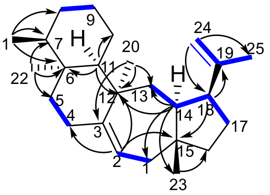

(-)-Arathanadiene B (**8**)

key COSY
key HMBC

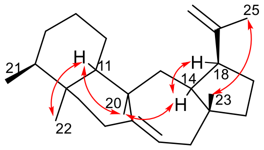

(-)-Arathanadiene B (**8**)

Key NOESY

Position	δH (ppm) (multiplicity, *J* in Hz, number of H)	δC (ppm)	COSY	HMBC	NOESY
1	2.02 (m,2H)	41.94	H2	C2, C3, C4, C14, C15, C23	H2, H14, H16ab, H20, H23
2	5.40 (ddd, 8.4, 4.8, 1.2, 1H)	119.47	H1ab	C4, C12, C15	H2ab, H4b, H23
3		150.87			
4	2.40 (br t, 9.2, 2H)	32.95	H4b, H5b;<br>H4a, H5b	C2, C3, C5, C6;<br>C2, C3, C6, C12	H4b, H11, H20, H22;<br>H2, H4a
5	1.72 (m, 1H);<br>1.36 (m, 1H)	40.61	H5b;<br>H4a, H4b, H5a	C4, C6, C12, C22;<br>C3, C6, C7	H5b, H22;<br>H5a, H10a, H21, H22
6		36.78			
7	1.63 (m, 1H)	40.7	H21	H8	H21
8	1.49 (m, 1H);<br>1.28 (m, 1H)	30.9	H8b, H9ab;<br>H8a	C6, C7;<br>C6, C7, C21	H8b, H21;<br>H8a, H9ab, H22
9	1.69 (m, 2H)	21.94	H8a	C10, C11	H8b, H20, H22
10	1.79 (m, 1H);<br>1.63 (m, 1H)	24.92		C9, C20;<br>C11, C20	H5b, H10b;<br>H10a
11	1.47 (m, 1H)	50.51		C6, C10, C12	H4a, H20, H22
12		44.05			
13	1.76 (m, 1H);<br>1.60 (m, 1H)	34.05	H13b;<br>H13a, H14	C3, C12, C14, C15, C20;<br>C12, C14, C15	H13b;<br>H13a, H23
14	2.15 (ddd, 13.2, 11.6, 2.4, 1H)	50.43	H13b, H18	C1, C12, C13, C18, C23	H1ab, H16b, H18, H20
15		42.64			
16	1.48 (m, 1H);<br>1.31 (m, 1H)	42.14	H16b;<br>H16a	C14;<br>C17	H16b, H23;<br>H1ab, H14, H16a
17	1.75 (m, 2H)	28.54	H18		
18	2.90 (dt, 11.6, 8.8, 1H)	51.32	H14, H17ab	C14, C15, C17, C19, C25	H18
19		148.23			H14, H17ab, H24b
20	1.15 (s, 3H)	26.02		C3, C11, C12, C13	H1a/b, H9a/b, H11, H14
21	0.82 (d, 6.8, 3H)	16.85	H7	C6, C7, C8	H7
22	0.86 (s, 3H);	21.59		C5, C6, C11	H4a, H5a, H8b, H9a/b, H11
23	0.94 (s, 3H)	19.35		C14, C16	H1a/b, H13b, H16a, H17a/b, H25
24	4.92 (dq, 2.4, 1.8, 1H);<br>4.84 (d, 2.4, 1H)	113.68	H25	C18, C25;<br>C18, C25	H24b, H25;<br>H2, H13b, H23, H25
25	1.73 (s, 3H)	23.09	H24ab	C18, C19, C24	H23, H24a, H24b

Table S12 | ^{1}H (400 MHz), ^{13}C (100 MHz), COSY, HMBC and NOESY NMR (in benzene-d_6) data of (-)-brarapone B (**9**)

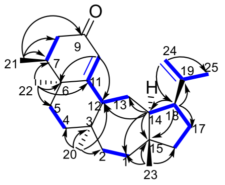

(-)-Brarapone B (**9**)

key COSY

key HMBC

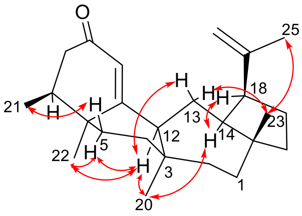

(-)-Brarapone B (**9**)

Key NOESY

Position	δH (ppm) (multiplicity, *J* in Hz, number of H)	δC (ppm)	COSY	HMBC	NOESY
1	1.43 (m, 1H);<br>1.34 (m, 1H)	37.88	H1b, H2b;<br>H1a, H2b	C2, C15, C23<br>C2, C3, C15	H2b;
2	1.43 (m, 1H);<br>0.84 (m, 1H)	34.15	H2b;<br>H1a, H1b, H2b	C1, C3, C20;<br>C3, C20	H14, H20;<br>H5a, H20
3		41.47			
4	0.85 (m, 1H);<br>0.68 (m, 1H)	39.81	H4b, H5b;<br>H4a, H5a	C2, C20;<br>C6, C12, C20	H4b;<br>H4a, H5a, H5b, H20
5	1.30 (m, 1H);<br>1.18 (m, 1H)	30.86	H4b, H5b;<br>H4a, H5a	C7, C11;<br>C4, C6, C7, C20, C22	H4b, H5b, H21;<br>H1b, H4b, H5a, H12, H20, H21
6		41.04	H5, H21		
7	1.73 (m, 1H)	36.34	H8a,b, H21	C11, C21, C22	H21
8	2.25 (dd, 13.6, 4.4, 1H);<br>2.02 (dd, 16.8, 13.6, 1H)	42.1	H7, H8b;<br>H7, H8a	C6, C9;<br>C7, C9, C21	H7, H8b, H21;<br>H8a, H21
9		197.22			
10	6.34 (s, 1H)	125.17	H12	C6, C12	H13a, H13b, H20
11		174			
12	2.11 (ddd, 10.0, 8.0, 2.4, 1H);	44.38	H10, H13a, H13b	C2, C3, C10, C11, C13, C20	H5b, H13b, H20, H22
13	1.72 (m, 1H);<br>1.64 (m, 1H)	26	H12, H13b;<br>H12, H13a	C15;<br>C3, C15, C19	H23, H25;<br>H12, H24a
14	2.19 (td, 11.6, 2.4, 1H)	45.65	H18	C1, C12, C13, C18, C19, C23	H2a, H16b, H18, H20
15		44.94			
16	1.49 (m, 1H);<br>1.25 (m, 1H)	42.98	H16b;<br>H16a, H17a, H17b	C18;<br>C15, C23	H16b;<br>H14, H16a, H17ab
17	1.72 (m, 2H)	29.48	H16b, H17b, H18;<br>H16b, H17a, H18	C19, C25;<br>C19, C25	H16b;<br>H16b
18	2.89 (dt, 11.6, 9.2, 1H)	50.71	H14, H17a, H17b	C15, C19, C24, C25	H14, H17, H24, H25
19		147.57			
20	1.03 (s, 1H)	28.2		C2, C3, C4, C12	H2a, H5b, H12, H14
21	0.60 (d, 6.8, 3H)	18.96	H7	C6, C7, C8	H5a, H7, H8a
22	0.58 (s, 3H)	15.84		C5, C6, C7, C11	H5b, H8b, H12
23	0.85 (s, 3H)	20.69		C1, C15, C16	H13a, H16a, H25
24	4.81 (m, 1H);<br>4.78 (d, 2.4, 1H)	114.42	H24b, H25;<br>H24a, H25	C18, C24b, C25;<br>C18, C24a, C25	H13a, H18, H23, H25;<br>H13b
25	1.58 (d, 0.8, 3H)	22.5	H24a, H24b	C18, C19, C24	H23, H24a

(A) Computed ECD spectrum

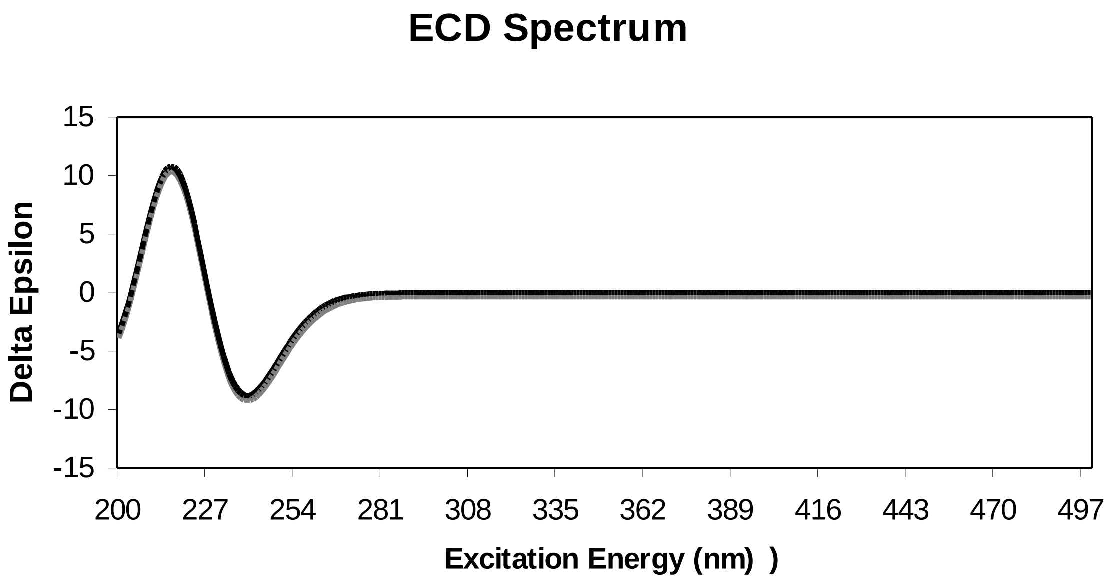

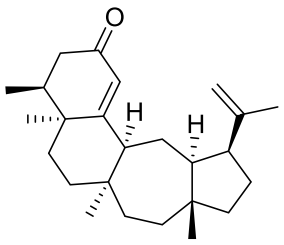

(-)-Brarapone B (**9**)

(B) Experimental ECD spectrum of (-)-brarapone B (9) (in EtOH, 0.5 mg/ml)

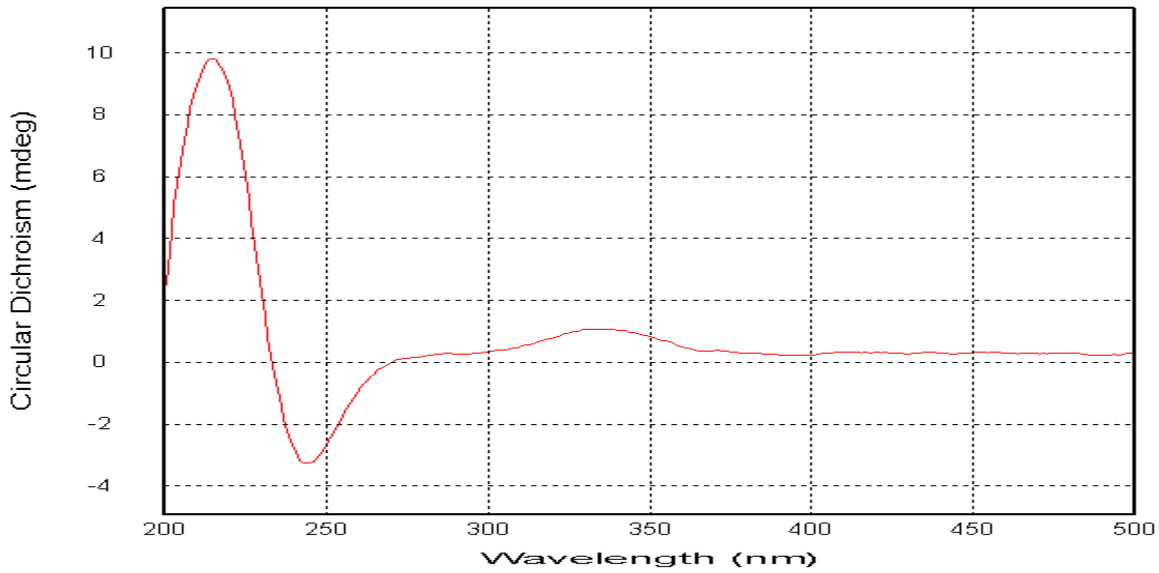

Figure S1. Calculated ECD spectrum (A) and experimental ECD spectrum (B) of (-)-brarapone B (**9**)

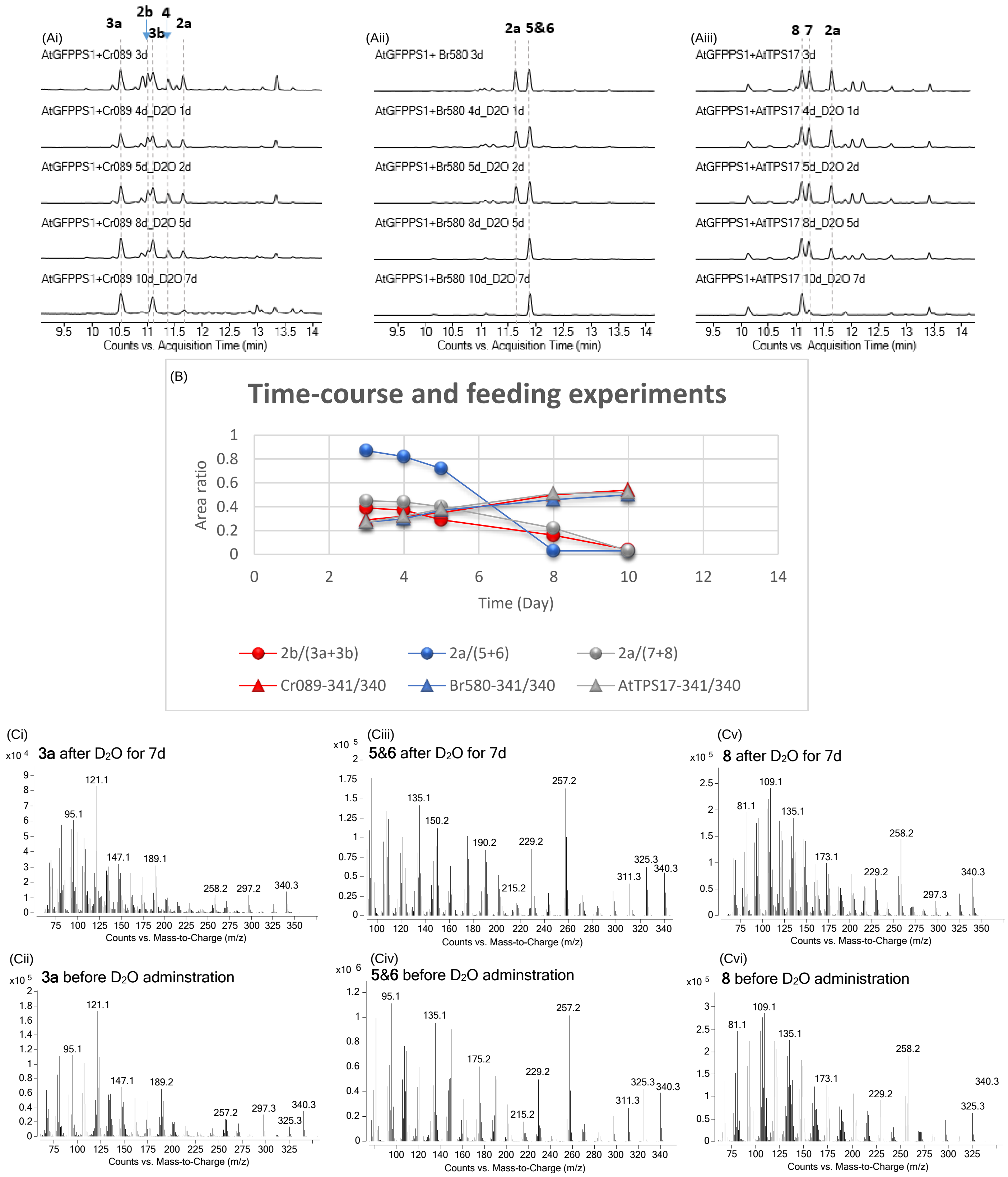

Figure S2. Time course and in vivo feeding experiments in *N.benthamina*. A) GC-MS traces of extracts of leaves expressing different STSs harvested on the 3rd, 4th, 5th, 8th and 10th day post agro-infiltration. Plants were water with D_2O after sampling the first leaf on the 3rd day. Ai, AtGFPPS1+Cr089; Aii, AtGFPPS1+Br580; Aiii, AtGFPPS1+AtTPS17. B) Time-course experiment showing the area ratios of compounds **2b**/(**3a**+**3b**), **2a**/(**5**+**6**) and **2a**/(**7**+**8**) decreased over time whilst those of ms fragment 341/340 increased. C) MS spec of compounds **3a** (i & ii), **5**+**6** (iii & iv), and **8** (v & vi) before and after feeding D_2O indicates incorporation deuteria in the products.

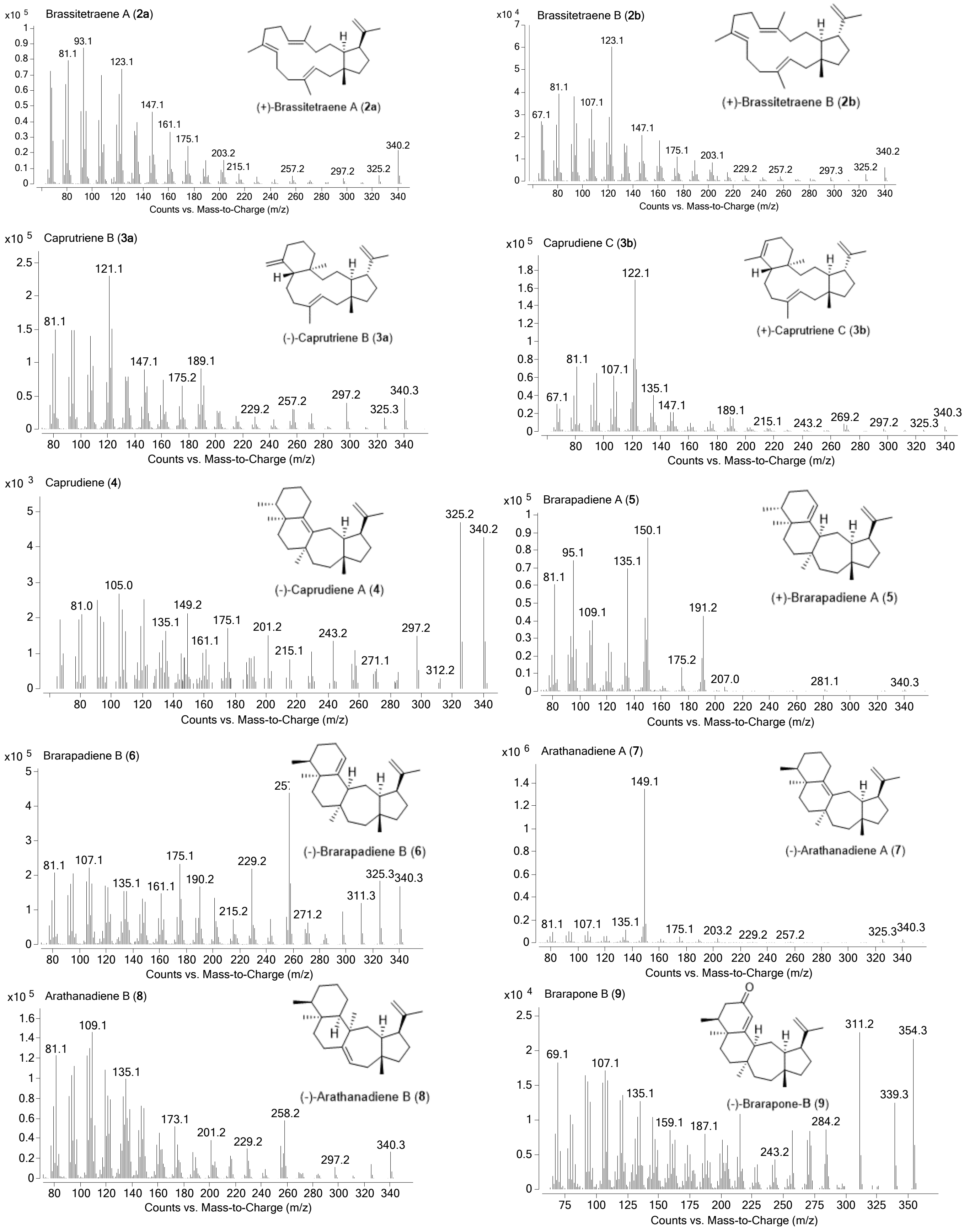

Figure S3. Mass spectra (EI-MS) of compounds **2**-**9**

(A)

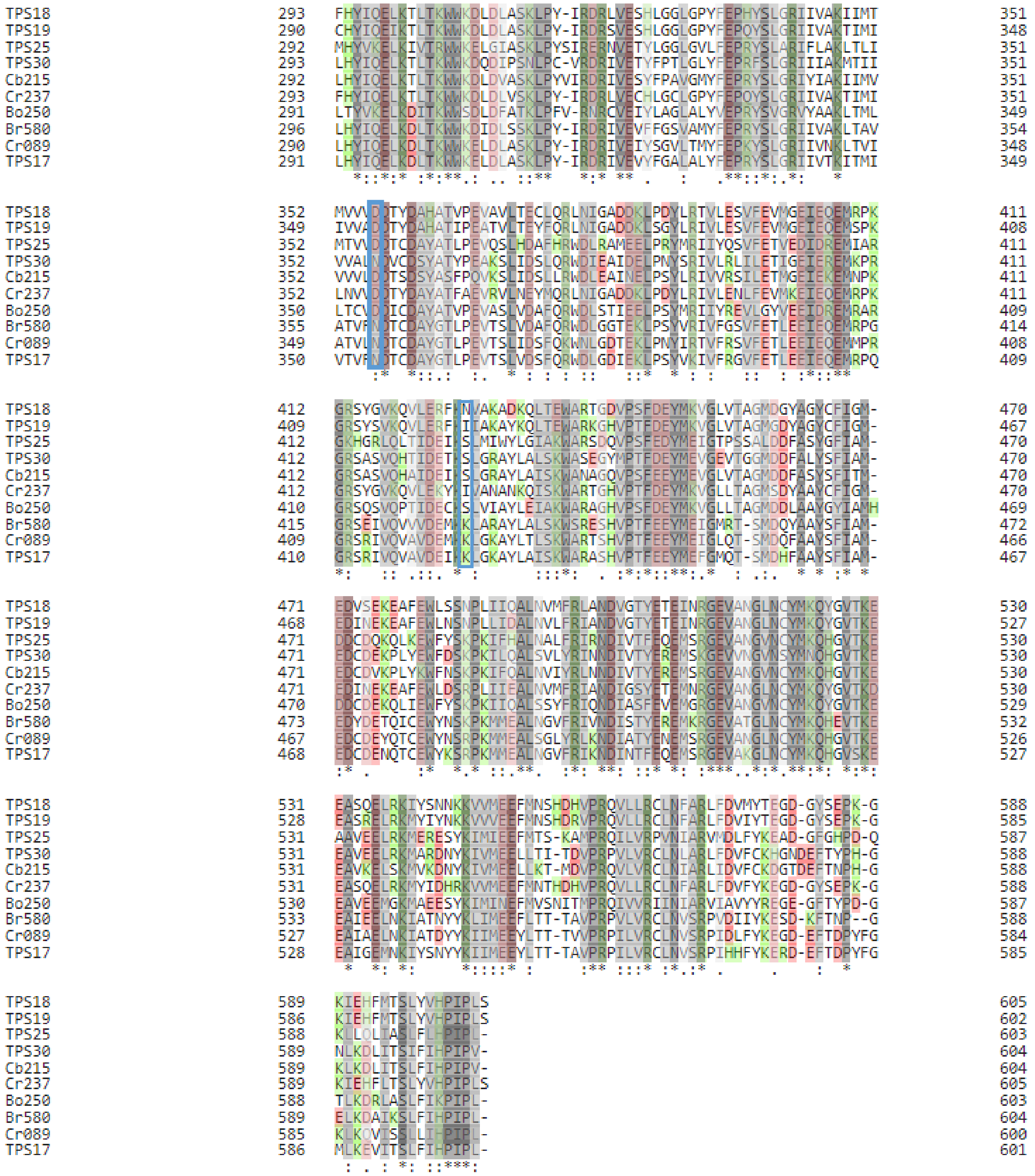

Figure S4. Sequence and structural analysis of the TPSs investigated in this work. A) Sequence alignment of the N-terminus region of protein sequences of the characterized plant STSs with the four investigated in this work suggested two sites (highlighted in blue boxes) implicated in driving the functional divergence of plant STSs. Sequences were aligned in uniprot (http://www.uniprot.org/align/). Gray shading indicates amino acids in similarity, red shading denotes amino acids with negative charge; green shading refers to amino acids with positive charge.

(B)

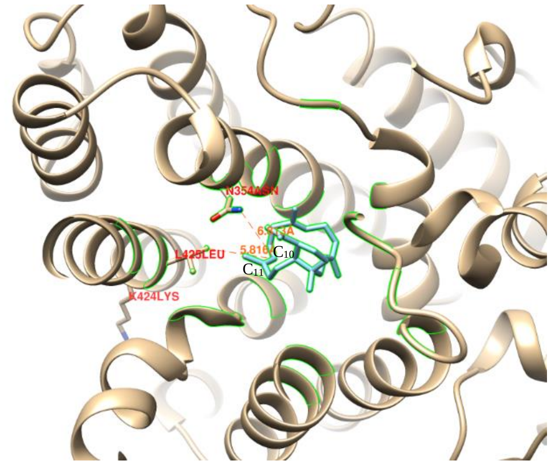

(C)

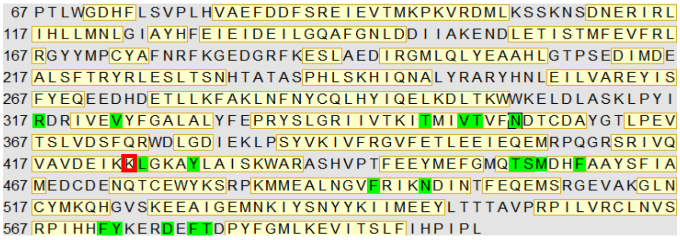

Figure S4. Sequence and structural analysis of the TPSs investigated in this work**.** B) Homology model of AtTPS17 with brassitetraene (**2a**) docked in the active site. The homology model was created based on the crystal structure of 5-epi-aristolochene synthase in the Phyer^2 server (http://www.sbg.bio.ic.ac.uk/phyre2/html/page.cgi?id=index). The two key amino acid residues (N354 and K424) identified from sequence comparison and a residue L425 adjacient to K424 were labelled in red. Amino acid residues within 5 Å region of the substrate were highlighted in green. C) Amino acid residues within 5 Å region of the docked substrate were highlighted in green in the sequence. Lysine424 (K424) is highlighted in red box.

*Note: the homology model is generated based on the crystal structure of a sesquiterpene synthase and is for understanding the approximate positions of some key amino acids only, the exact positions of amino acid residues in the sesterterpene synthase AtTPS17 will differ from the homology model.

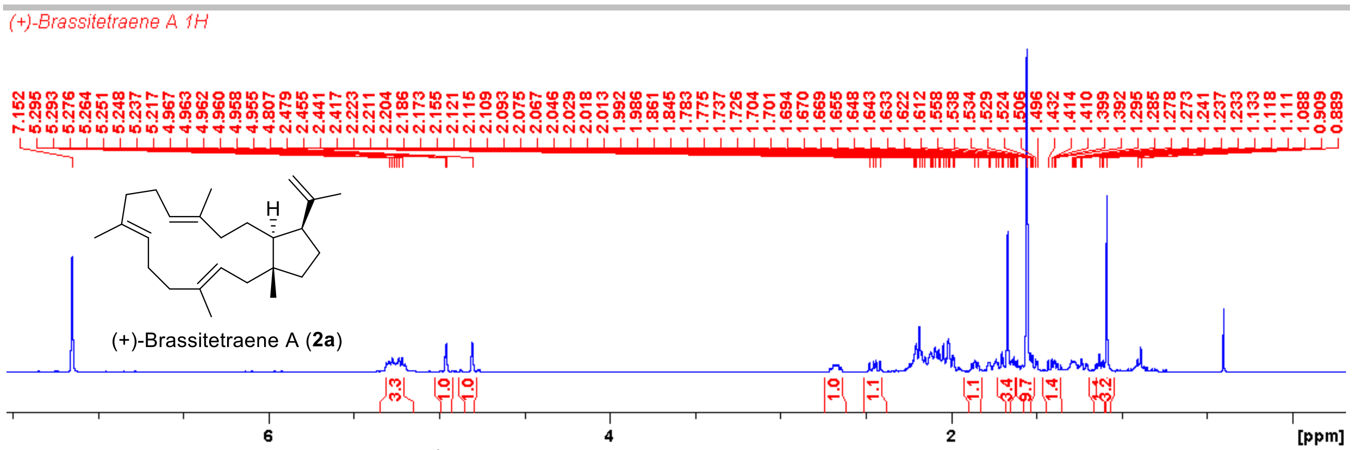

Figure S5. ^{1}H NMR (400 MHz, benzene-d$_6$) spectrum of (+)-brassitetraene A (**2a**)

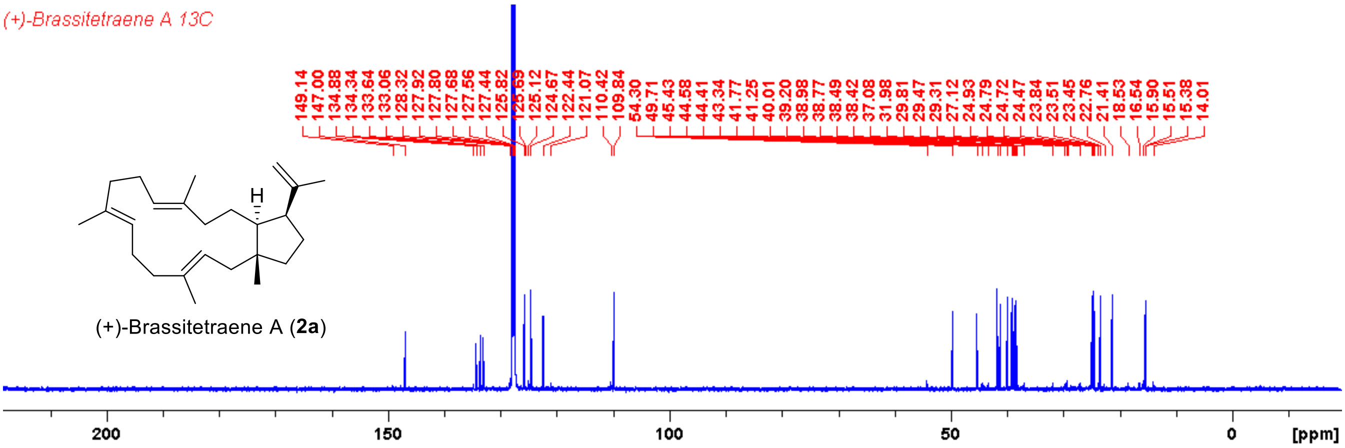

Figure S6. ^{13}C NMR (100 MHz, benzene-d$_6$) spectrum of (+)-brassitetraene A (**2a**)

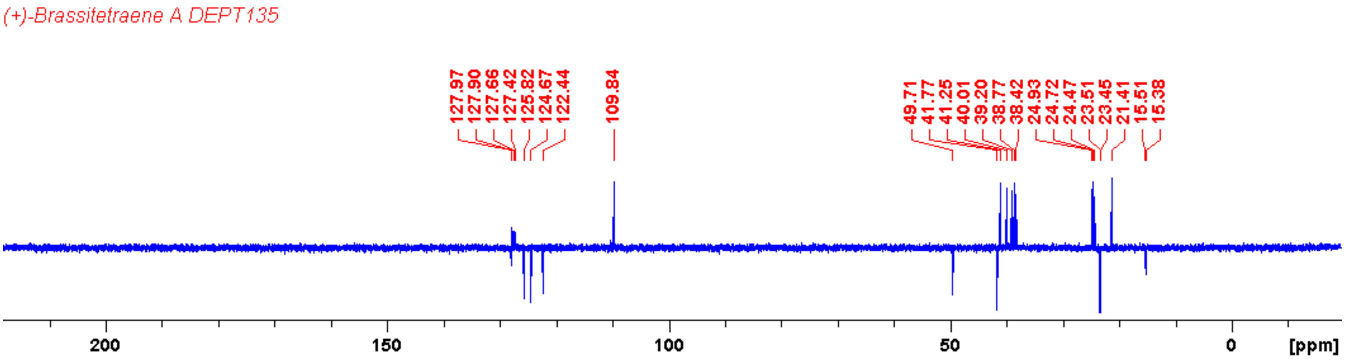

Figure S7. DEPT135 (100 MHz, benzene-d$_6$) spectrum of (+)-brassitetraene A (**2a**)

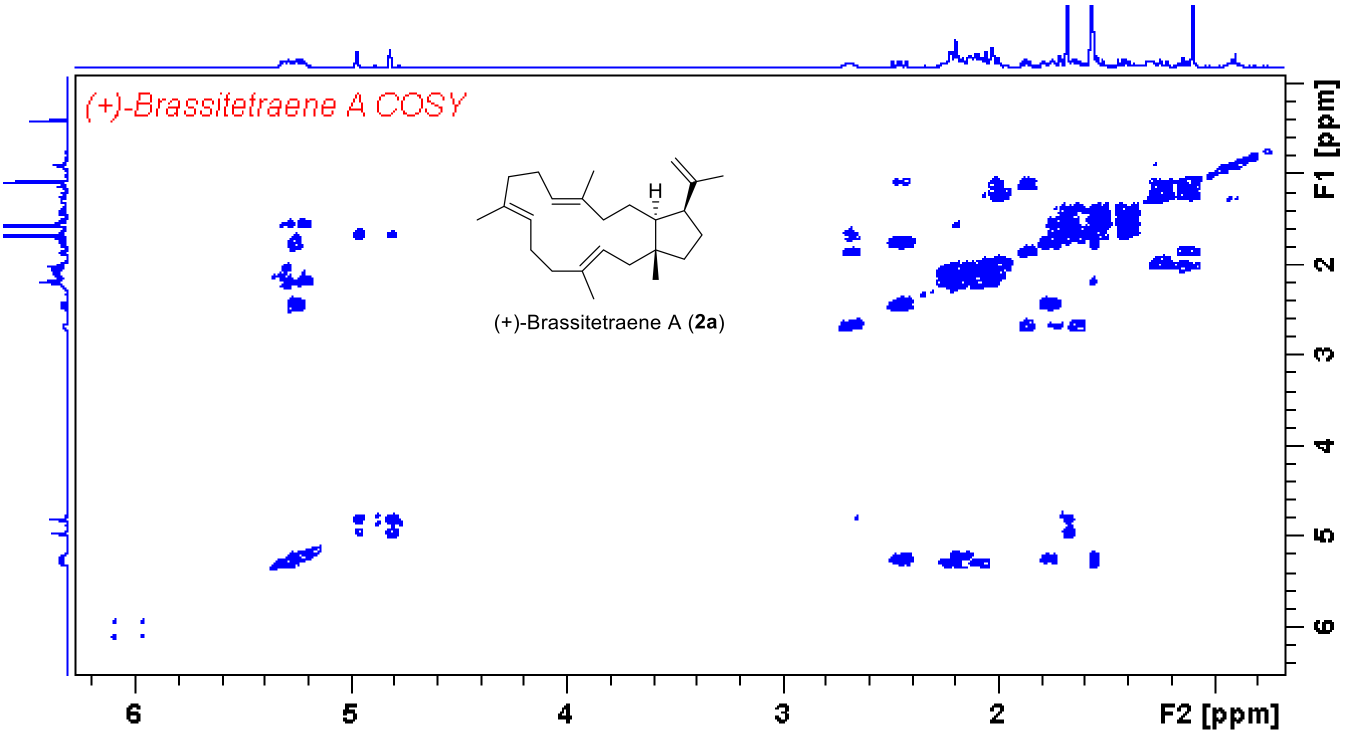

Figure S8. COSY NMR (400 MHz, benzene-d6) spectrum of (+)-brassitetraene A (**2a**)

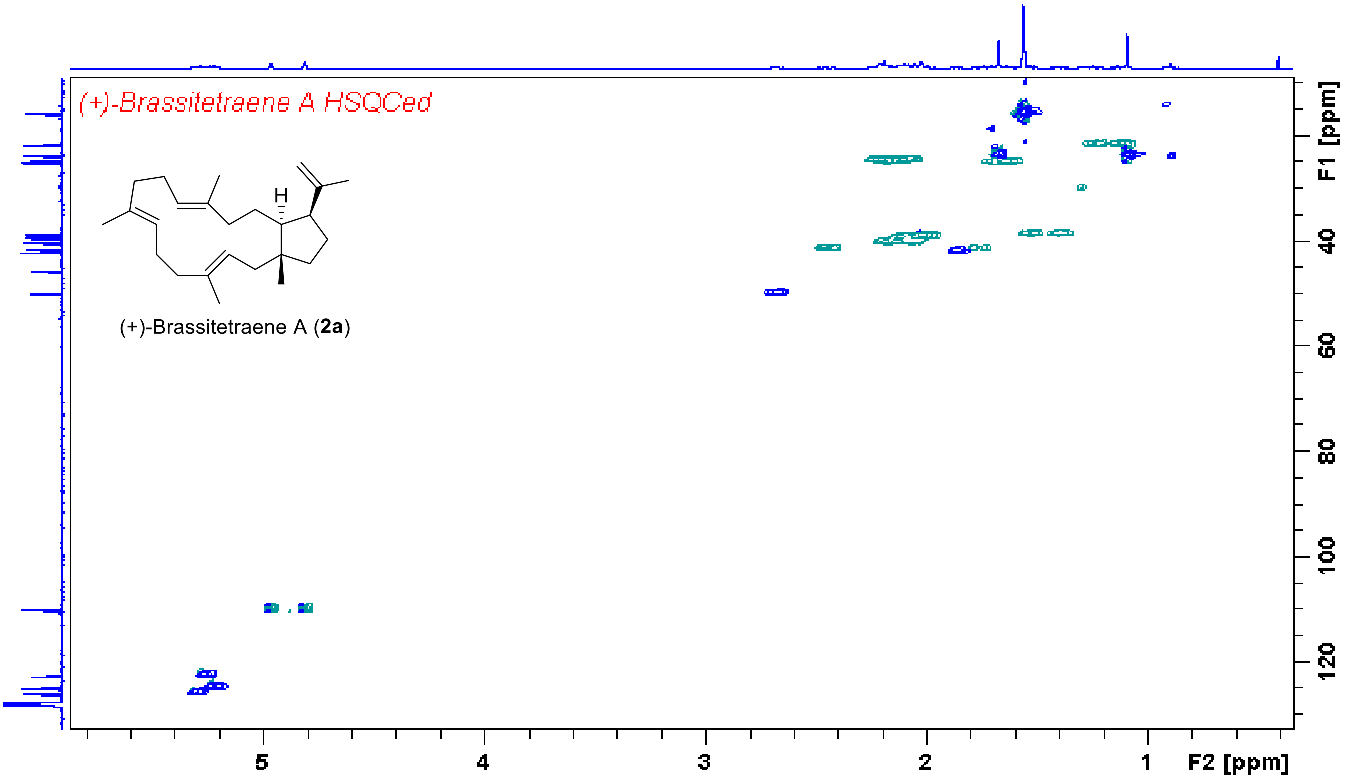

Figure S9. HSQC NMR (400 MHz, benzene-d6) spectrum of (+)-brassitetraene A (**2a**)

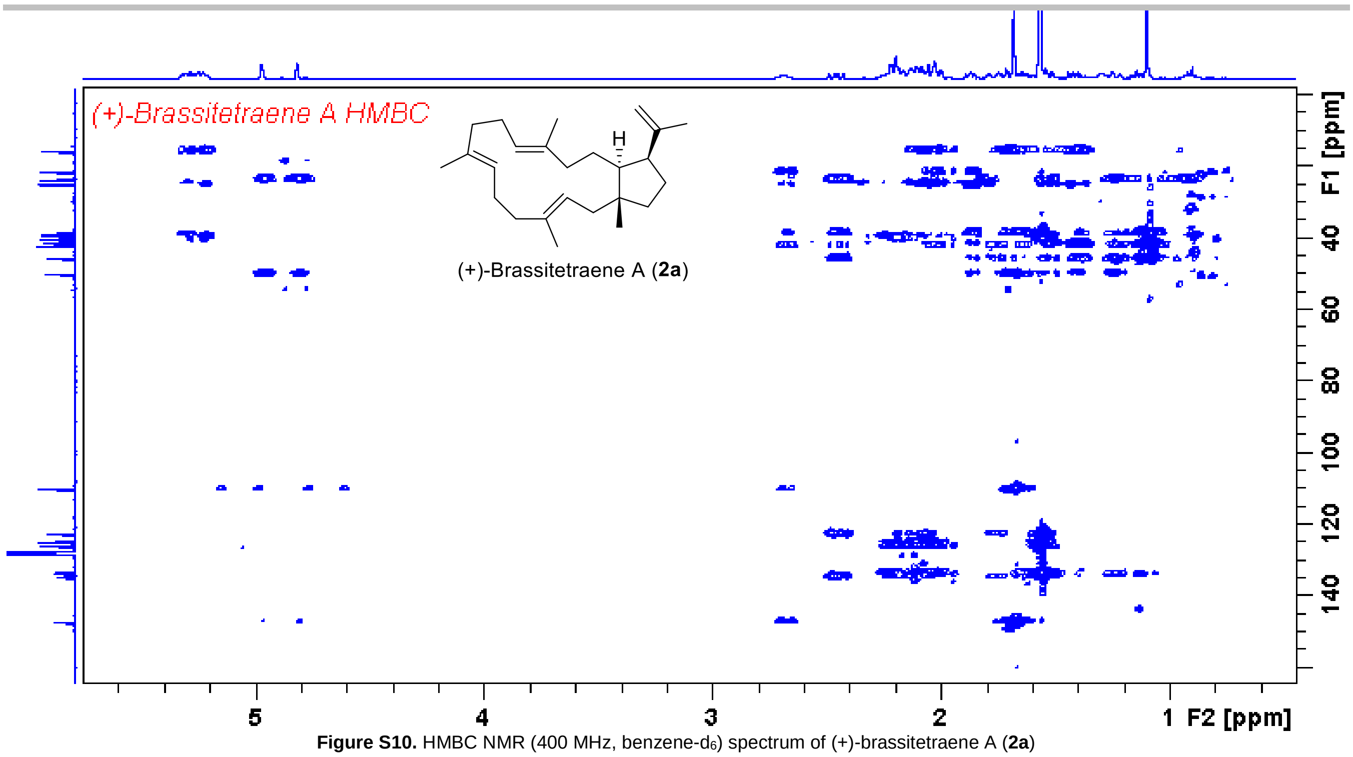

Figure S10. HMBC NMR (400 MHz, benzene-d6) spectrum of (+)-brassitetraene A (**2a**)

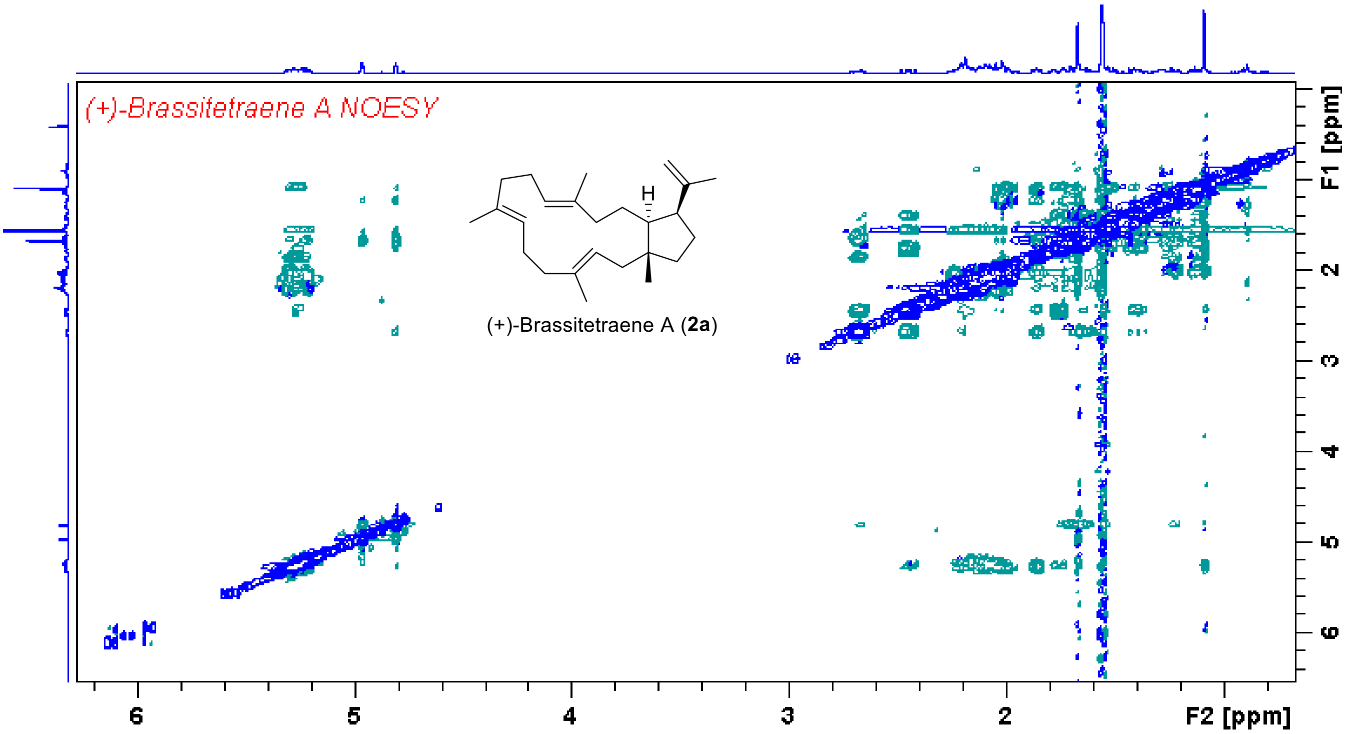

Figure S11. NOESY NMR (400 MHz, benzene-d6) spectrum of (+)-brassitetraene A (**2a**)

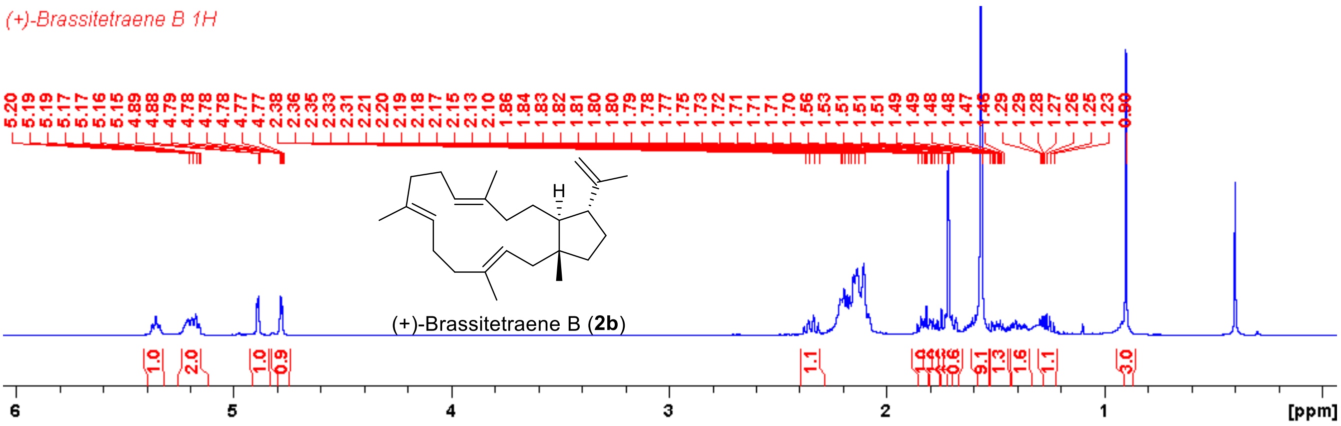

Figure S12. ^{1}H NMR (400 MHz, benzene-d$_6$) spectrum of (+)-brassitetraene B (**2b**)

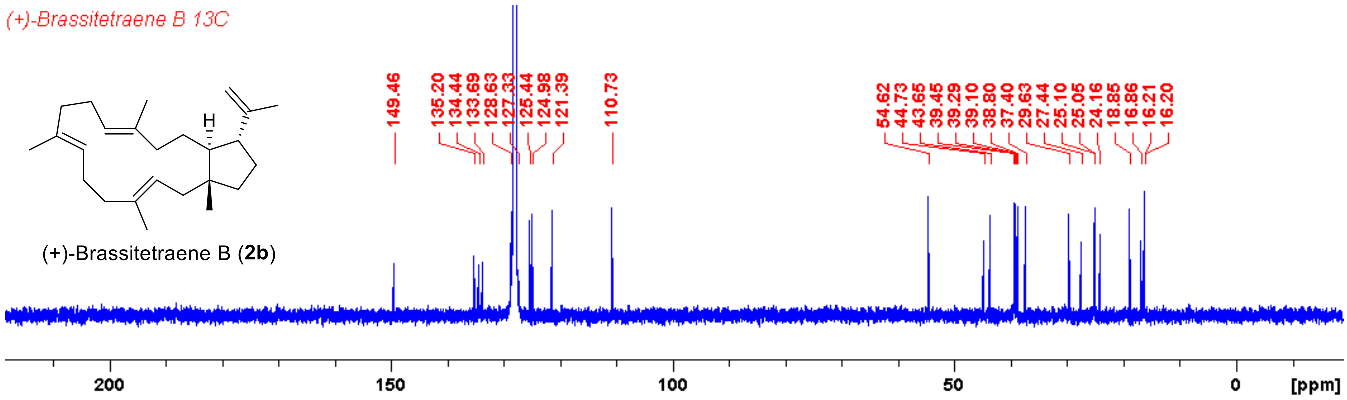

Figure S13. ^{13}C NMR (100 MHz, benzene-d$_6$) spectrum of (+)-brassitetraene B (**2b**)

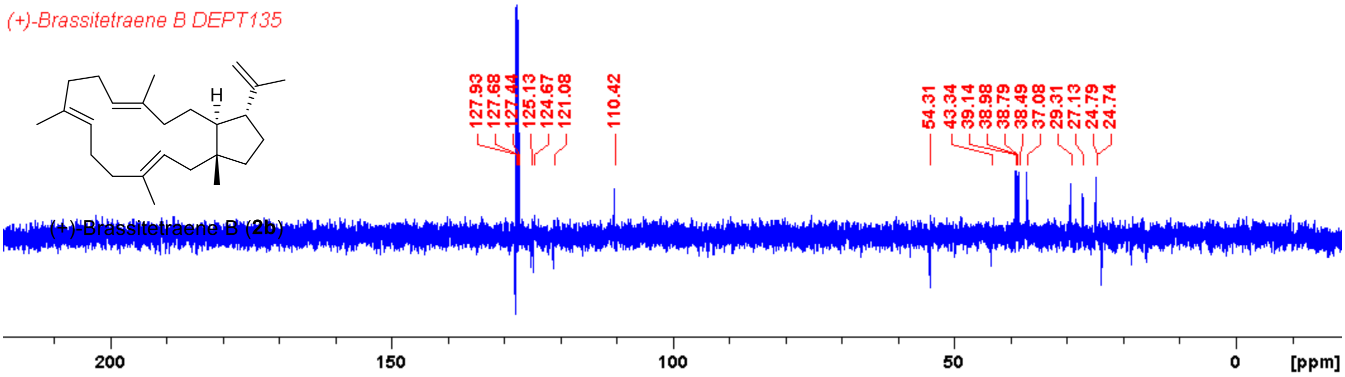

Figure S14. DEPT135 NMR (100 MHz, benzene-d$_6$) spectrum of (+)-brassitetraene B (**2b**)

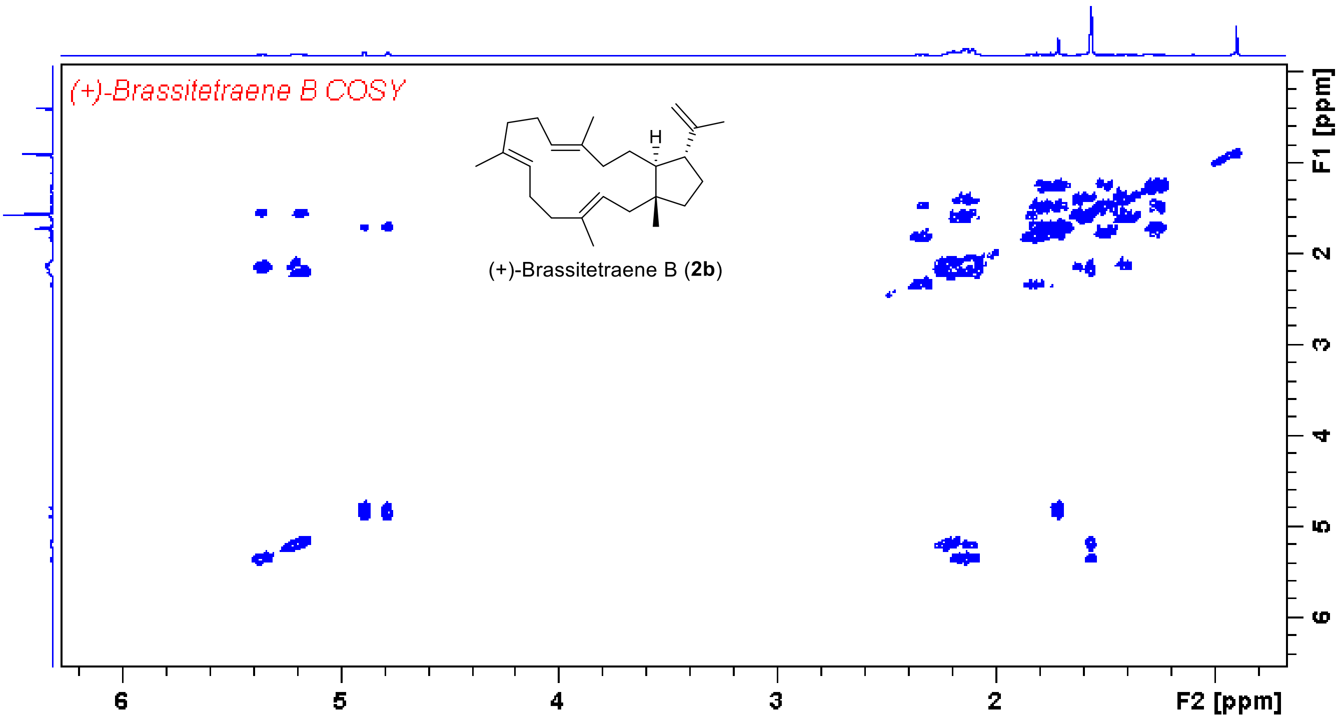

Figure S15. COSY NMR (400 MHz, benzene-d_6) spectrum of (+)-brassitetraene B (**2b**)

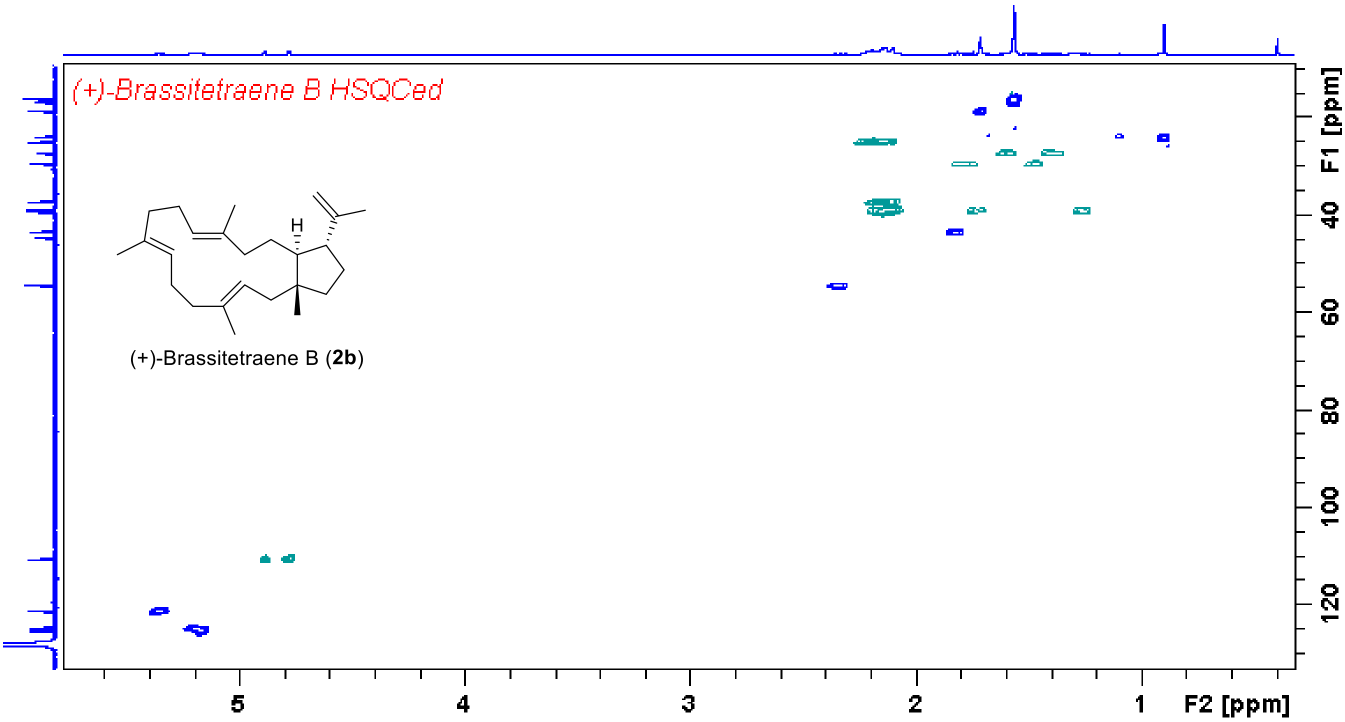

Figure S16. HSQC NMR (400 MHz, benzene-d_6) spectrum of (+)-brassitetraene B (**2b**)

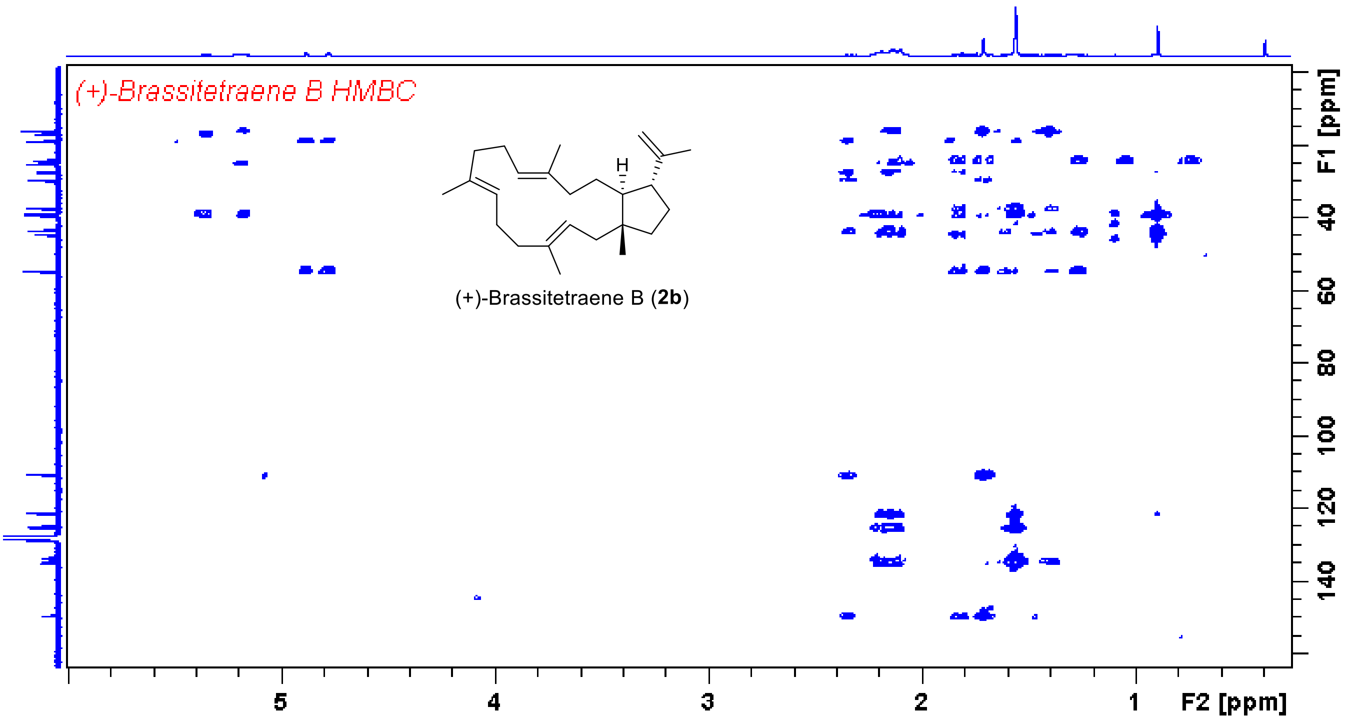

Figure S17. HMBC NMR (400 MHz, benzene-d_6) spectrum of (+)-brassitetraene B (**2b**)

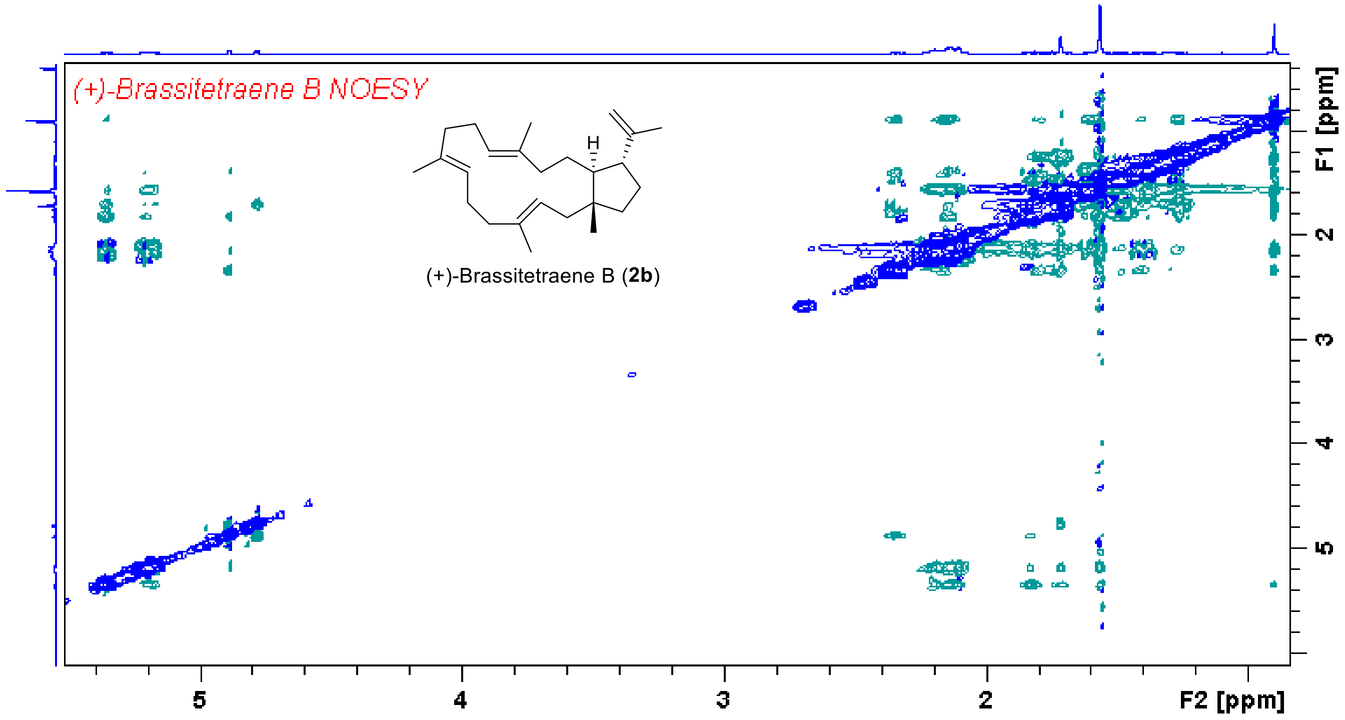

Figure S18. NOESY NMR (400 MHz, benzene-d_6) spectrum of (+)-brassitetraene B (**2b**)

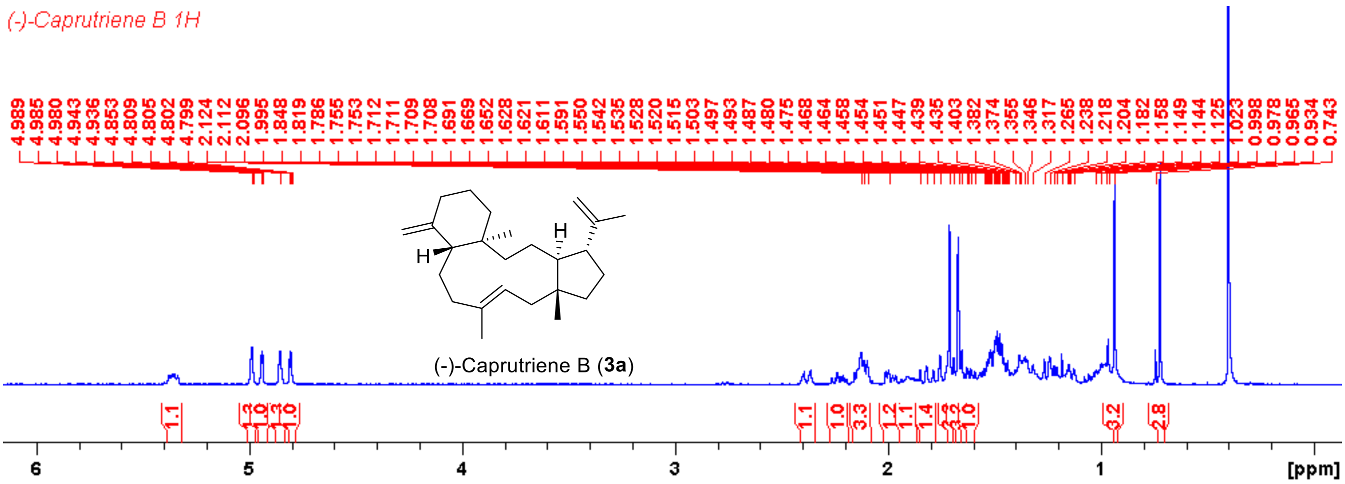

Figure S19. ^{1}H NMR (400 MHz, benzene-d$_6$) spectrum of (-)-caprutriene B (**3a**)

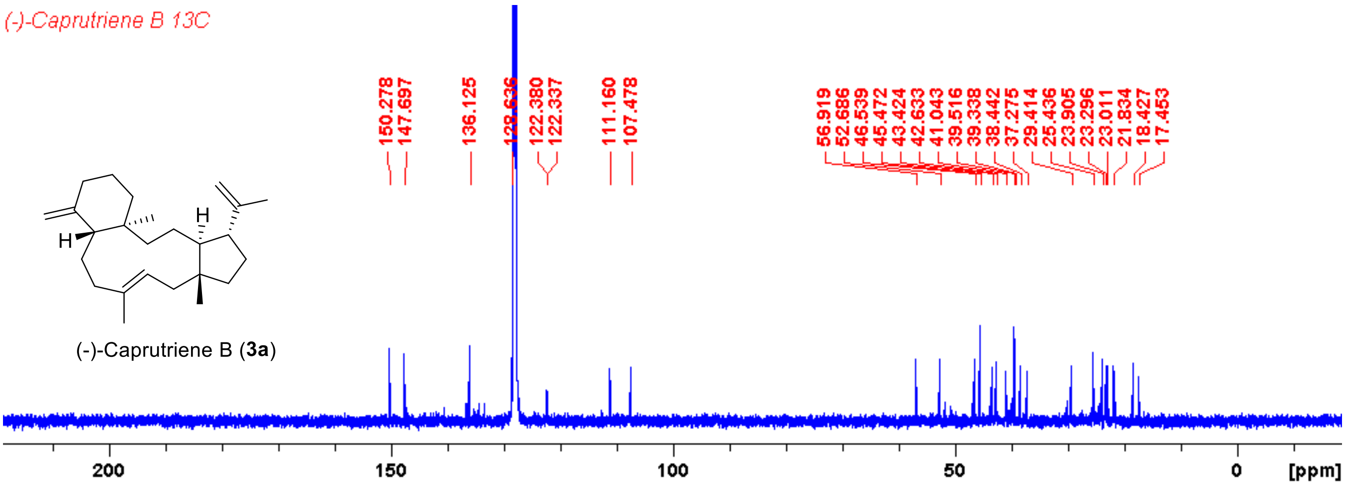

Figure S20. ^{13}C NMR (100 MHz, benzene-d$_6$) spectrum of (-)-caprutriene B (**3a**)

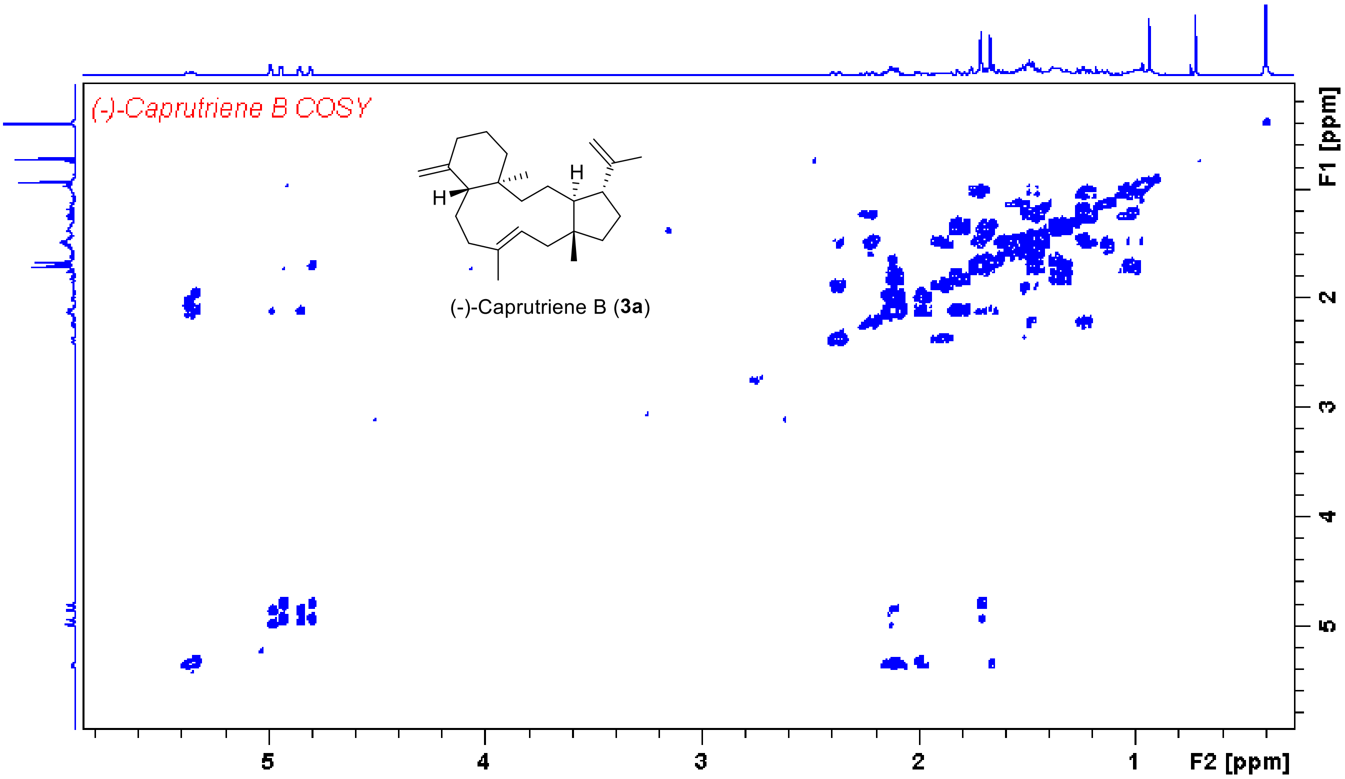

Figure S21. COSY NMR (400 MHz, benzene-d_6) spectrum of (-)-caprutriene B (**3a**)

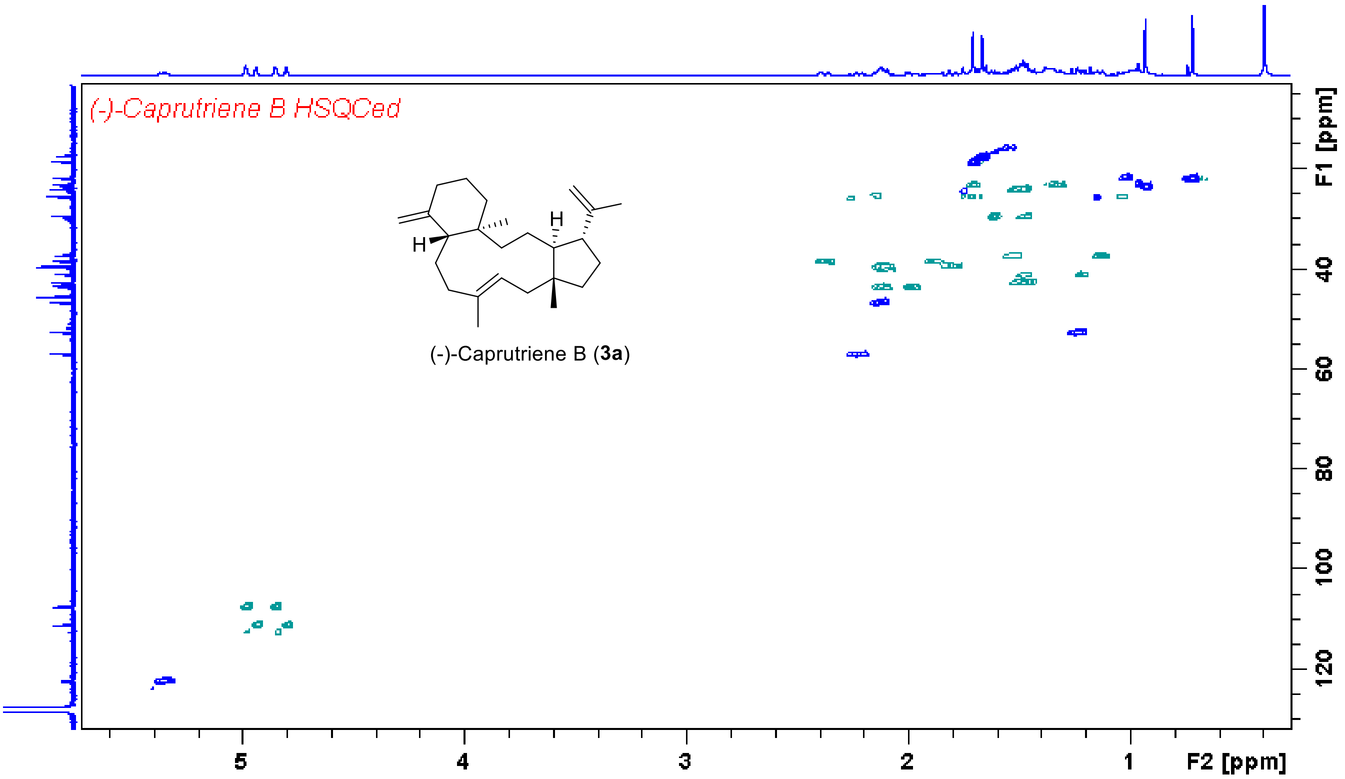

Figure S22. HSQC NMR (400 MHz, benzene-d_6) spectrum of (-)-caprutriene B (**3a**)

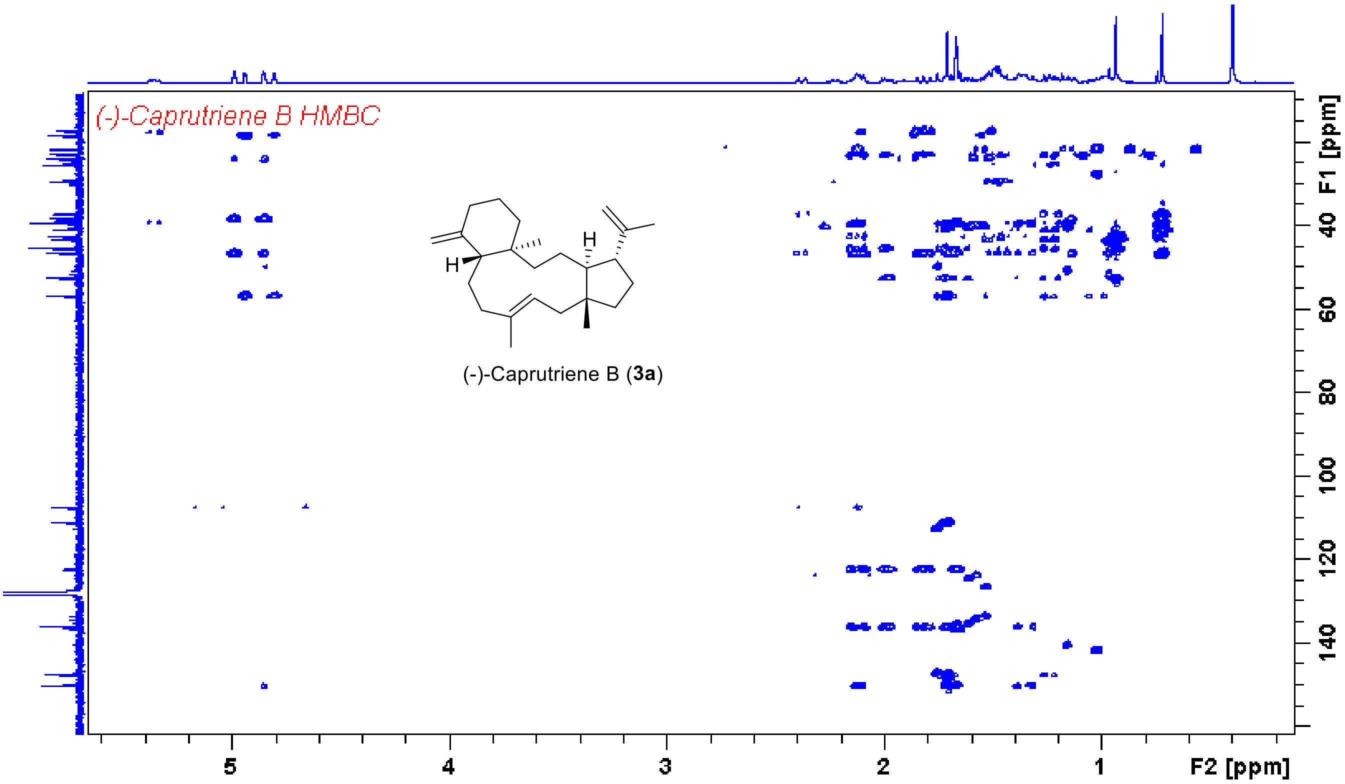

Figure S23. HMBC NMR (400 MHz, benzene-d$_6$) spectrum of (-)-caprutriene B (**3a**)

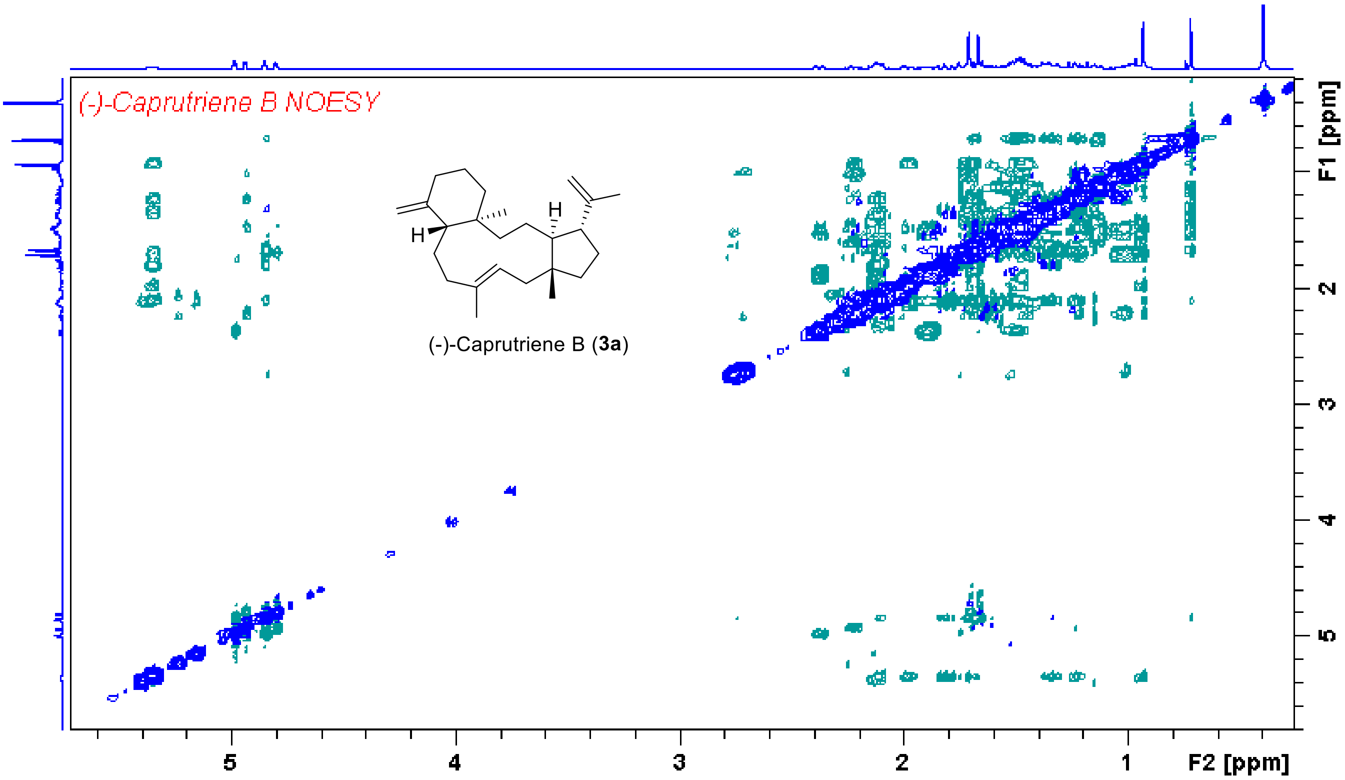

Figure S24. ^{1}H NMR (400 MHz, benzene-d$_6$) spectrum of (-)-caprutriene B (**3a**)

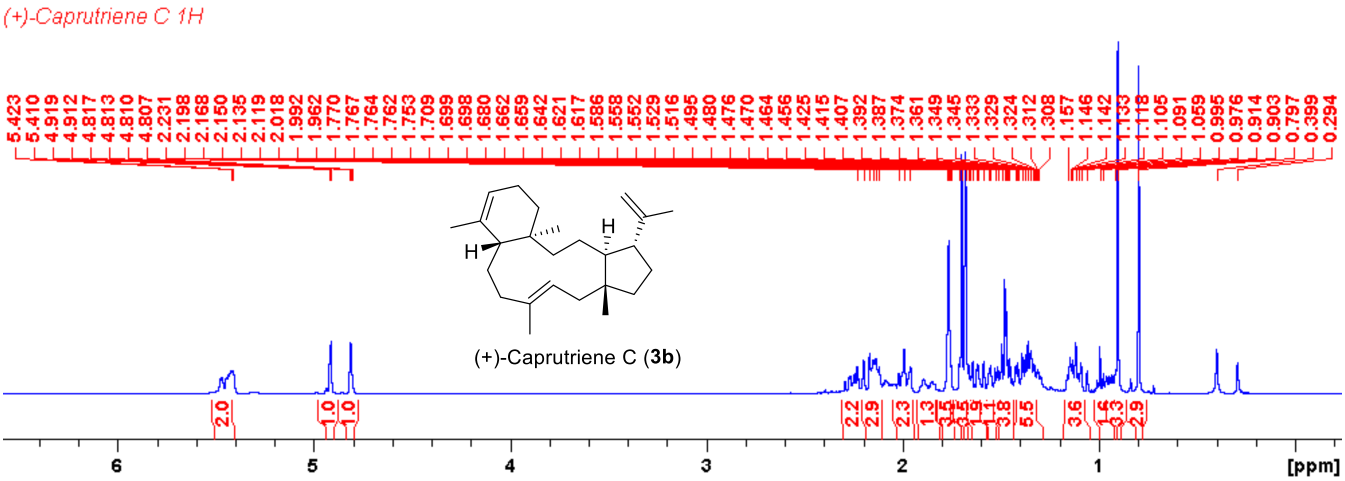

Figure S25. ^{1}H NMR (400 MHz, benzene-d$_6$) spectrum of (+)-caprutriene C (**3b**)

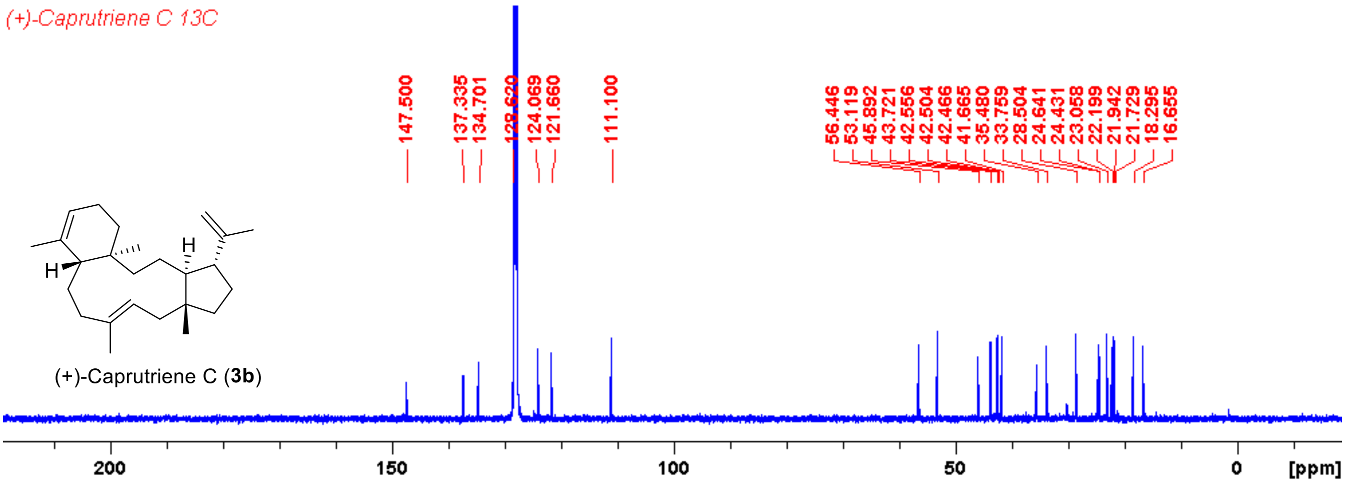

Figure S26. ^{13}C NMR (100 MHz, benzene-d$_6$) spectrum of (+)-caprutriene C (**3b**)

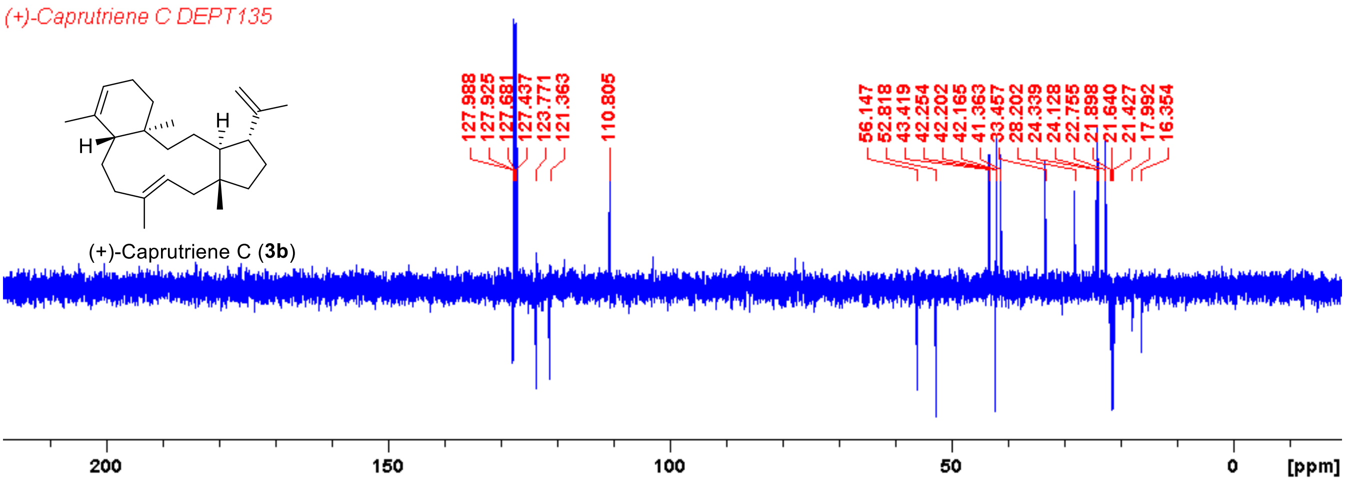

Figure S27. DEPT135 NMR (100 MHz, benzene-d$_6$) spectrum of (+)-caprutriene C (**3b**)

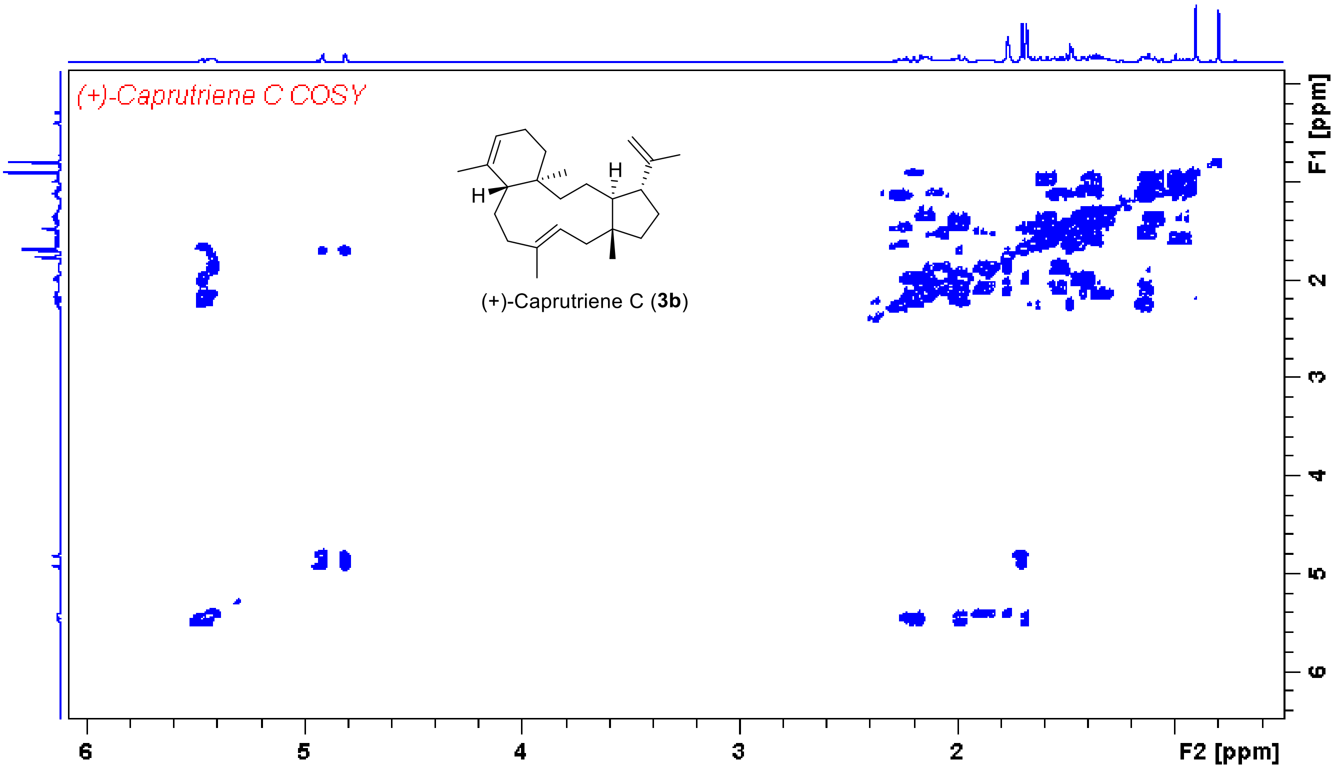

Figure S28. COSY NMR (400 MHz, benzene-d_6) spectrum of (+)-caprutriene C (**3b**)

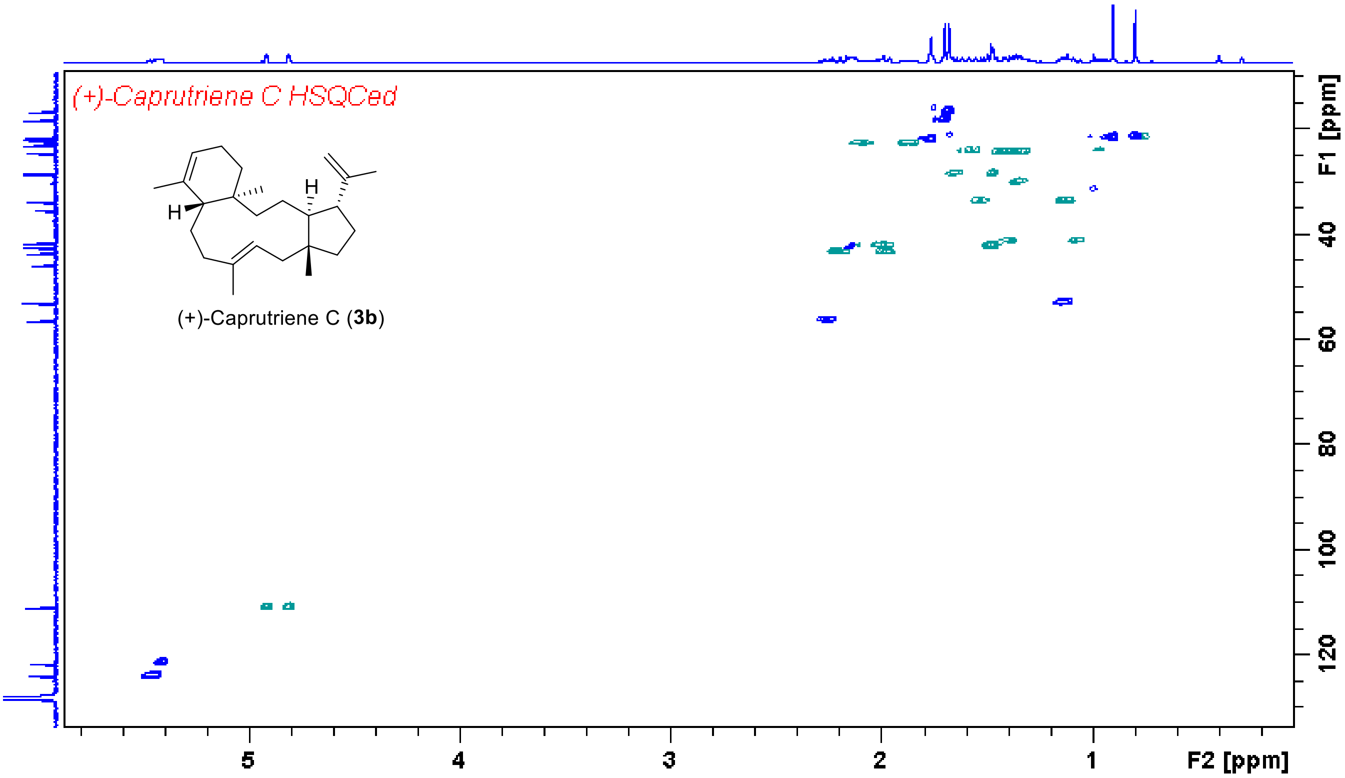

Figure S29. HSQC NMR (400 MHz, benzene-d_6) spectrum of (+)-caprutriene C (**3b**)

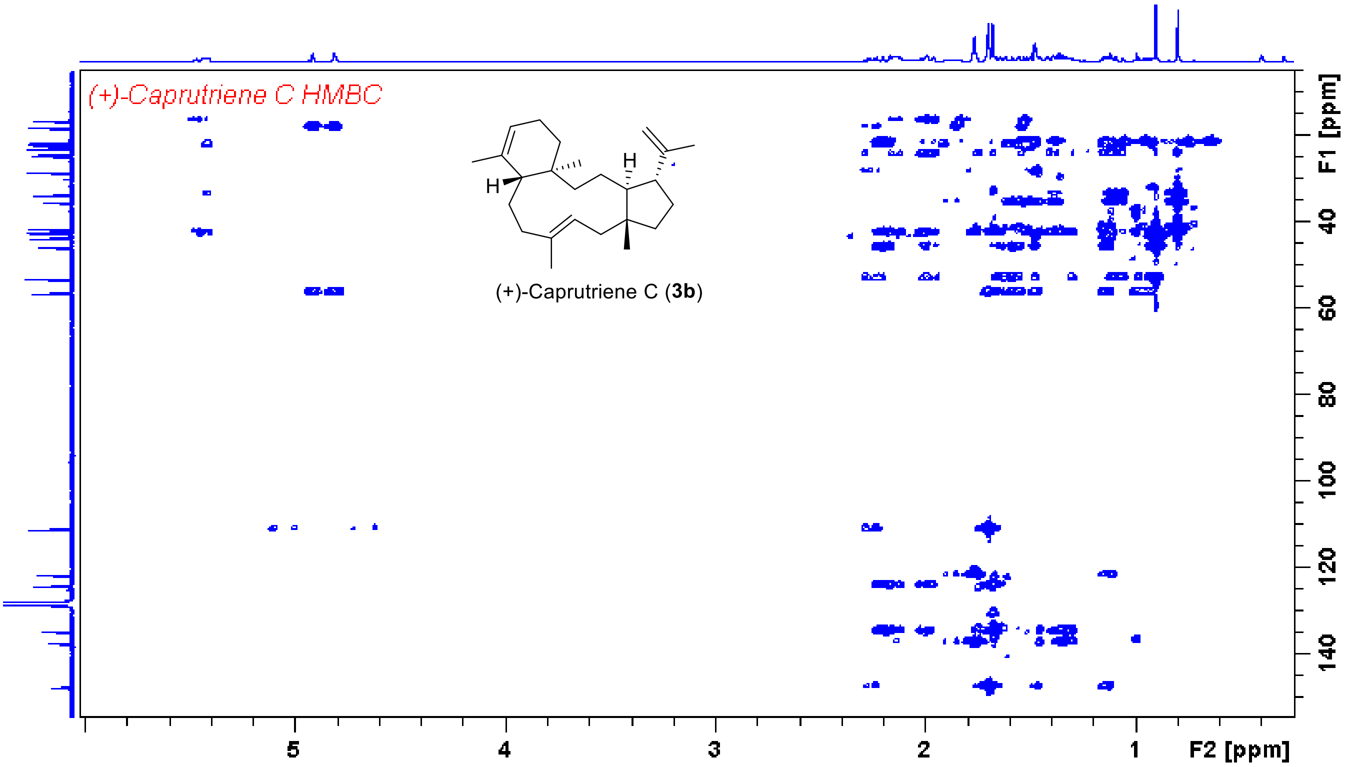

Figure S30. HMBC NMR (400 MHz, benzene-d6) spectrum of (+)-caprutriene C (**3b**)

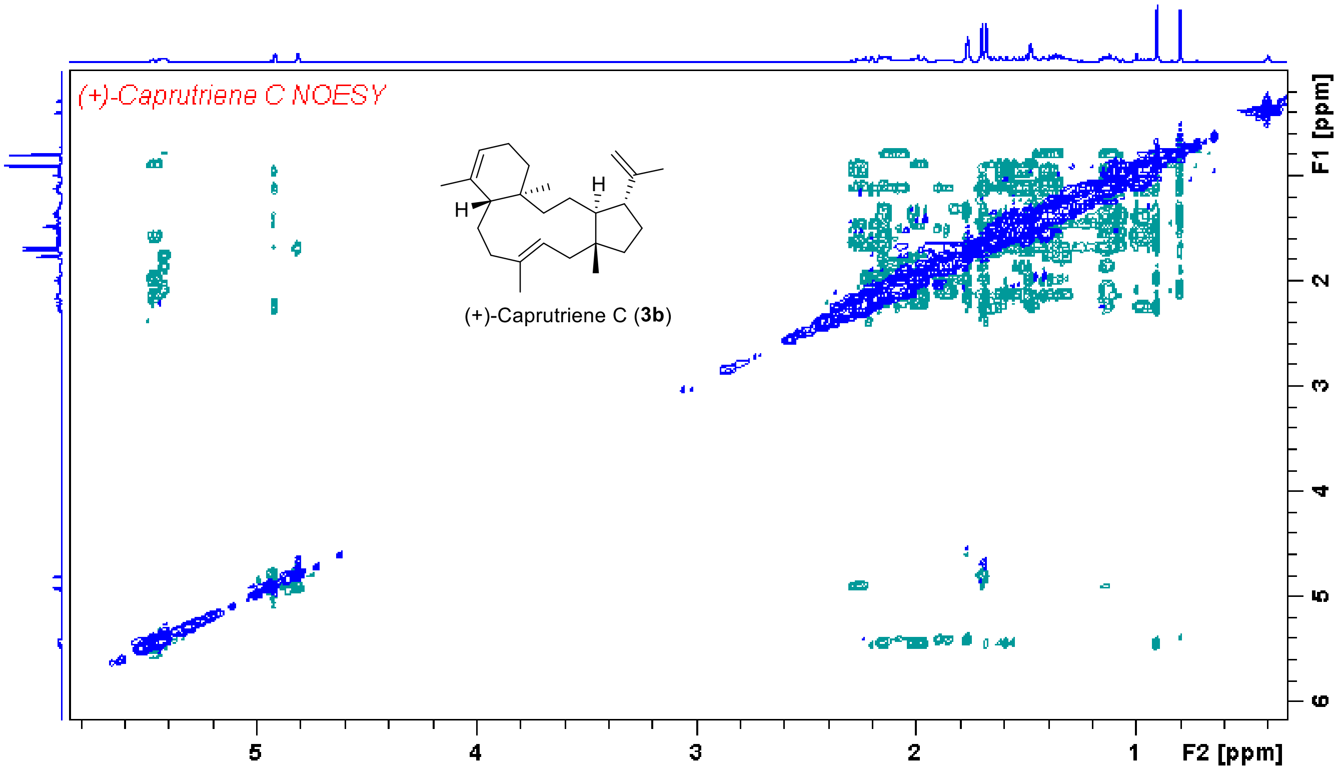

Figure S31. NOESY NMR (400 MHz, benzene-d6) spectrum of (+)-caprutriene C (**3b**)

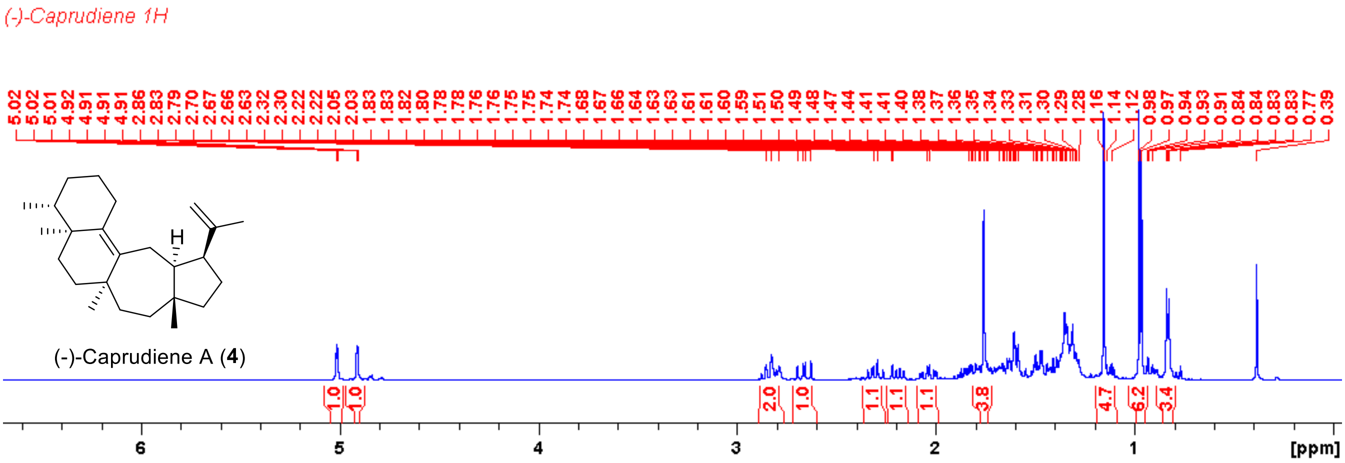

Figure S32. ^{1}H NMR (400 MHz, benzene-d$_6$) spectrum of (-)-caprudiene A (**4**)

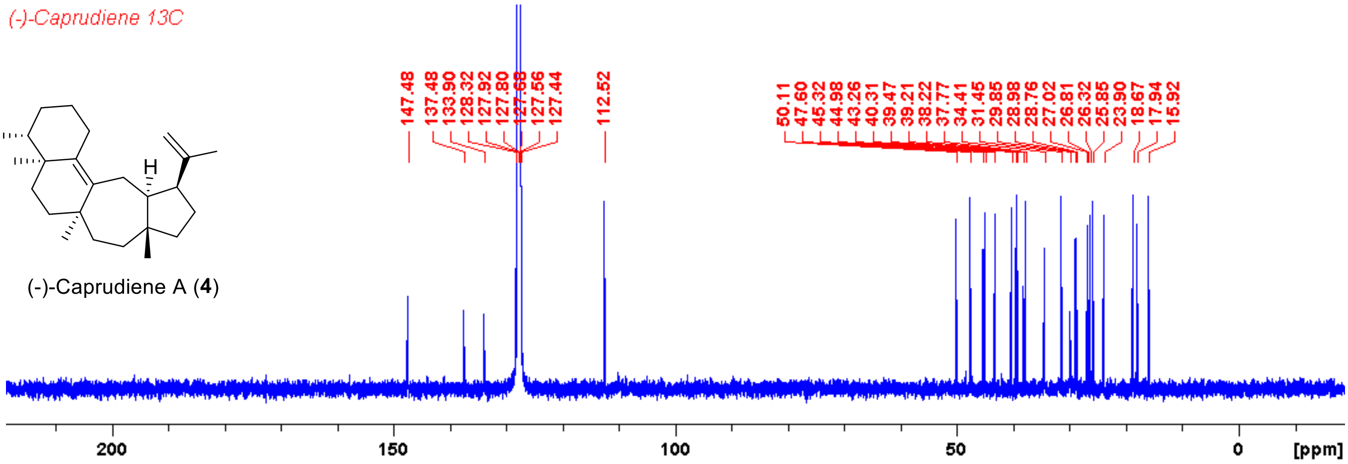

Figure S33. ^{13}C NMR (100 MHz, benzene-d$_6$) spectrum of (-)-caprudiene A (**4**)

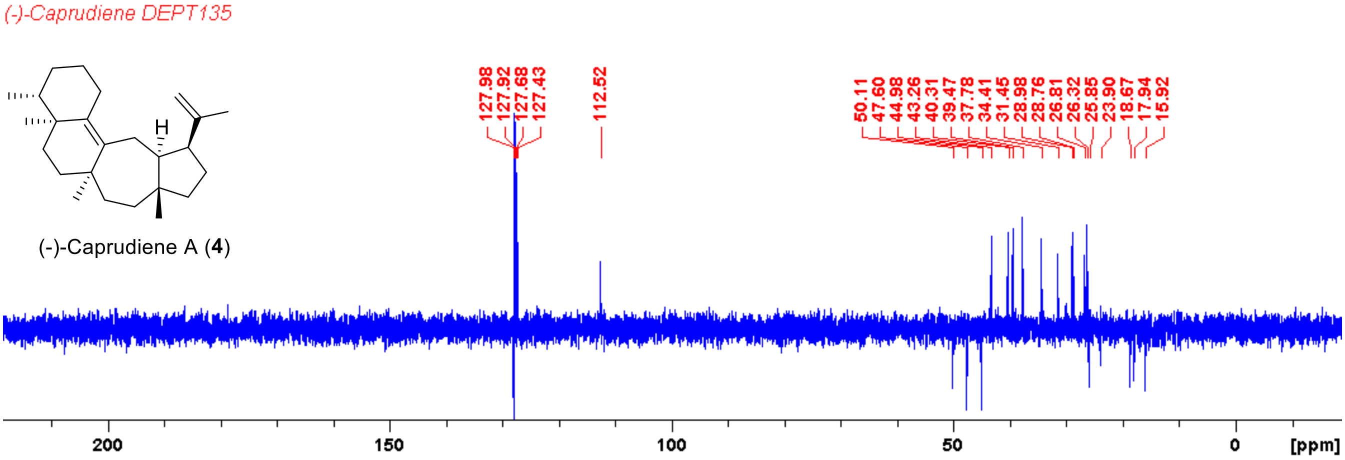

Figure S34. DEPT135 NMR (100 MHz, benzene-d$_6$) spectrum of (-)-caprudiene A (**4**)

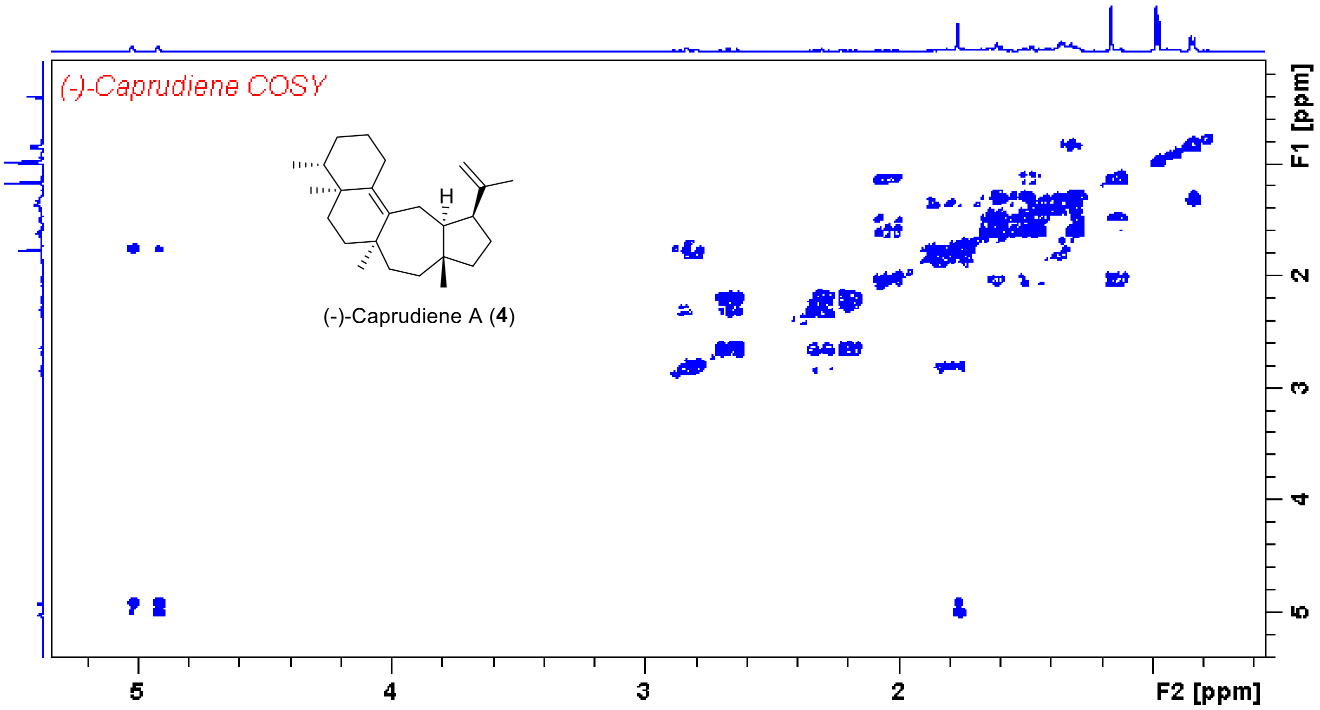

Figure S35. COSY NMR (400 MHz, benzene-d$_6$) spectrum of (-)-caprudiene A (**4**)

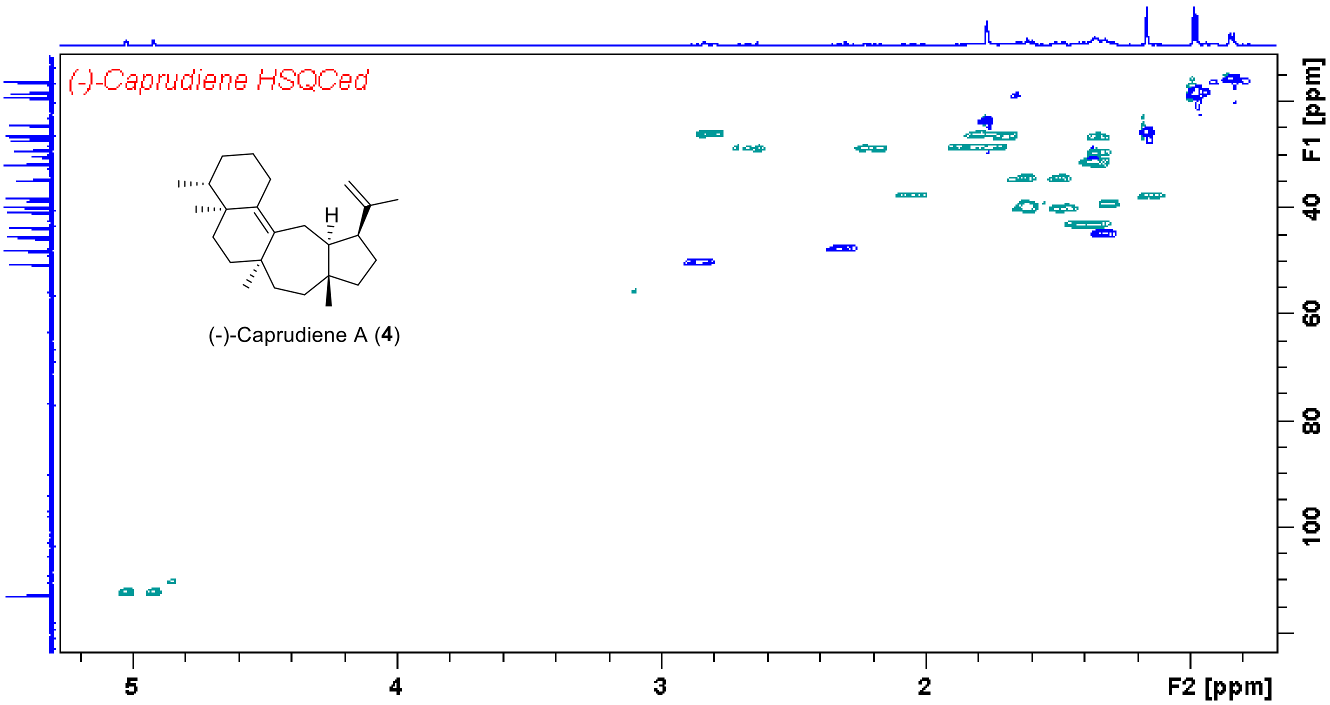

Figure S36. HSQC NMR (400 MHz, benzene-d$_6$) spectrum of (-)-caprudiene A (**4**)

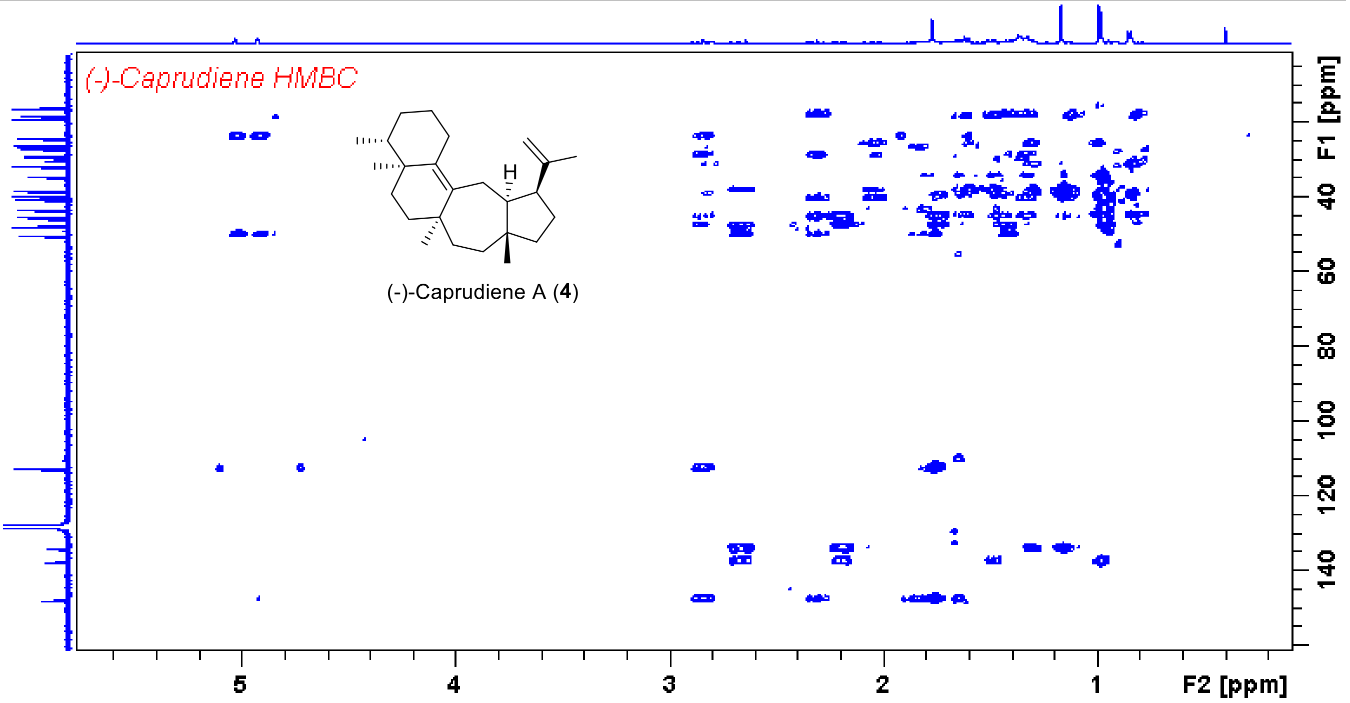

Figure S37. HMBC NMR (400 MHz, benzene-d_6) spectrum of (-)-caprudiene A (**4**)

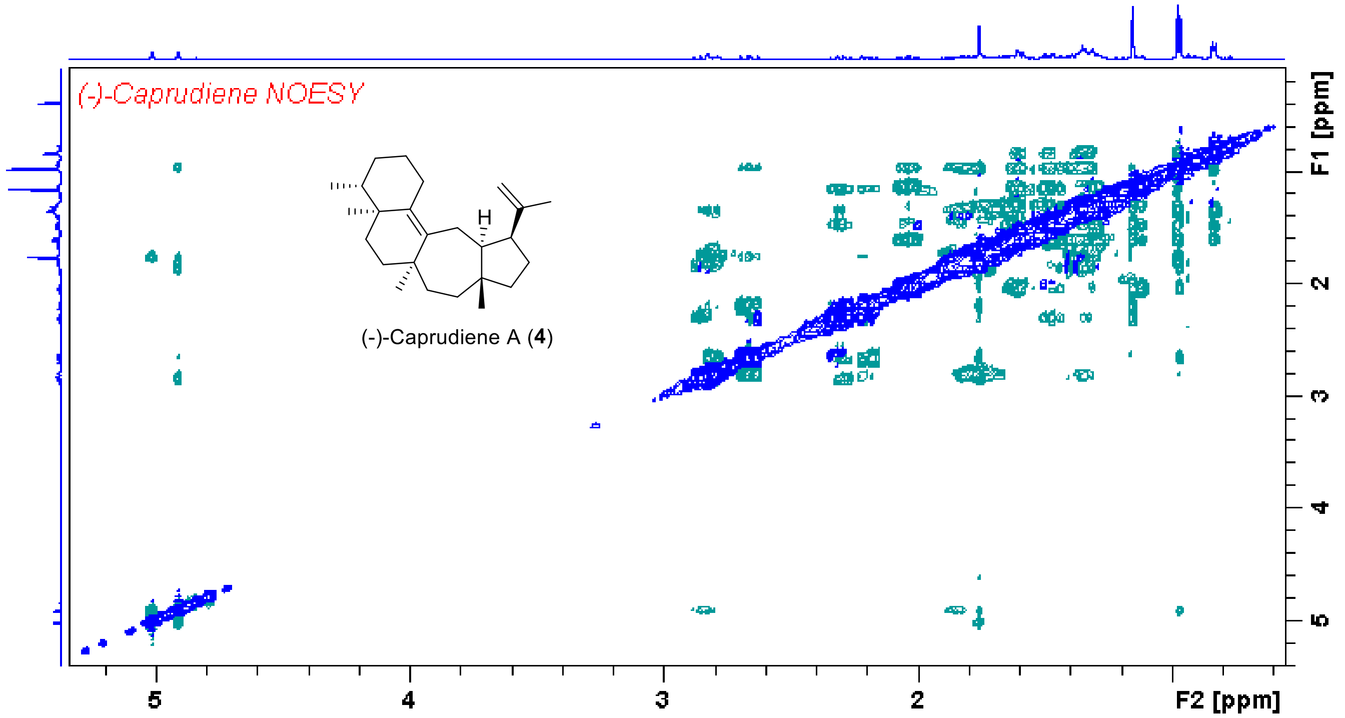

Figure S38. NOESY NMR (400 MHz, benzene-d_6) spectrum of (-)-caprudiene A (**4**)

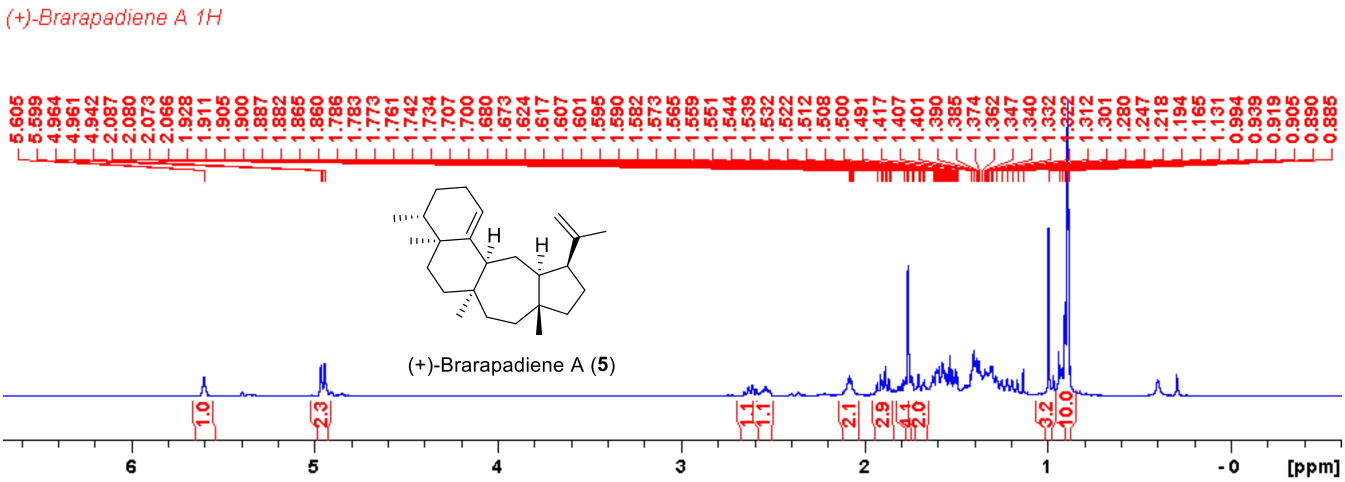

Figure S39. ^{1}H NMR (400 MHz, benzene-d_6) spectrum of (+)-brarapadiene A (**5**)

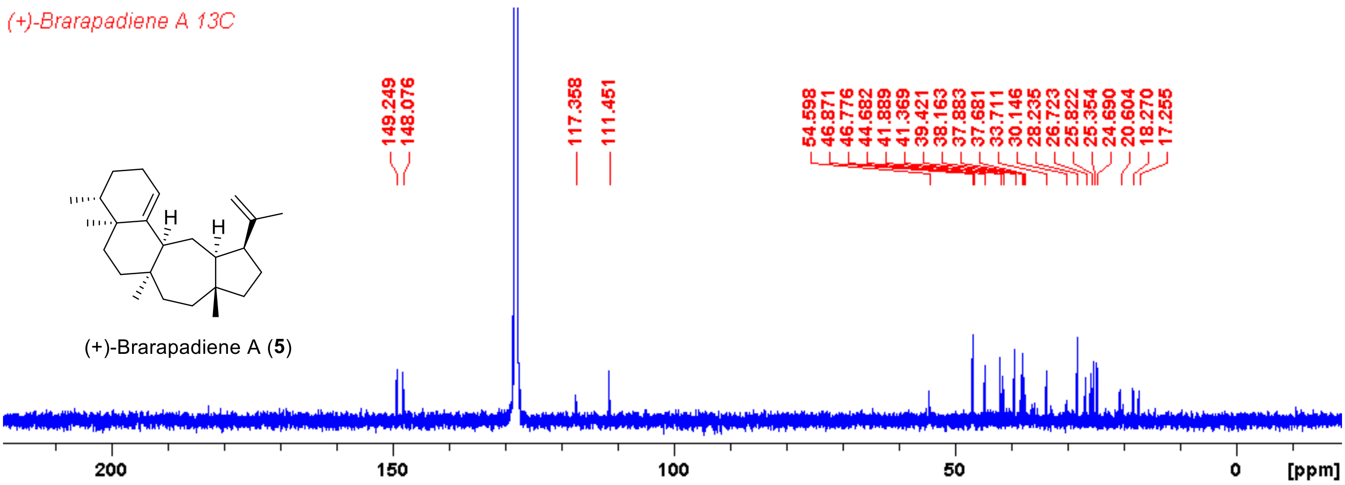

Figure S40. ^{13}C NMR (100 MHz, benzene-d_6) spectrum of (+)-brarapadiene A (**5**)

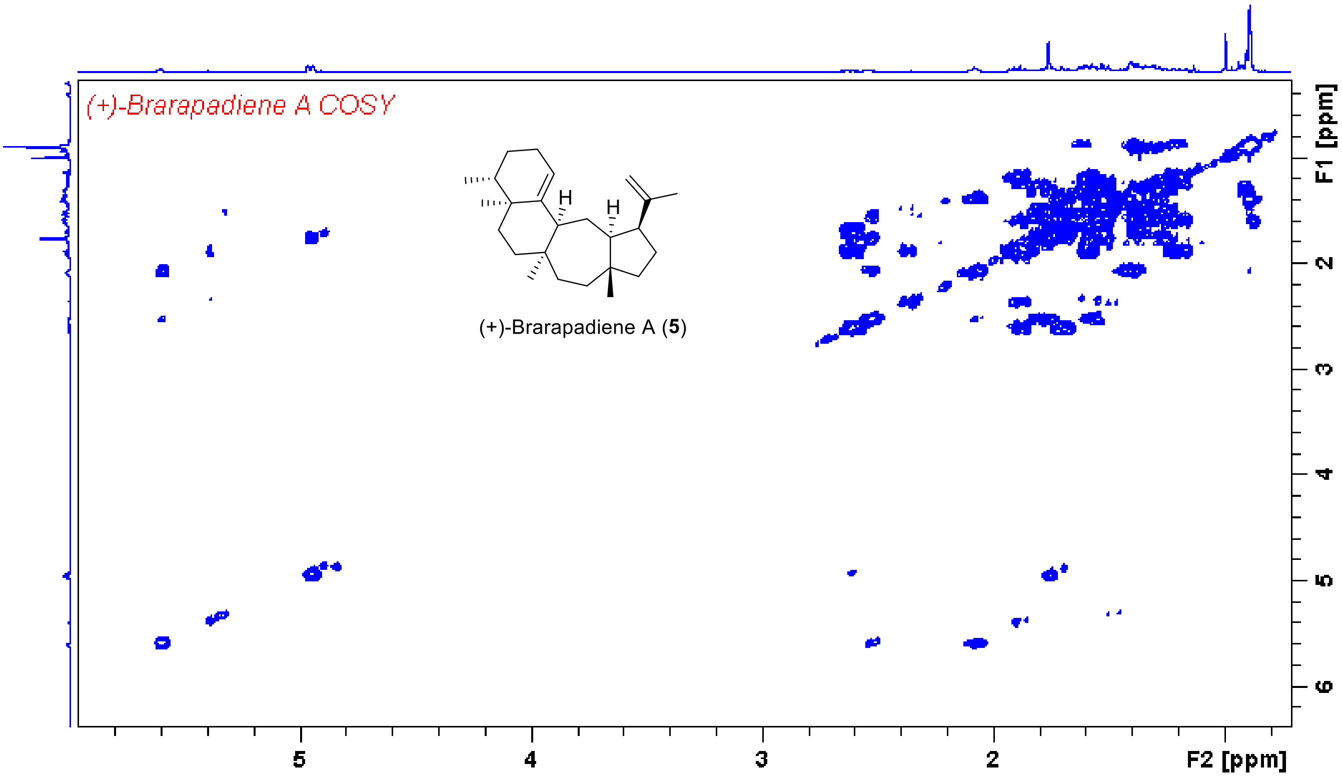

Figure S41. COSY NMR (400 MHz, benzene-d6) spectrum of (+)-brarapadiene A (**5**)

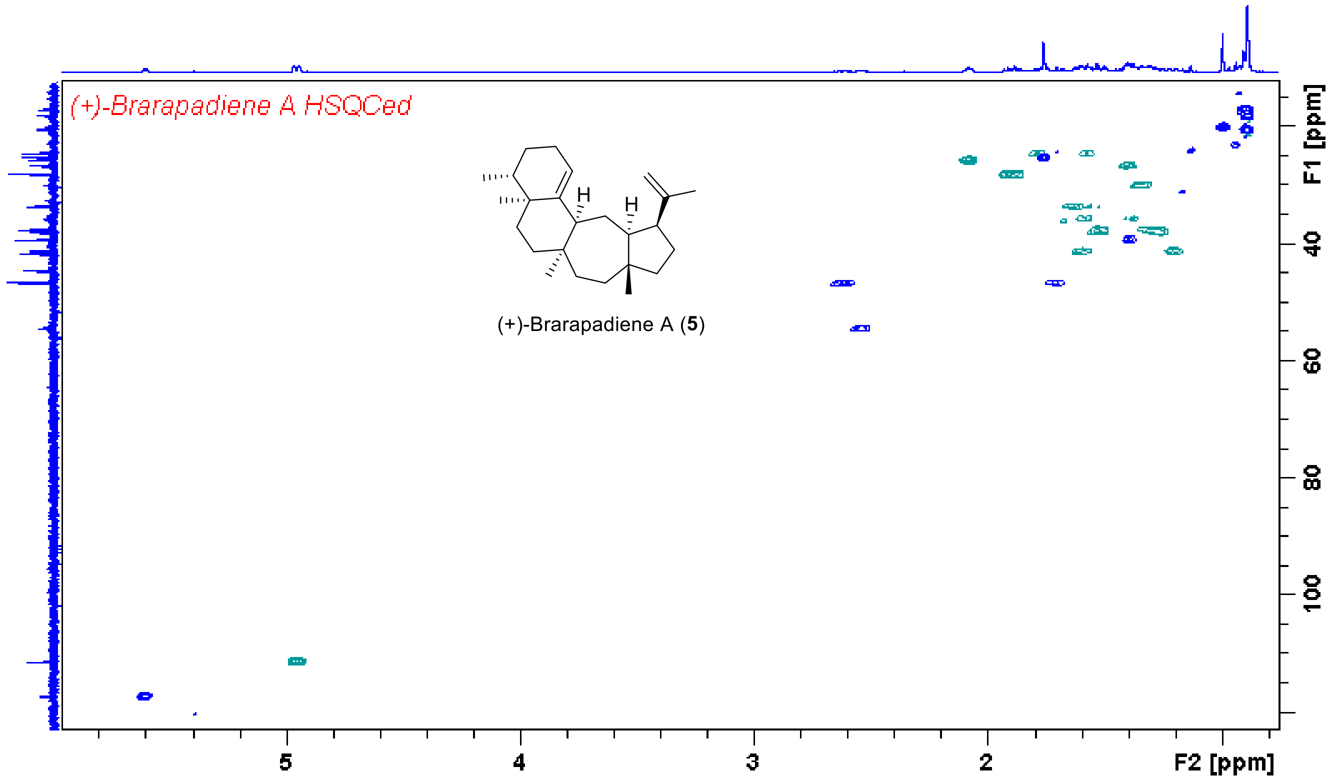

Figure S42. HSQC NMR (400 MHz, benzene-d6) spectrum of (+)-brarapadiene A (**5**)

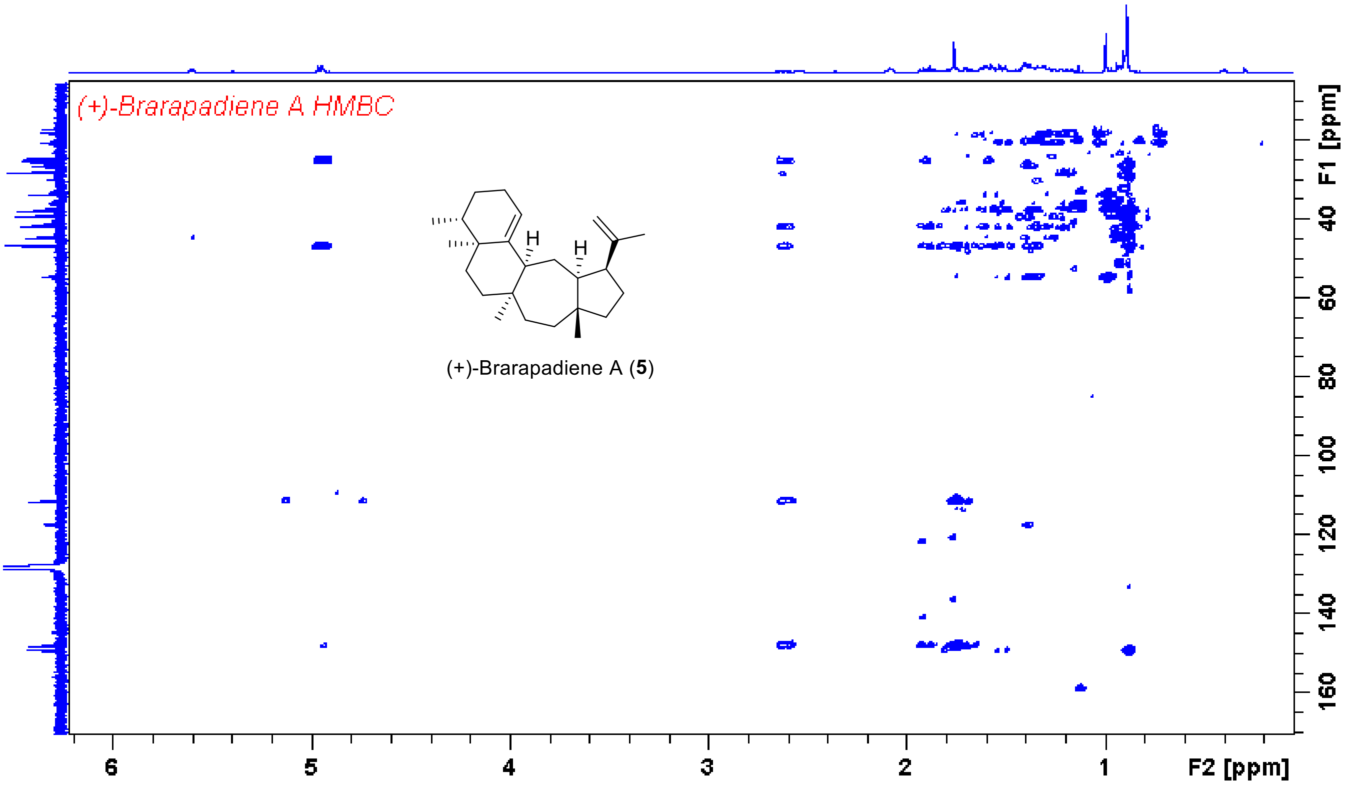

Figure S43. HMBC NMR (400 MHz, benzene-d_6) spectrum of (+)-brarapadiene A (**5**)

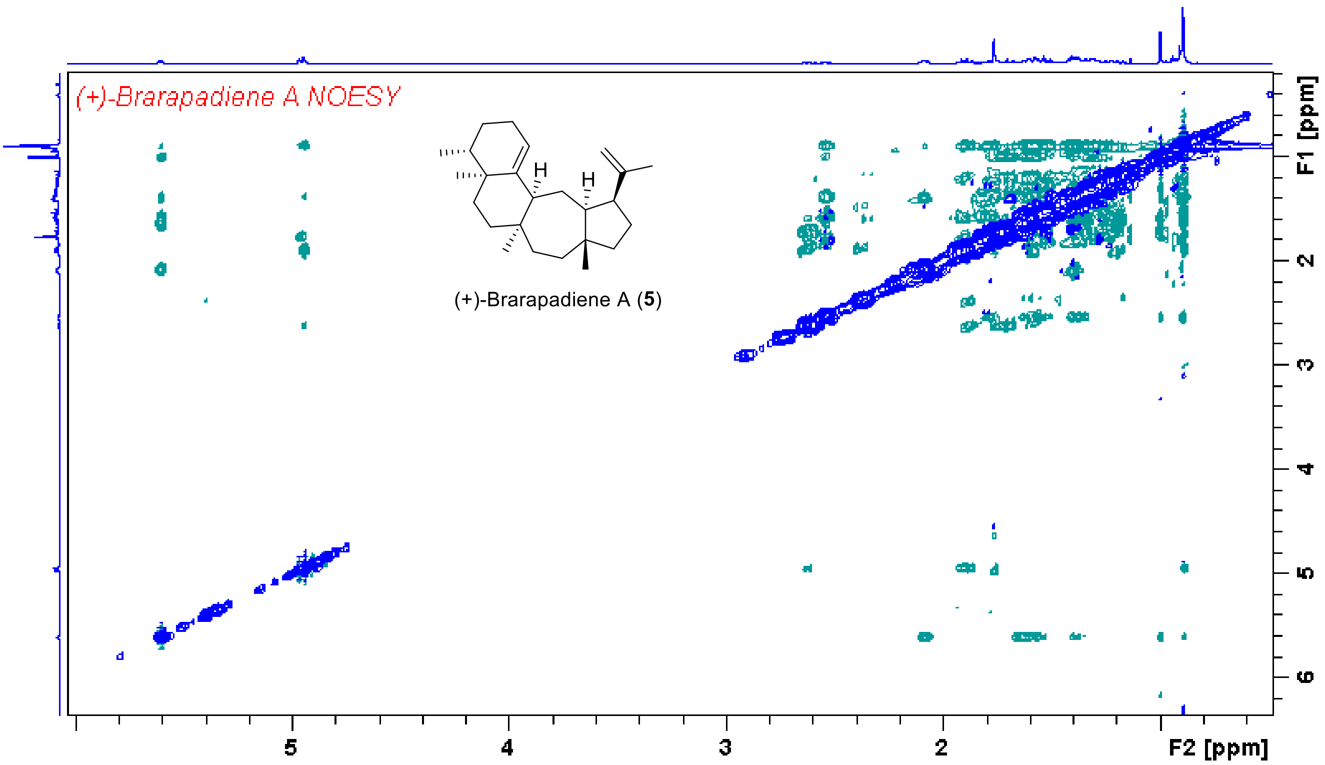

Figure S44. NOESY NMR (400 MHz, benzene-d_6) spectrum of (+)-brarapadiene A (**5**)

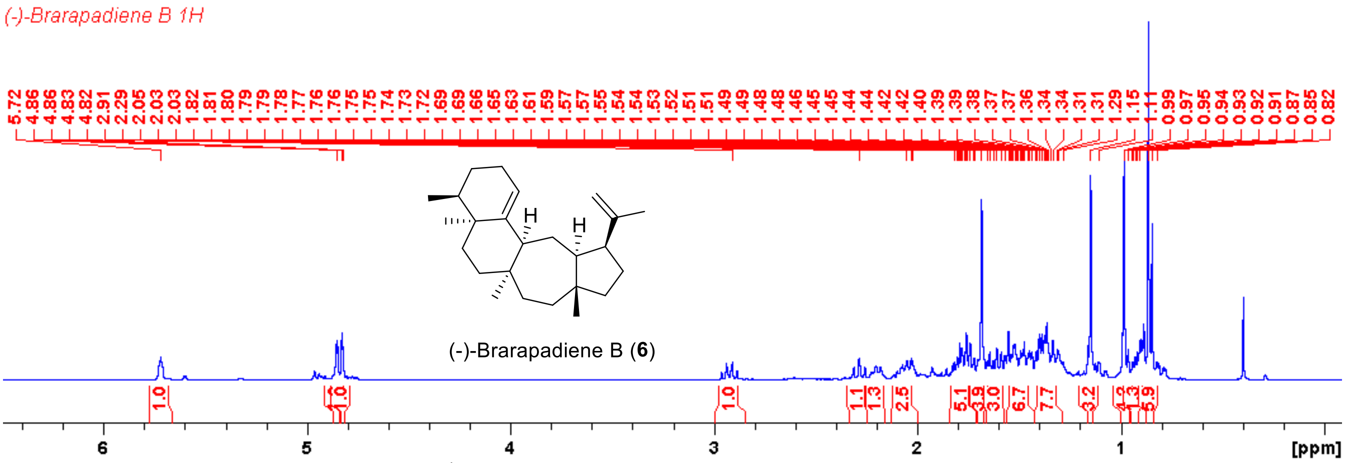

Figure S45. ^{1}H NMR (400 MHz, benzene-d_6) spectrum of (-)-brarapadiene B (**6**)

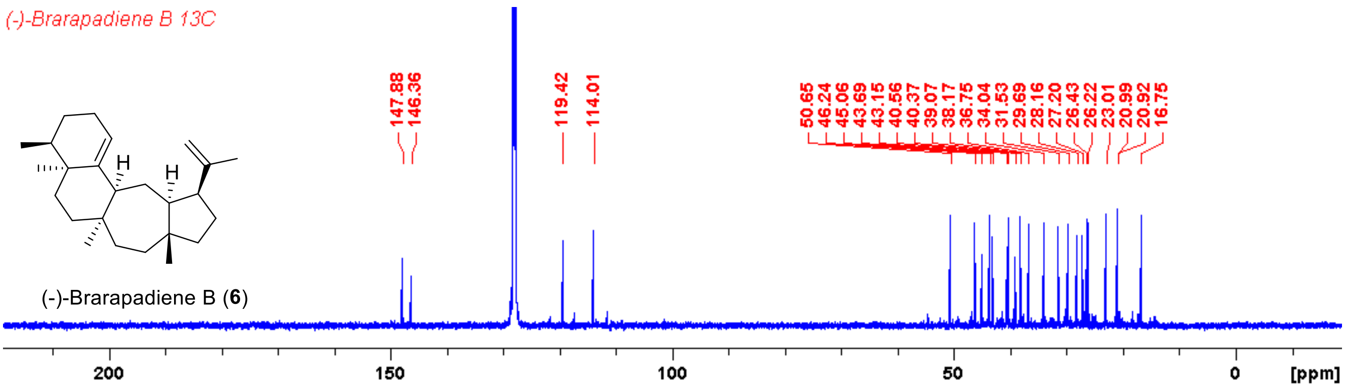

Figure S46. ^{13}C NMR (100 MHz, benzene-d_6) spectrum of (-)-brarapadiene B (**6**)

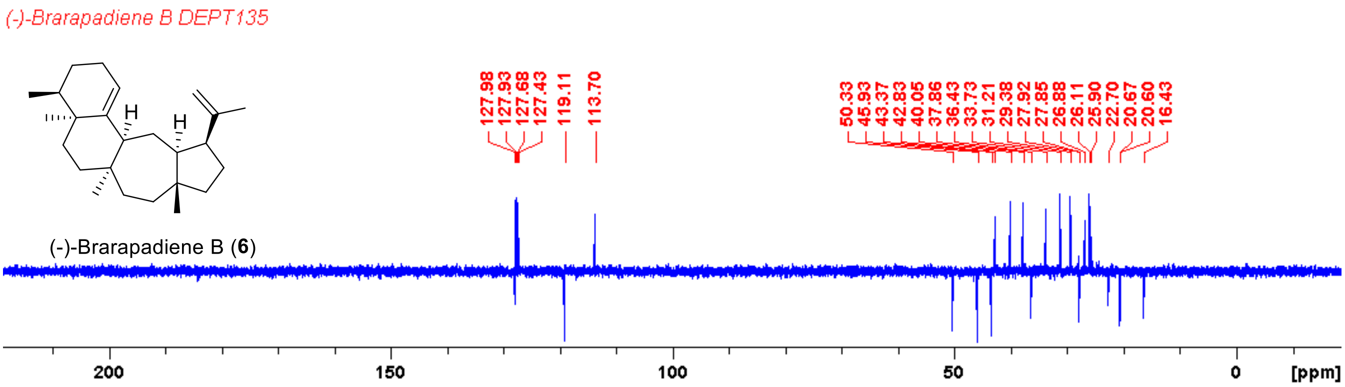

Figure S47. DEPT135 NMR (100 MHz, benzene-d_6) spectrum of (-)-brarapadiene B (**6**)

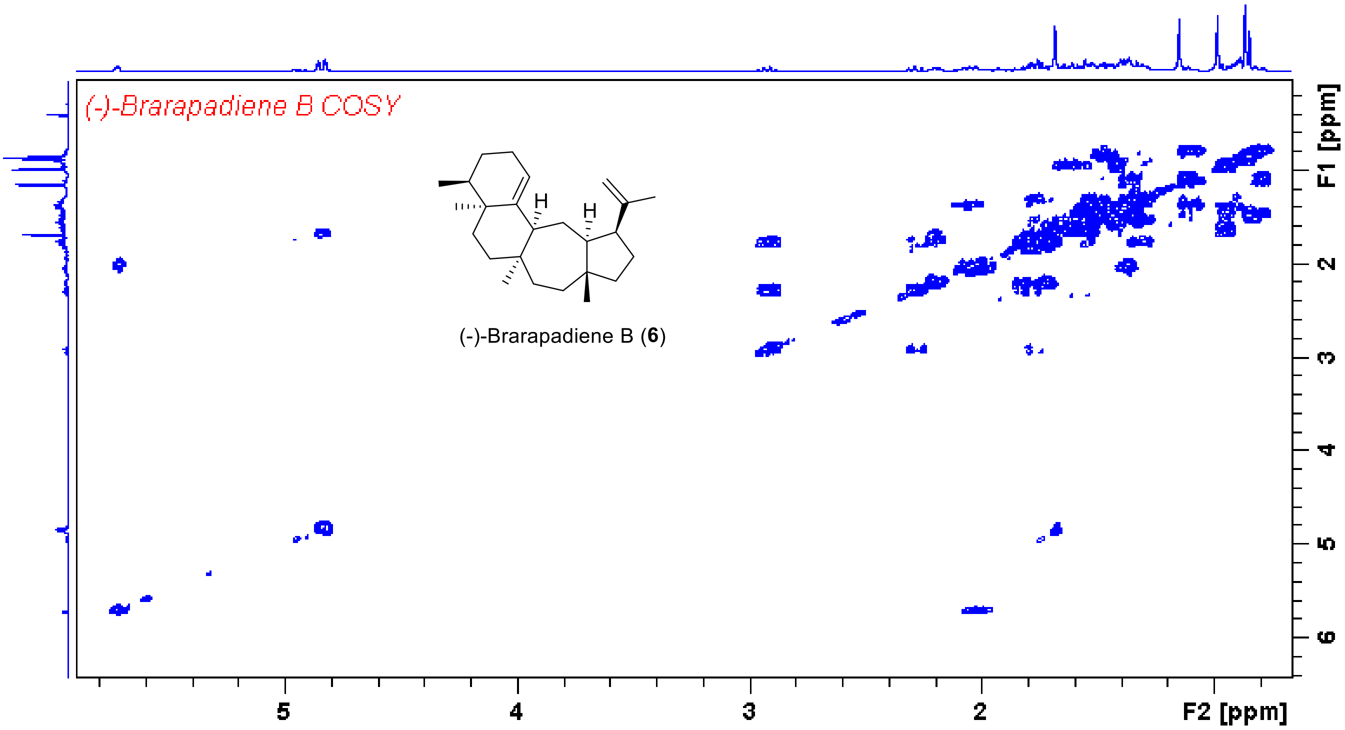

Figure S48. COSY NMR (400 MHz, benzene-d6) spectrum of (-)-brarapadiene B (**6**)

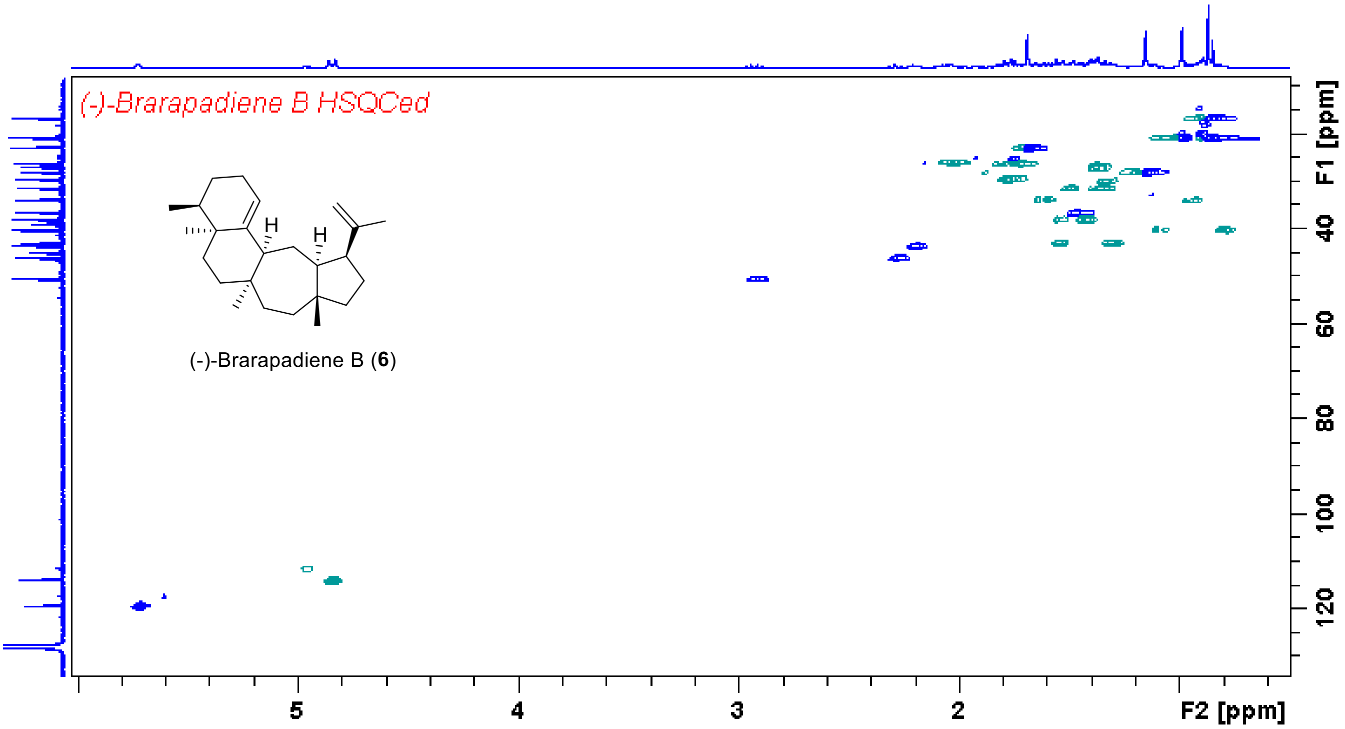

Figure S49. HSQC NMR (400 MHz, benzene-d6) spectrum of (-)-brarapadiene B (**6**)

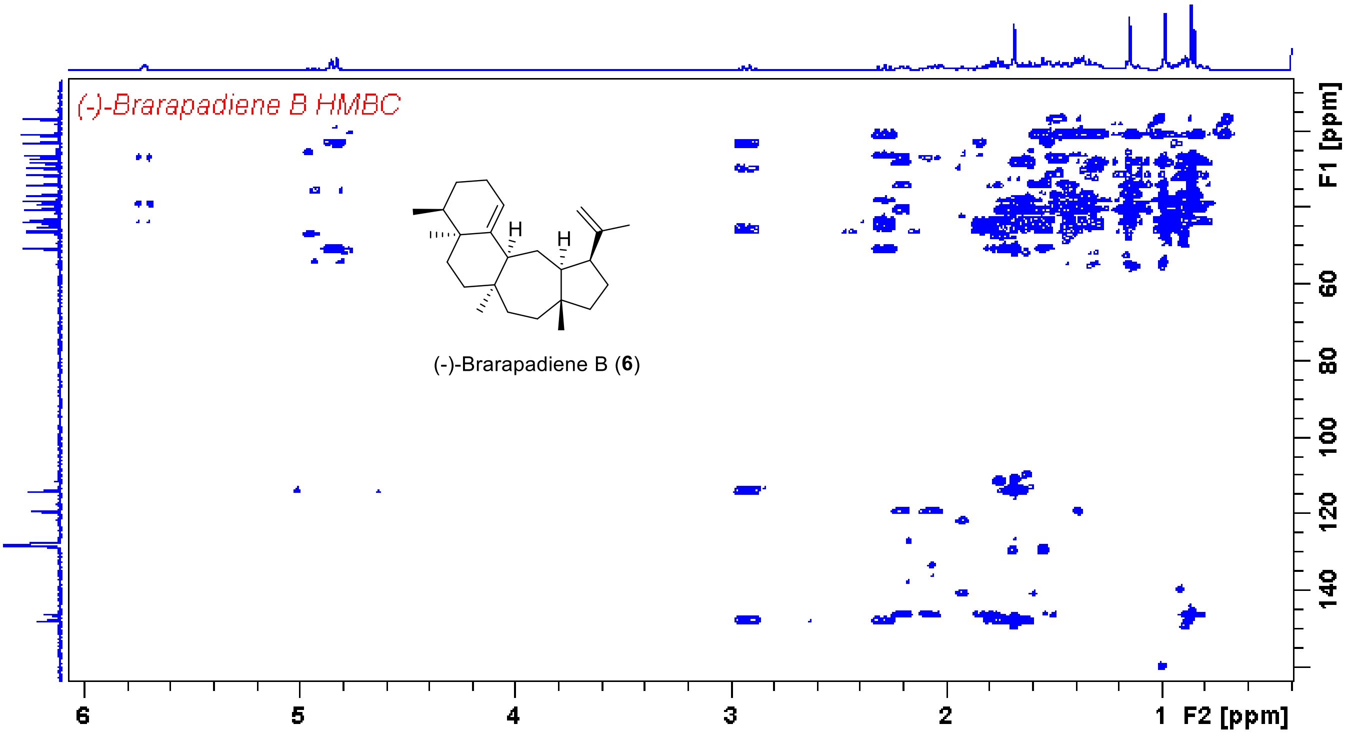

Figure S50. HMBC NMR (400 MHz, benzene-d_6) spectrum of (-)-brarapadiene B (**6**)

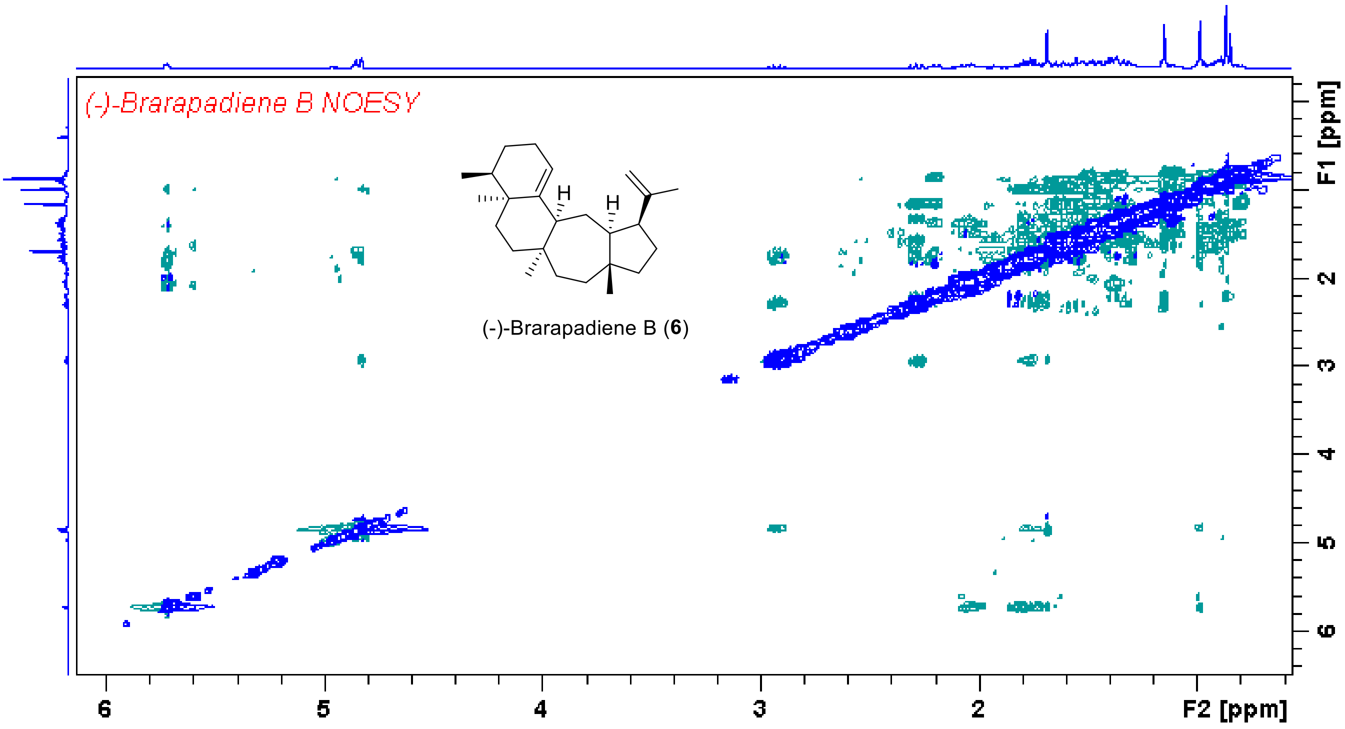

Figure S51. NOESY NMR (400 MHz, benzene-d_6) spectrum of (-)-brarapadiene B (**6**)

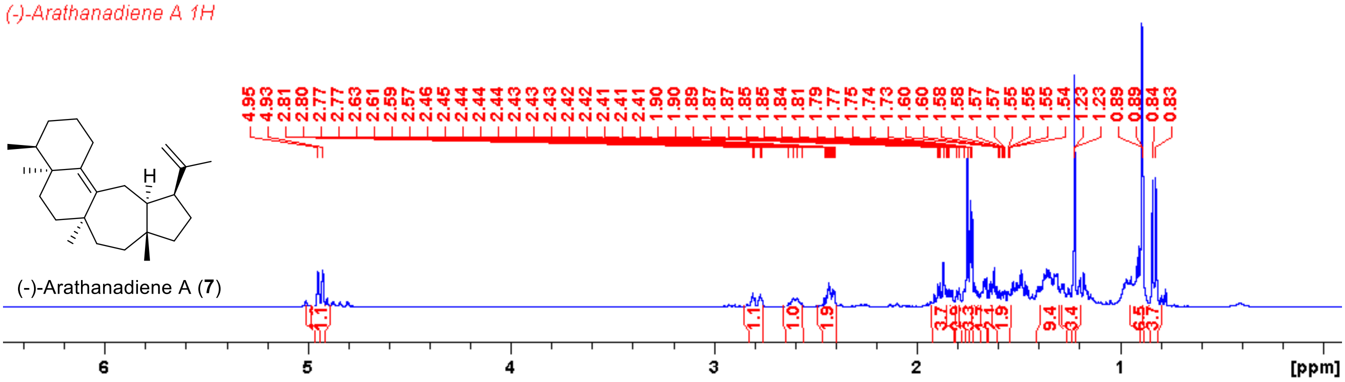

Figure S52. ^{1}H NMR (400 MHz, benzene-d$_6$) spectrum of (-)-arathanadiene A (**7**)

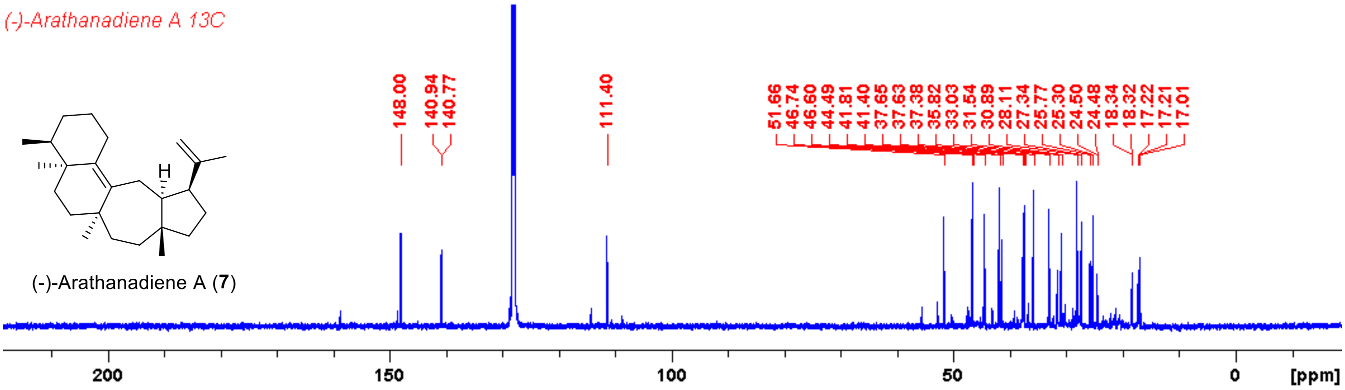

Figure S53. ^{13}C NMR (100 MHz, benzene-d$_6$) spectrum of (-)-arathanadiene A (**7**)

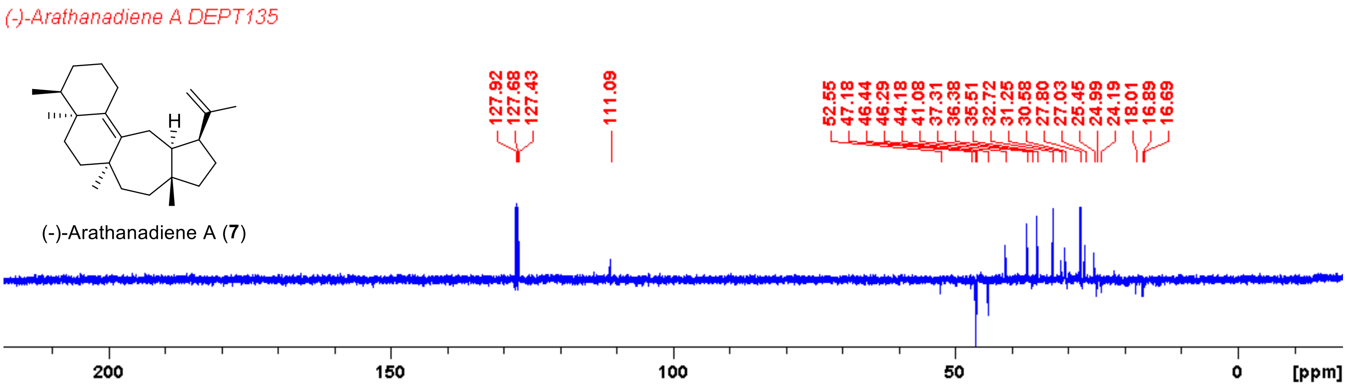

Figure S54. DEPT135 NMR (100 MHz, benzene-d$_6$) spectrum of (-)-arathanadiene A (**7**)

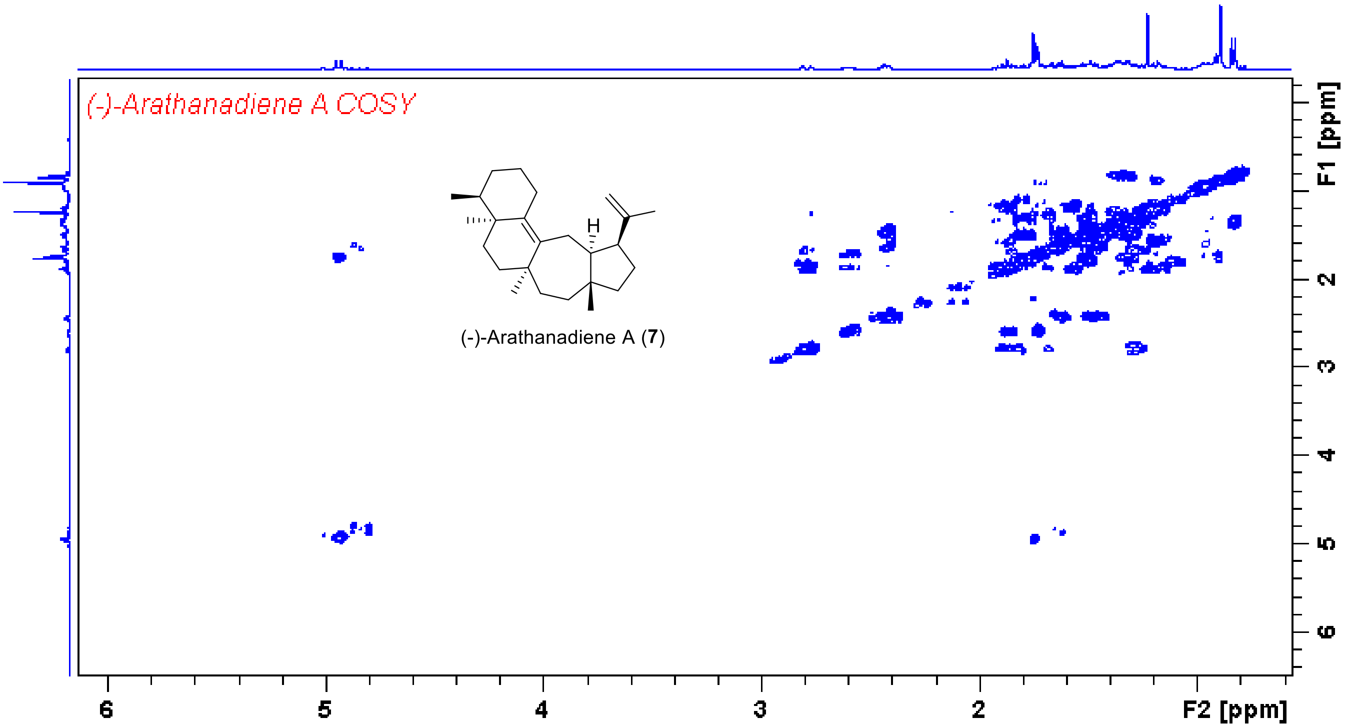

Figure S55. COSY NMR (400 MHz, benzene-d6) spectrum of (-)-arathanadiene A (**7**)

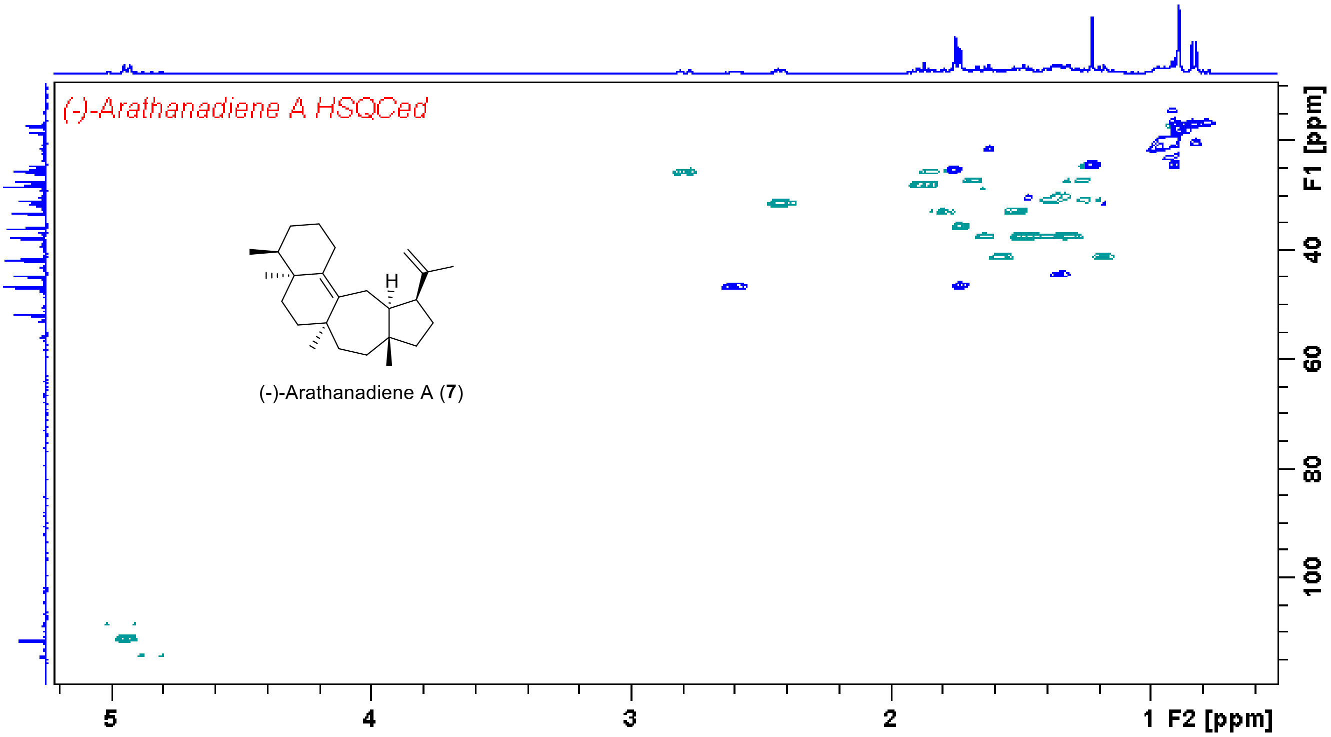

Figure S56. HSQC NMR (400 MHz, benzene-d6) spectrum of (-)-arathanadiene A (**7**)

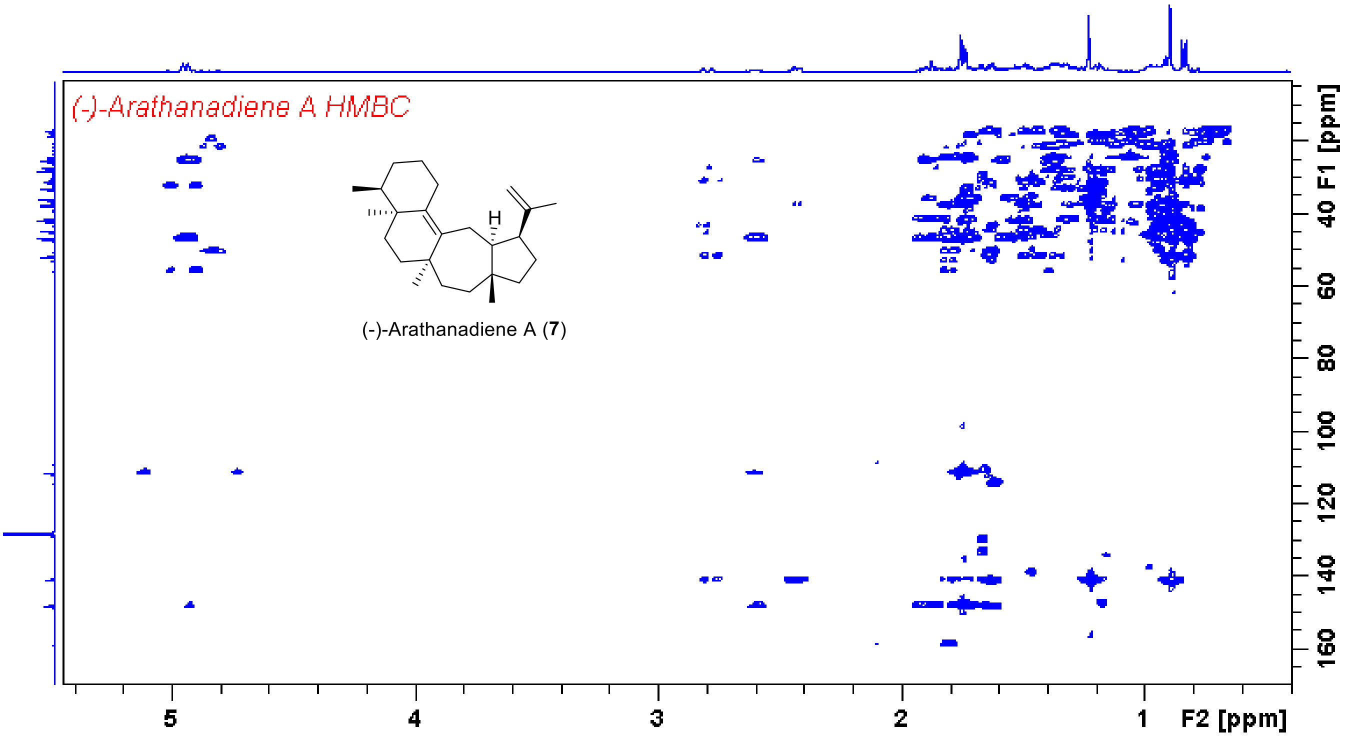

Figure S57. HMBC NMR (400 MHz, benzene-d6) spectrum of (-)-arathanadiene A (**7**)

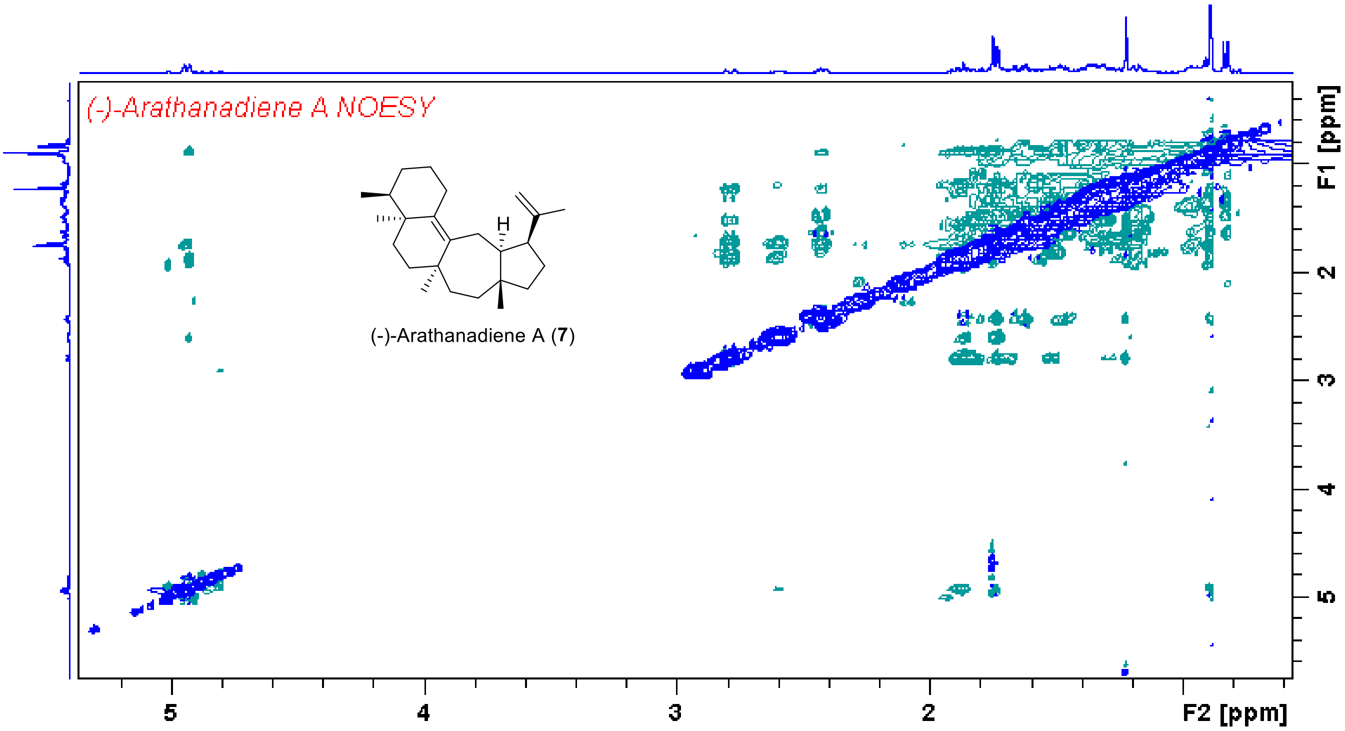

Figure S58. NOESY NMR (400 MHz, benzene-d6) spectrum of (-)-arathanadiene A (**7**)

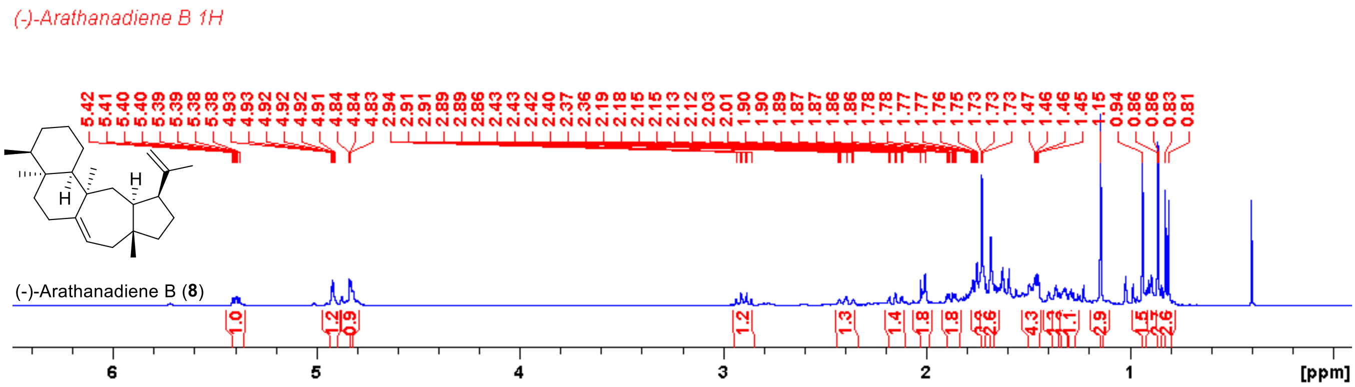

Figure S59. ^{1}H NMR (400 MHz, benzene-d6) spectrum of (-)-arathanadiene B (**8**)

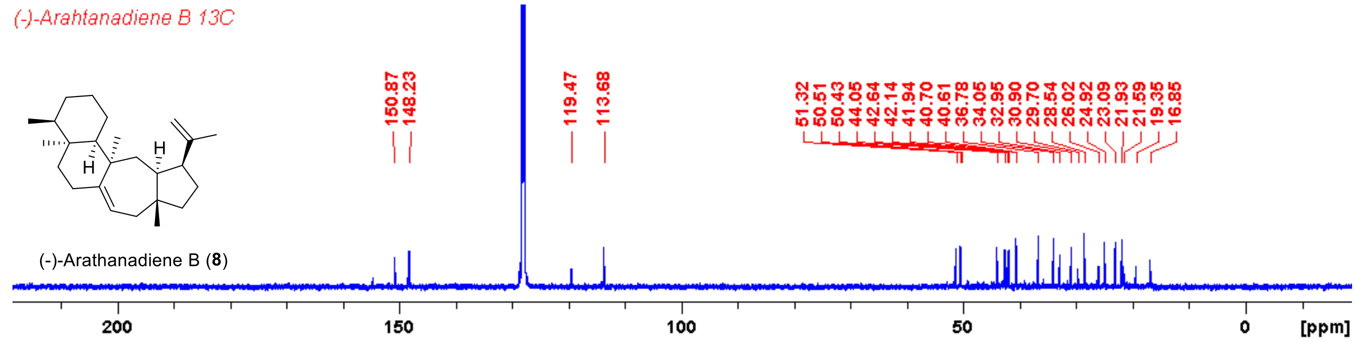

Figure S60. ^{13}C NMR (100 MHz, benzene-d6) spectrum of (-)-arathanadiene B (**8**)

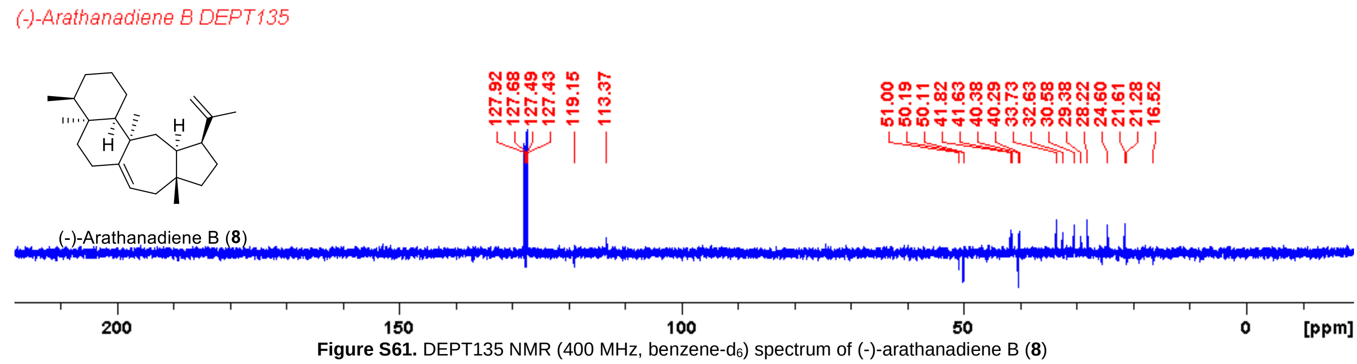

Figure S61. DEPT135 NMR (400 MHz, benzene-d6) spectrum of (-)-arathanadiene B (**8**)

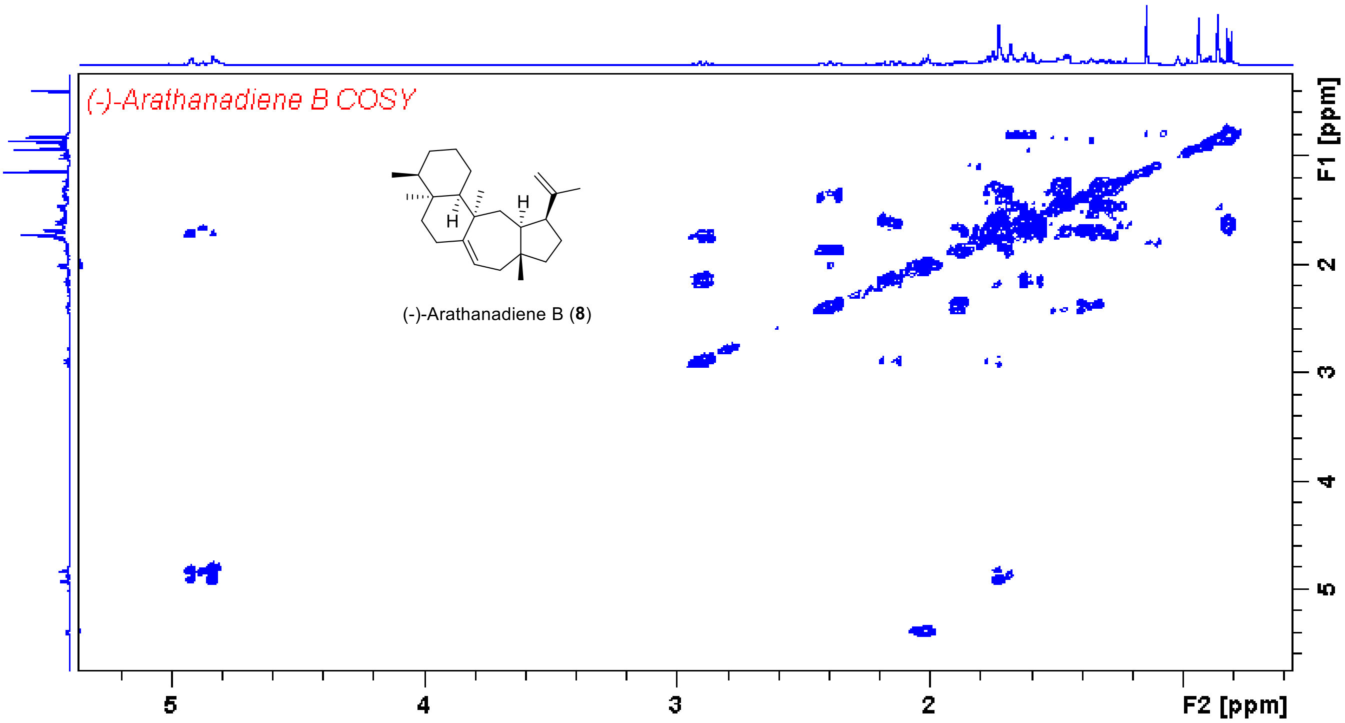

Figure S62. COSY NMR (400 MHz, benzene-d_6) spectrum of (-)-arathanadiene B (**8**)

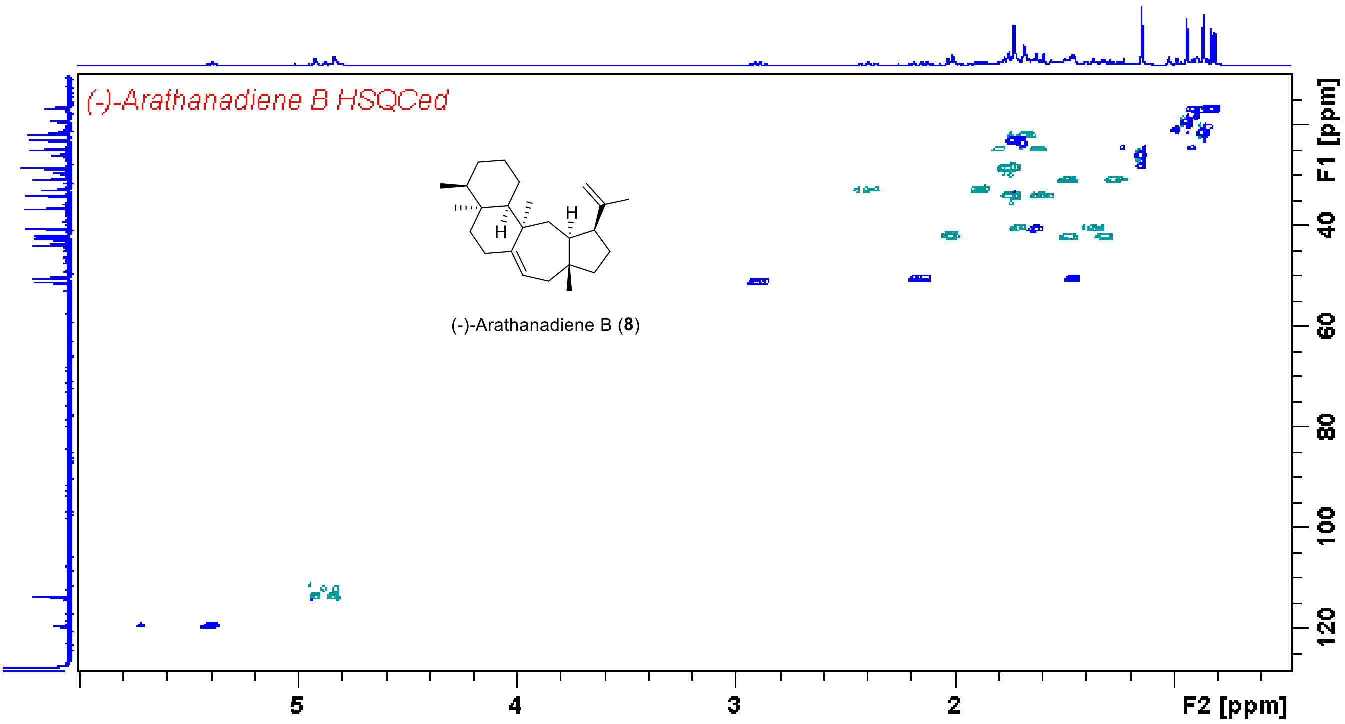

Figure S63. HSQC NMR (400 MHz, benzene-d_6) spectrum of (-)-arathanadiene B (**8**)

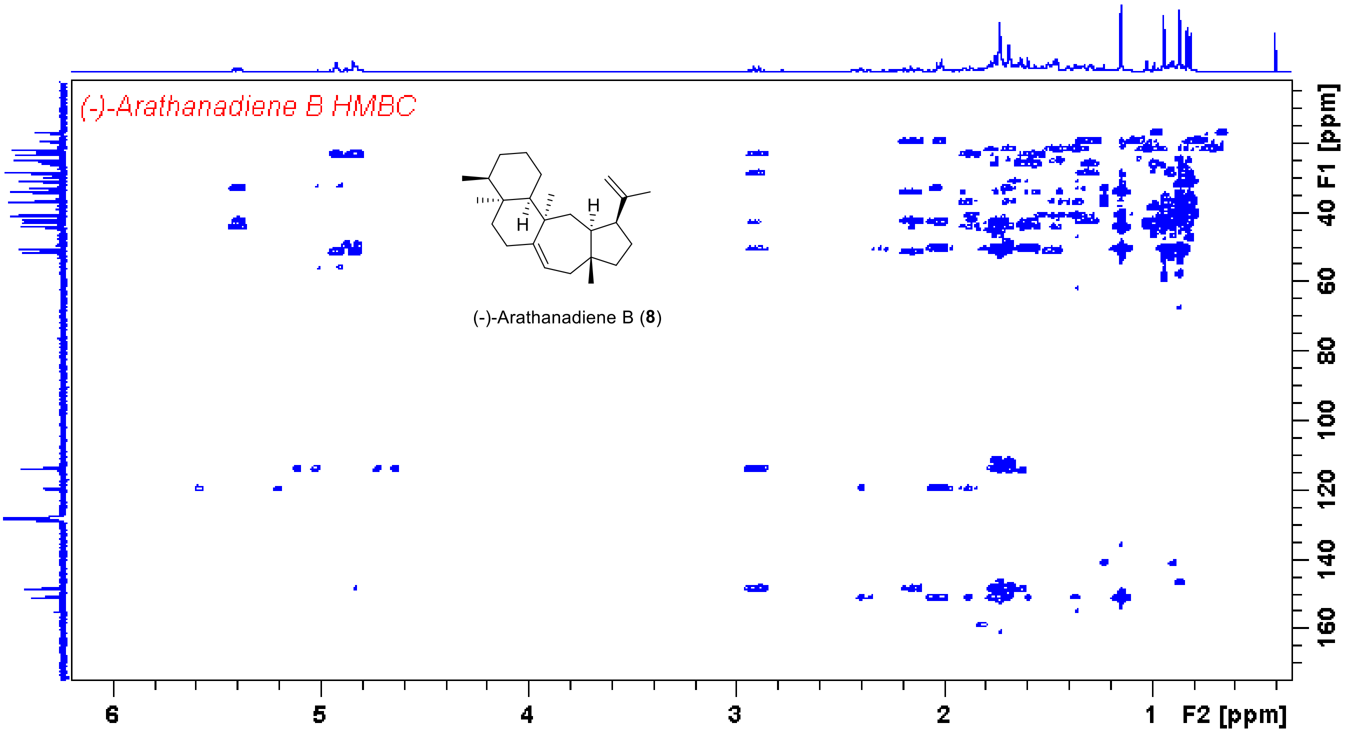

Figure S64. HMBC NMR (400 MHz, benzene-d6) spectrum of (-)-arathanadiene B (**8**)

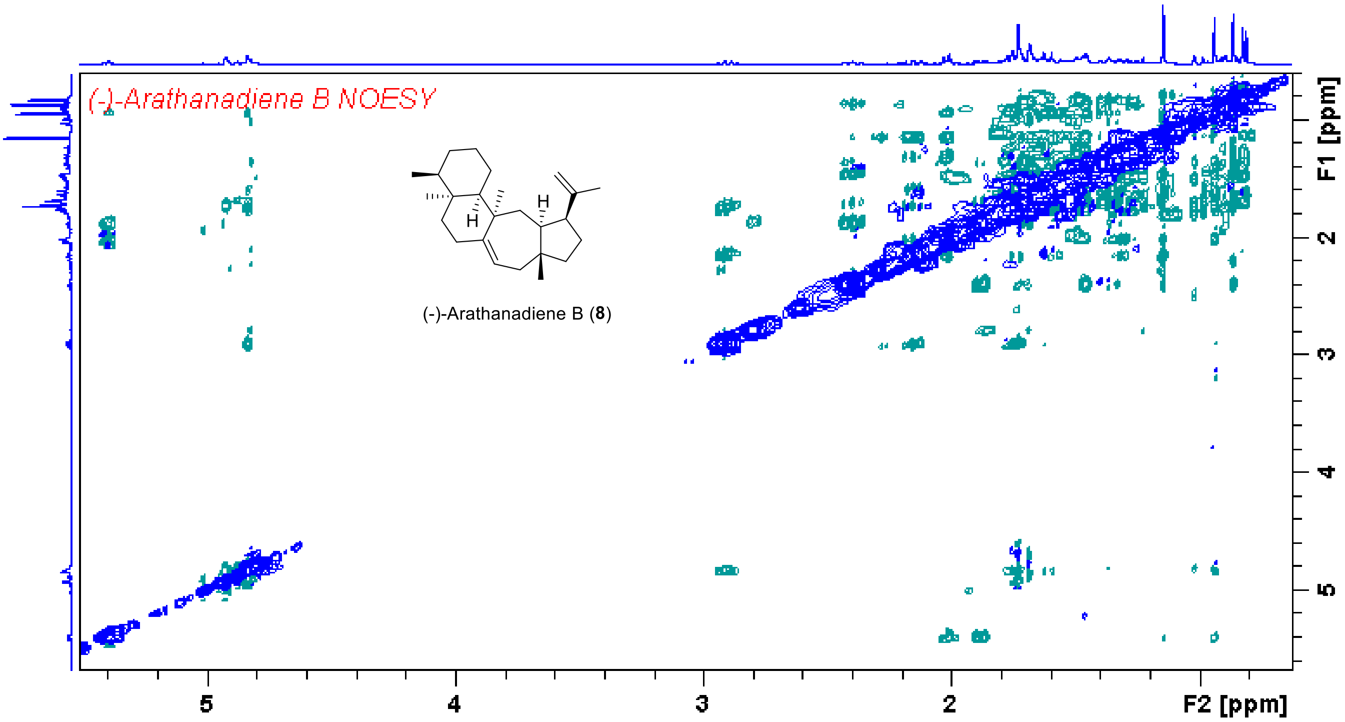

Figure S65. NOESY NMR (400 MHz, benzene-d6) spectrum of (-)-arathanadiene B (**8**)

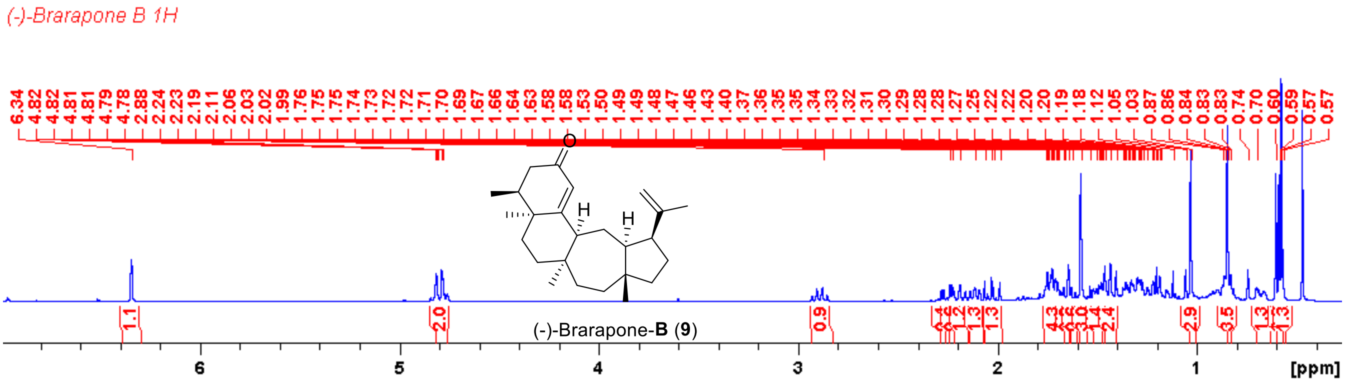

Figure S66. ^{1}H NMR (400 MHz, benzene-d6) spectrum of (-)-brarapone B (**9**)

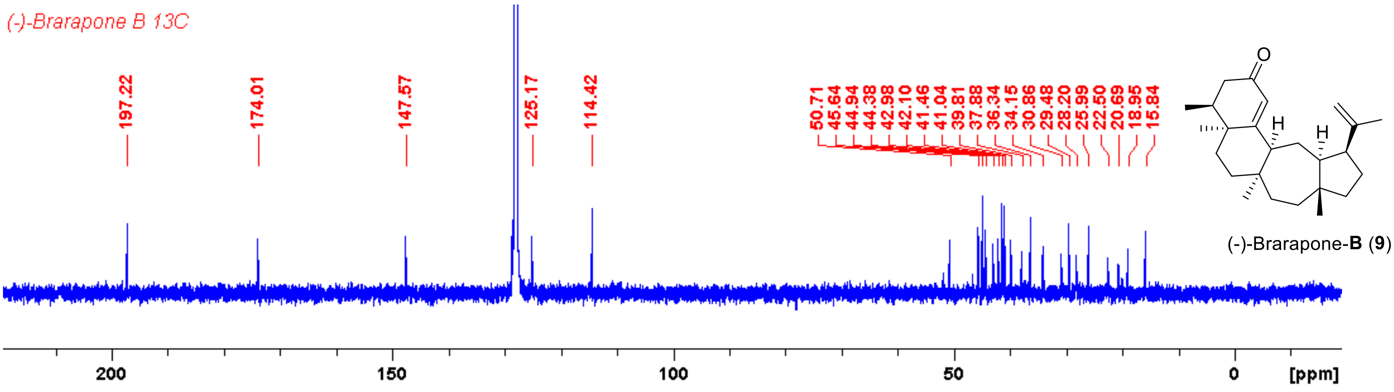

Figure S67. ^{13}C NMR (100 MHz, benzene-d6) spectrum of (-)-brarapone B (**9**)

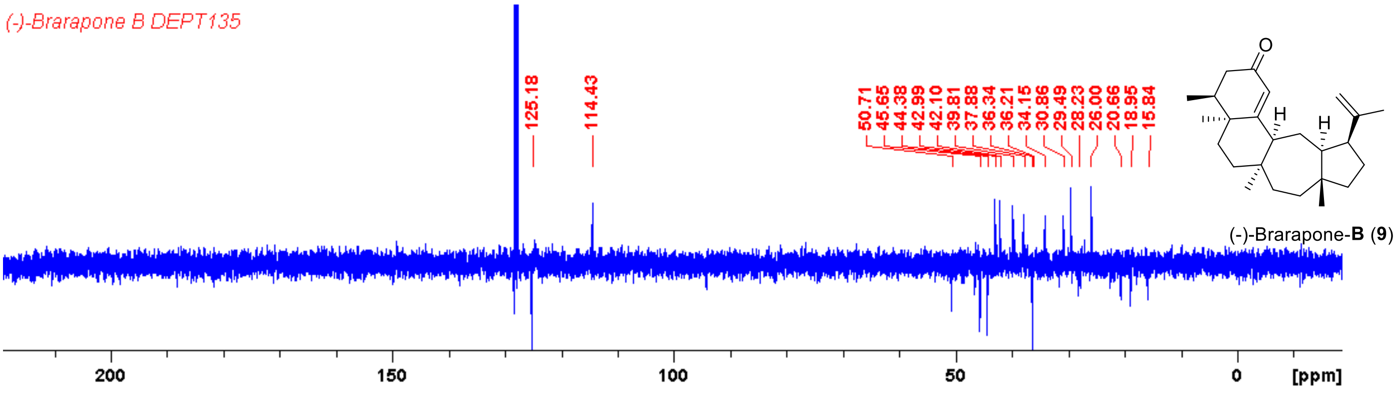

Figure S68. DEPT135 NMR (100 MHz, benzene-d6) spectrum of (-)-brarapone B (**9**)

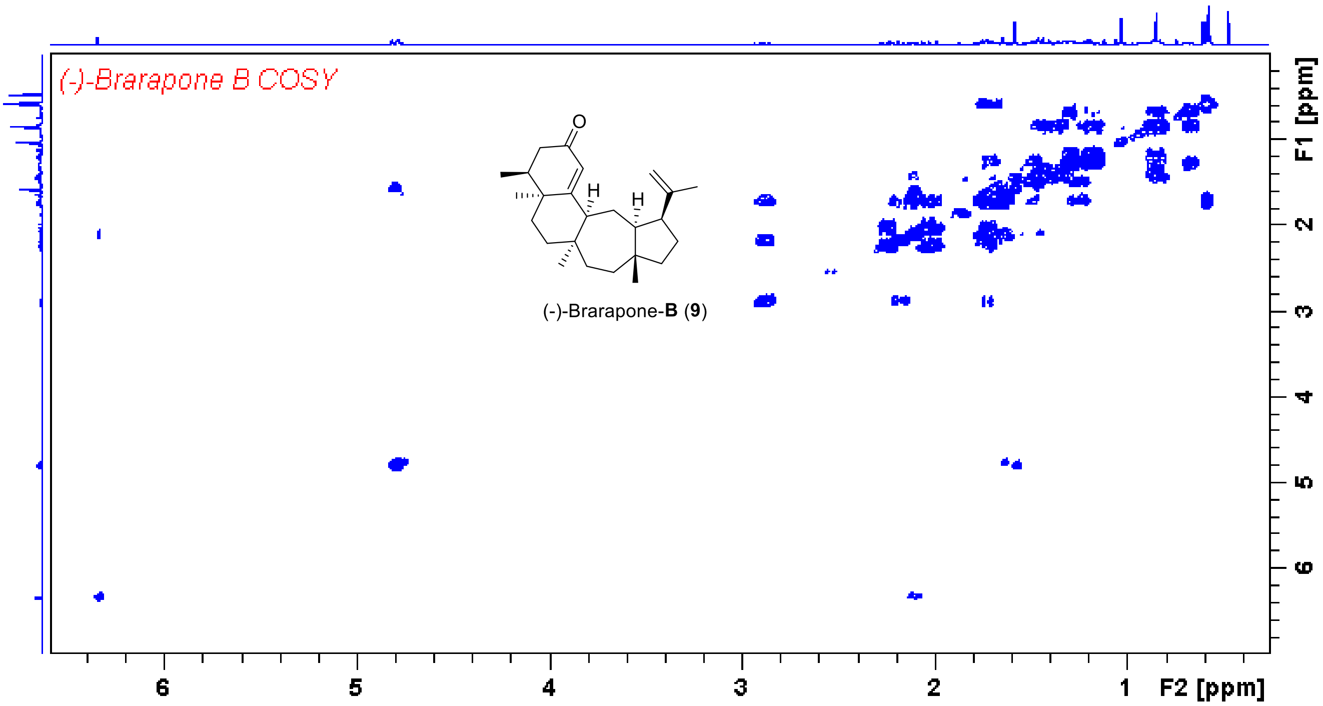

Figure S69. COSY NMR (400 MHz, benzene-d$_6$) spectrum of (-)-brarapone B (**9**)

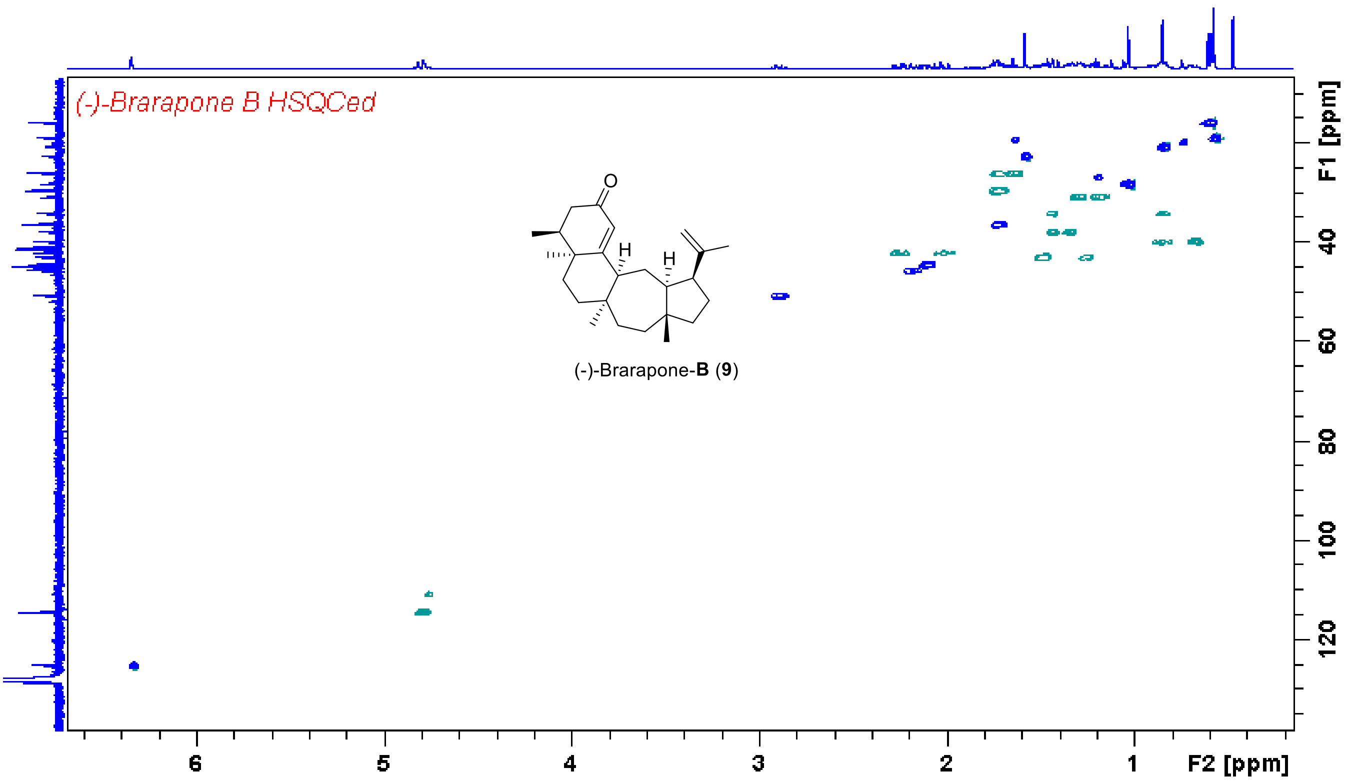

Figure S70. HSQC NMR (400 MHz, benzene-d$_6$) spectrum of (-)-brarapone B (**9**)

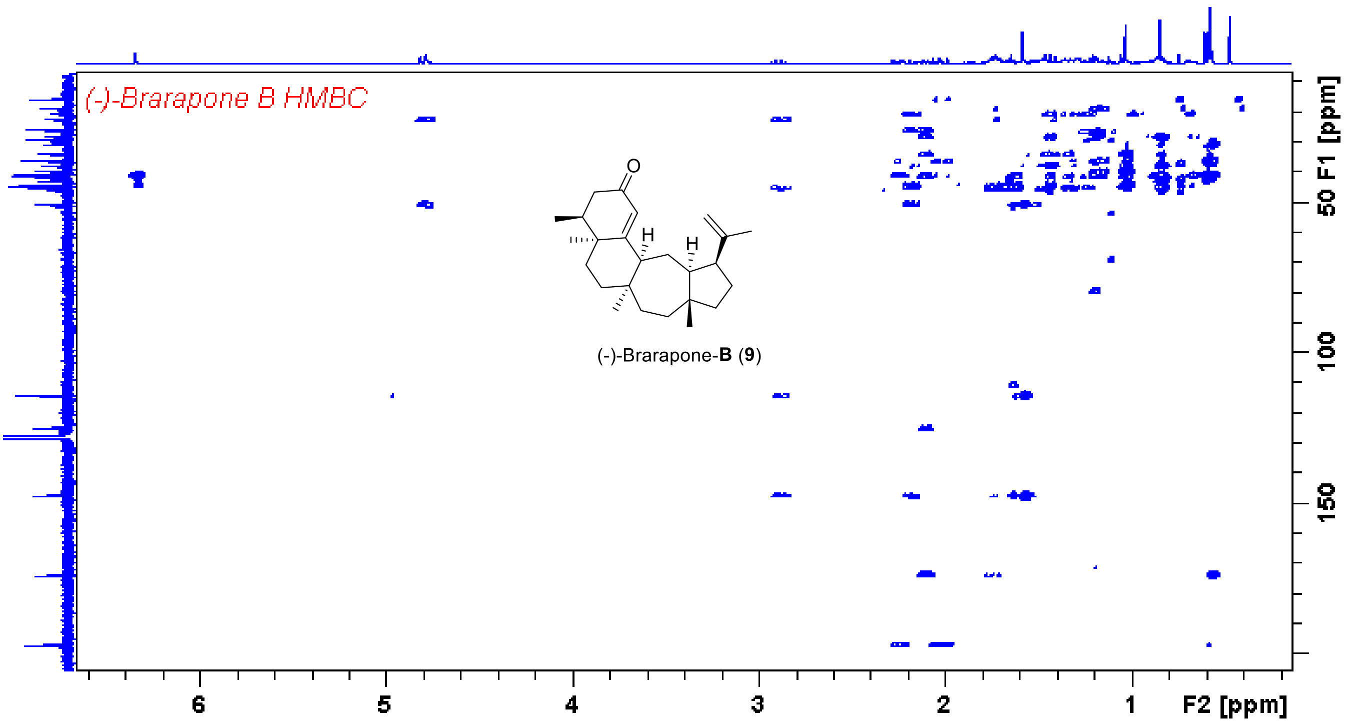

Figure S71. HMBC NMR (400 MHz, benzene-d$_6$) spectrum of (-)-brarapone B (**9**)

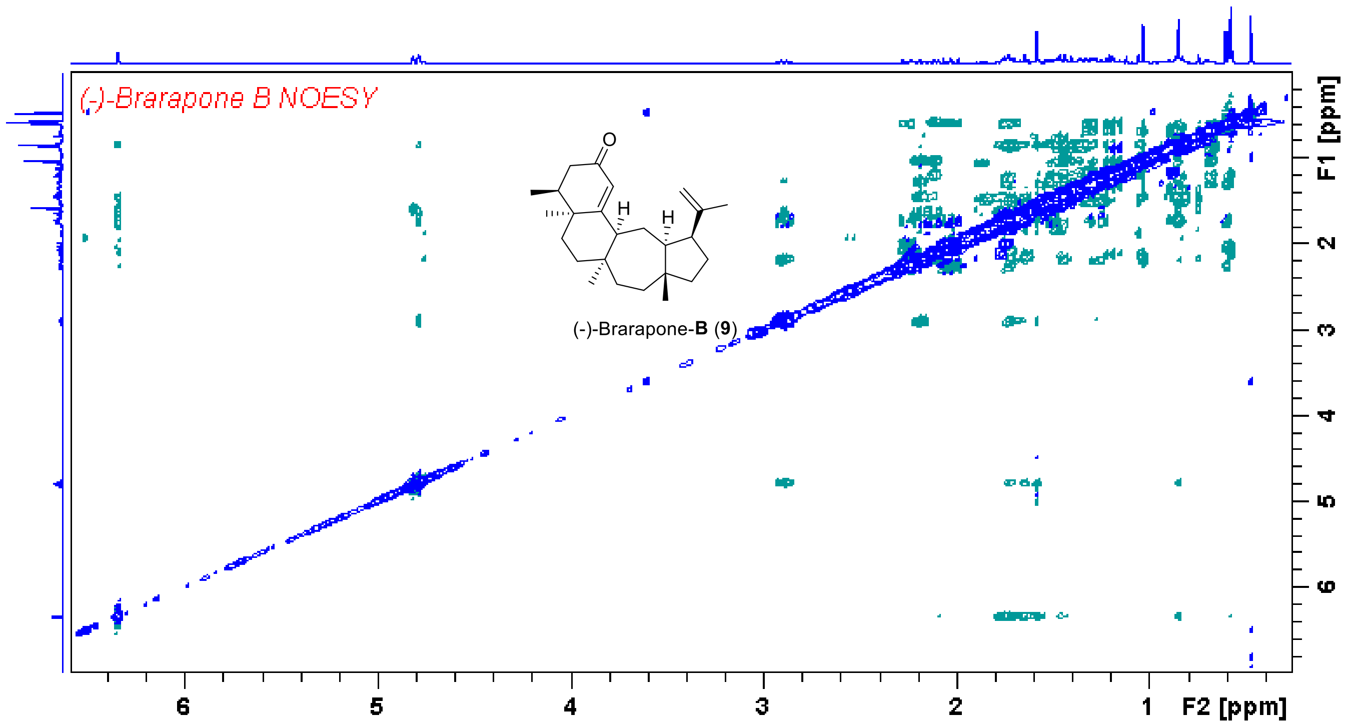

Figure S72. NOESY NMR (400 MHz, benzene-d$_6$) spectrum of (-)-brarapone B (**9**)

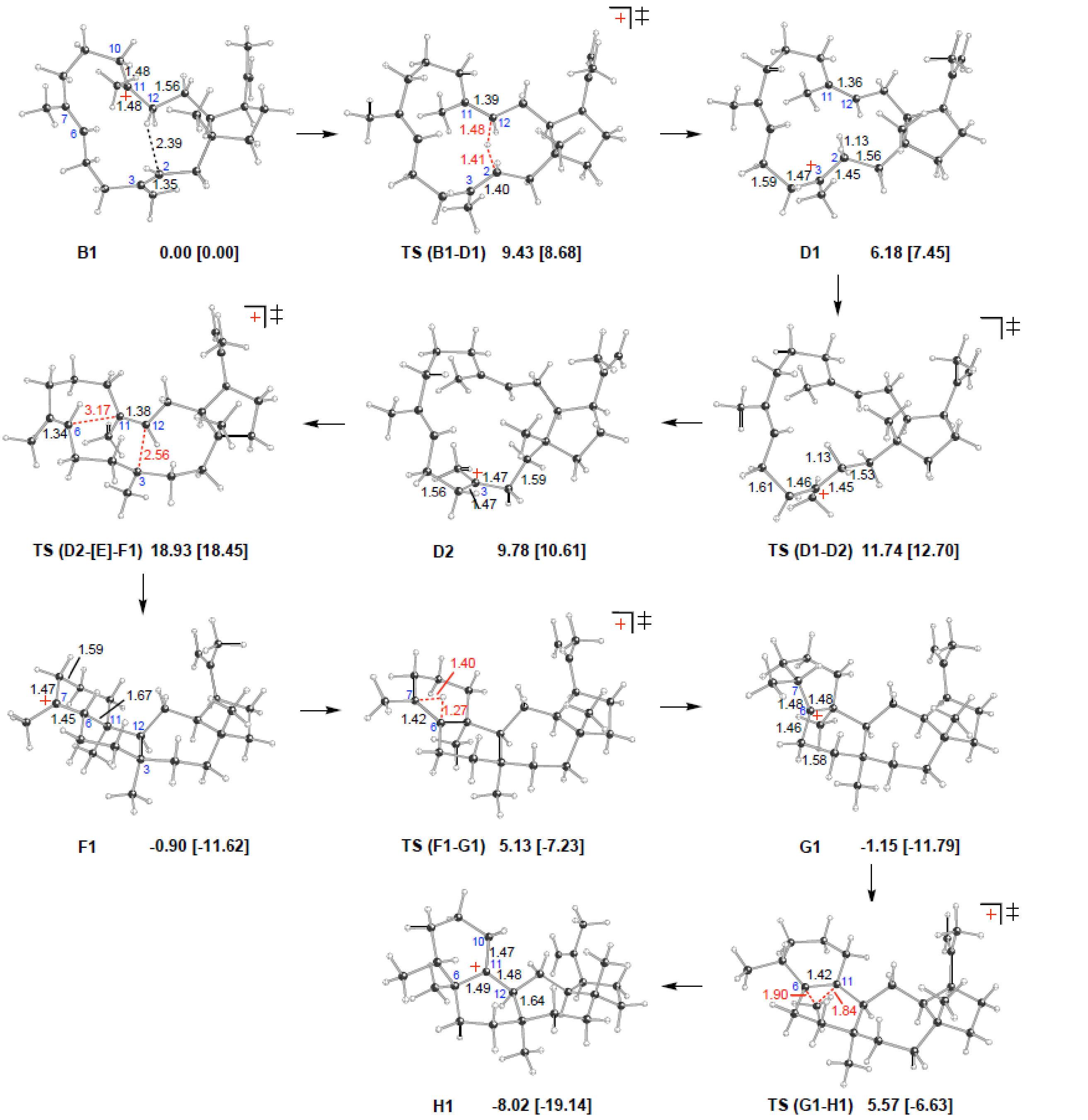

Figure S73. Computed carbocation intermediates and transition state structures involved in the pathway leading to compound **4**. Selected distances (Å) and relative energies (kcal/mol; B3LYP/6-31+G(d,p)) in normal texts and Mpw1pw91/6-31+G(d,p)//B3LYP/6-31+G(d,p) in brackets) are shown.

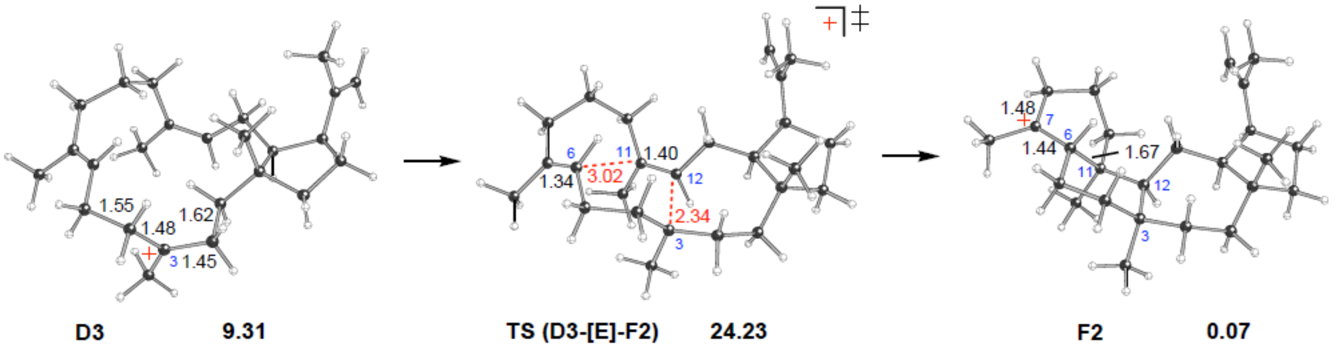

Figure S74. Conversion of a different conformer **D3** to **F2**. Selected distances (Å) and relative energies (kcal/mol; B3LYP/6-31+G(d,p)) in normal texts and Mpw1pw91/6-31+G(d,p)//B3LYP/6-31+G(d,p) in brackets) are shown.
